# A selective removal of the secondary hydroxy group from *ortho*-dithioacetal-substituted diarylmethanols

**DOI:** 10.3762/bjoc.14.105

**Published:** 2018-05-29

**Authors:** Anna Czarnecka, Emilia Kowalska, Agnieszka Bodzioch, Joanna Skalik, Marek Koprowski, Krzysztof Owsianik, Piotr Bałczewski

**Affiliations:** 1 Group of Synthesis of Functional Materials, Centre of Molecular and Macromolecular Studies, Polish Academy of Sciences, Sienkiewicza 112, 90-363 Łódź, Poland,; 2 Department of Structural and Material Research, Institute of Chemistry, Environmental Protection and Biotechnology, Faculty of Mathematics and Natural Sciences, Jan Długosz University in Częstochowa, Armii Krajowej 13/15, 42-200 Częstochowa, Poland

**Keywords:** diarylmethanes, diarylmethanols, 1,3-dithiane, selective reduction, sodium cyanoborohydride, zinc iodide

## Abstract

We present a successful deoxygenation reaction of *ortho*-1,3-dithianylaryl(aryl)methanols which enables a selective removal of the secondary hydroxy group in presence of the 1,3-dithianyl moiety under reductive conditions. This reaction proceeds well with ZnI_2_/Na(CN)BH_3_ in dichloroethane or benzene for both unsubstituted and substituted aryls (by electron-rich groups). This is leading to formyl-protected diarylmethanes with potential application in the synthesis of new pharmaceuticals and optoelectronic materials. This synthetic approach gives an access to a wide variety of functionalized *ortho*-1,3-dithianylaryl(aryl)methanes in 26–95% yields and is recommended for the substrates containing sulfur atoms, for which transition metal-induced reactions fail.

## Introduction

**Figure 1:**
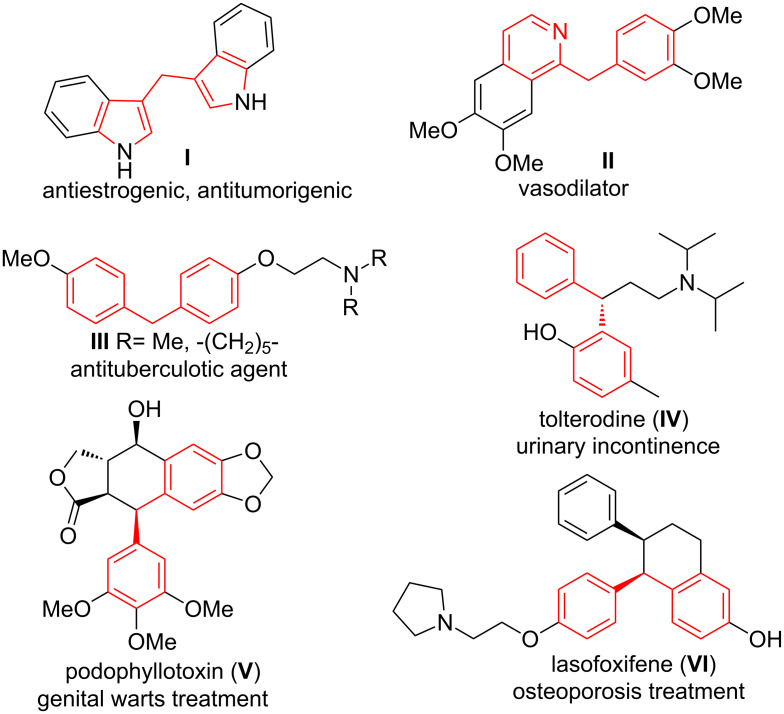
Structures of biologically active diarylmethanes and commercially available pharmaceuticals based on the diarylmethyl moiety.

In last decades, diarylmethanes, such as **I**–**IV** and cyclic compounds possessing a diarylmethyl motif, like podophyllotoxin (**V**) and lasofoxifene (**VI**), have been a subject of interest as bioactive molecules (Figure 1) [1]. The diarylmethane derivatives, isolated from natural sources, showed antibacterial [2], antiestrogenic and antitumorigenic (**I**) [3–5] or vasodilating activities (**II**) [6]. Other ones have been reported as inhibitors of HIV-1 integrase and viral replication in cells [7] or antitubercular agents (**III**) [8–9]. Various diarylmethane-based molecules, like tolterodine (**IV**) [10], podophyllotoxin (**V**) [11] and lasofoxifene (**VI**) [12] have been used in the treatment of overactive bladder, external genital warts and osteoporosis (Figure 1). The diarylmethyl motif also plays a pivotal role in supramolecular architectures of calixarenes [13] and orthocyclophanes [14].

A special, *ortho-*carbonyl-substituted diarylmethyl scaffold is present in the key substrates for aromatic cyclodehydration, also known as the Bradsher reaction [15], which leads to fused, polycyclic aromatic hydrocarbons [16–19]. This type of reaction found applications in synthesis of organic, optoelectronic materials [20–22].

**Scheme 1:**
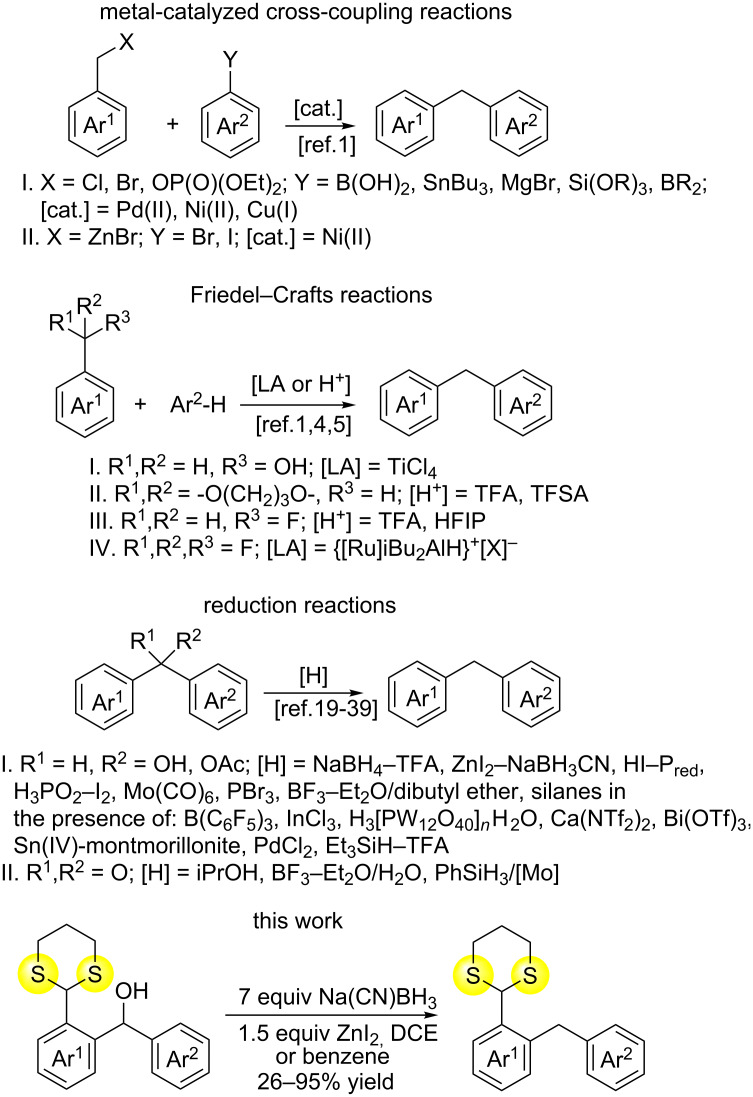
Various synthetic approaches to diarylmethanols (literature review and this work).

Metal-catalyzed cross-coupling reactions (e.g., Suzuki–Miyaura, Stille, Kumada, Hiyama or Negishi couplings) play a key role in the preparation of diarylmethanes. However, the reactions involving sulfur-containing moieties have not been reported yet (Scheme 1) [1]. It is noteworthy that under certain conditions, the Pd-catalyzed cross-coupling reactions of substrates containing an 1,3-dithiane moiety are feasible, like in the case of the 2-arylation of 2-aryl-substituted 1,3-dithianes. However, in the case of 2-benzyl-substituted 1,3-dithianes, a tandem elimination/1,3-dithiane ring opening followed by a Pd-catalyzed C−S bond formation was observed instead [23].

Diarylmethanes were also obtained in the Friedel–Crafts reactions of arenes with primary benzyl alcohols, aryl acetals, and benzyl esters [1]. Benzyl fluorides (in 1,1,1,3,3,3-hexafluoroisopropanol in the presence of a catalytic amount of trifluoroacetic acid [24]) as well as benzotrifluorides (employed in hydrodefluorinative Friedel–Crafts alkylations catalyzed by alumenium ions [25]) were interesting, alternative substrates for alcohols, acetals and esters.

A large group of synthetic procedures for the synthesis of diarylmethanes are deoxygenations of secondary diarylmethyl alcohols with hydride sources. These reactions require a preliminary C–OH bond activation by Brønsted or Lewis acids. Several reagent systems have recently been employed to achieve this goal, including: NaBH_4_–CF_3_COOH [26], ZnI_2_–NaBH_3_CN [27], HI–P_red_ [28], H_3_PO_2_–I_2_ [29–30], Mo(CO)_6_-Lawesson’s reagent [31], PBr_3_ [32], BF_3_·Et_2_O–dibutyl ether [33] and silanes (Si–H) in the presence of various Lewis acids: B(C_6_F_5_)_3_ [34–36], InCl_3_ [37–38], H_3_[PW_12_O_40_]×*n*H_2_O [39], Ca(NTf_2_)_2_ [40], Bi(OTf)_3_ [41], Sn(IV)-montmorillonite [42] and PdCl_2_ [43]. It is interesting that Seto et al. reported that Et_3_SiH–CF_3_COOH, TMSCl/NaI failed to reduce secondary OH groups in diheteroaromatic systems and the reduction with H_2_/Pd/C-ZnBr_2_ was very slow [32]. On the other hand, some rigid diarylmethanols were successfully reduced to benzo[*b*]indeno[2,1-*d*]thiophenes using the Et_3_SiH–CF_3_COOH system which was reported in the patent literature [44].

Benzophenones were also reduced to diarylmethanes using supercritical iPrOH at 350 °C [1], BF_3_·OEt_2_/H_2_O [33] and PhSiH_3_/MoCl_2_O_2_(H_2_O)_2_ [45].

The reductive deoxygenation reactions of diarylmethanols proceed via carbocationic species, which are formed by protonation or complexation of alcohols by Brønsted or Lewis acids. Therefore, diarylmethanols bearing electron-donating substituents (EDG = methyl, methoxy) are reduced much faster than diarylmethanols with electron-withdrawing groups (EWG = CF_3_, C(O)OR) on (hetero)aryl moieties. Consequently, dramatically decreased yields have been observed in these cases [32–33]. It has also been reported that *ortho*-substituents on aryl moieties give lower yields in the reduction processes [33]. Moreover, the OH reduction under acidic conditions cannot be carried out in the presence of other sensitive functional groups. For instance, ether cleavage and dehalogenation (I but not Cl and Br) are common, side reactions due to either the high acidity which is necessary to generate carbocationic species or due to the use of strongly reducing reaction conditions [28–30,46–52]. In this context, a serious challenge is still the selective and direct removal of the OH group from alcohols Ar_2_CH(OH) without excessive, side reactions. Especially in the presence of other functionalities such as, for instance, when a reductively sensitive *ortho*-1,3-dithianyl group is attached to one of the aryl moieties, side products are to be expected. This task is additionally difficult to accomplish due to a chemical similarity of oxygen and sulfur, two neighboring heteroatoms from the main group VI of the periodic table. A longer atomic radius of sulfur than oxygen should make the C–S bond weaker and more reactive than the C–O bond and consequently the reduction of the former should a priori occur preferentially [53–55].

According to the best of our knowledge, there are no literature reports concerning selective reductions of dibenzylic hydroxy groups in the presence of *ortho-*acetal or *ortho*-thioacetal functions. Such reductions may give an access to new series of *ortho*-formyl-protected diarylmethanes as well as their formyl-modified derivatives, obtained after deprotection of the 1,3-dithianyl group with one of the available methods [56].

Herein, we present the first example of a selective and efficient reduction of *ortho*-1,3-dithianylaryl(aryl)methanols leading to *ortho*-1,3-dithianylaryl(aryl)methanes using the ZnI_2_-Na(CN)BH_3_ reductive system (Scheme 1). The use of zinc iodide is critical in this system. It was used for the first time in dichloroethane by Lau et al. to reduce aryl ketones, aldehydes, benzylic, allylic and tertiary alcohols, including the first example of a diarylmethanol (benzhydrol) reduction to diarylmethane (diphenylmethane) [27].

## Results and Discussion

**Scheme 2:**
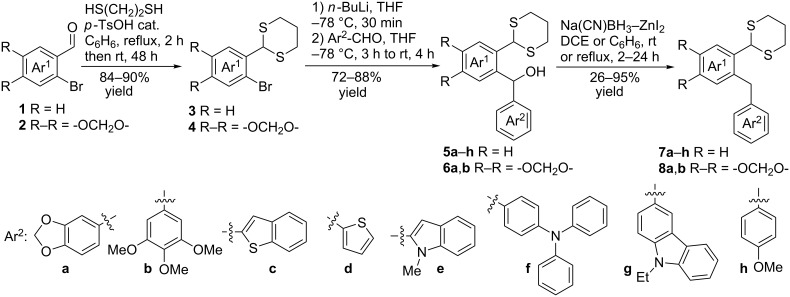
A general strategy for the synthesis of *ortho*-1,3-dithianylaryl(aryl)methanols **5** and **6**, and their reduction to the corresponding *ortho*-1,3-dithianylaryl(aryl)methanes.

The synthesis of *ortho*-1,3-dithianylaryl(aryl)methanols **5** and **6**, as key substrates for the OH reduction, has been realized according to the procedure shown in Scheme 2 including: 1) protection of the formyl group in *ortho*-bromoaldehydes **1** and **2** with 1,3-propanedithiol, 2) the Br/Li exchange reaction in the resulting *ortho*-bromo-1,3-dithianes **3** and **4** with *n*-BuLi followed by condensation with a second (hetero)aromatic aldehyde Ar^2^-CHO.

The reduction of *ortho*-1,3-dithianylaryl(aryl)methanols **5** and **6** to *ortho*-1,3-dithianylaryl(aryl)methanes **7** and **8** has been carried out using Lau’s procedure with ZnI_2_ (1.5 equiv) and Na(CN)BH_3_ (7 equiv) in dichloroethane (DCE) and a solvent-modified protocol employing the same reagents in benzene. In literature, only few applications of the ZnI_2_-Na(CN)BH_3_ system in DCE have been described for reduction of diarylmethanols that do not contain acid-sensitive, formyl protecting, acetal or thioacetal groups [20,27,57–60] and for the reduction of aromatic aldehydes and ketones [28]. The bromo function in such diarylmethanols [60], as well as Cl, Br, C(O)OMe, MeO, MeS and NO_2_ groups in aromatic aldehydes and ketones, being reduced to methyl and methylene groups, respectively [27], are tolerated by this reagent system.

The use of other procedures for deoxygenation of alcohols **5d**, **6a** and **6b** with metal hydride donors, such as NaBH_4_ and LiAlH_4_ in various combinations with ZnI_2_ and AlCl_3_ failed [61–63]. For instance, the reaction with NaBH_4_ itself and NaBH_4_/ZnI_2_/THF at room temperature and reflux only recovered the substrates. The same result was obtained at room temperature with NaBH_4_/AlCl_3_/THF, while at reflux the whole substrate was consumed and three unidentified products were formed lacking the SCHS and ArCH_2_Ar characteristic signals in ^1^H NMR spectra. With NaBH_4_/CF_3_COOH/rt, the substrate underwent decomposition.

In our case, the use of the Lau’s procedure in DCE for the reductive deoxygenation of *ortho*-1,3-dithianyl-substituted alcohols **5a**–**h** containing unsubstituted Ar^1^ phenyl ring and electron-donating Ar^2^ groups, gave diarylmethanes **7a**–**h** in 26–95% yields (Table 1, entries 1–9).

**Table 1:** A selective reduction of *ortho-*1,3-dithianylaryl(aryl)methanols to *ortho-*1,3-dithianylaryl(aryl)methanes using the ZnI_2_/Na(CN)BH_3_ reducing system.

entry	substrate	solvent	time [h]	temperature [°C]	product	yield

**1**	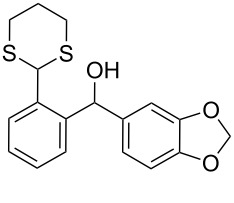 **5a**	DCE	24	rt	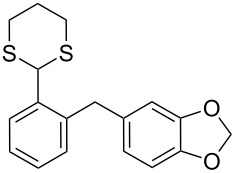 **7a**	95
**2**	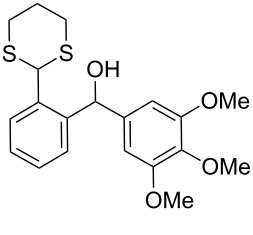 **5b**	DCE	72	rt	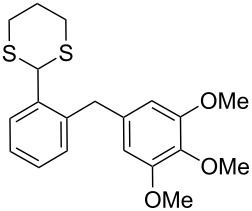 **7b**	95^a^
**3**		DCE	72	reflux		85
**4**	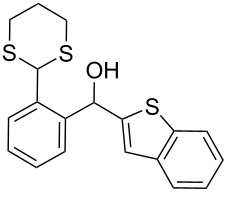 **5c**	DCE	72	rt	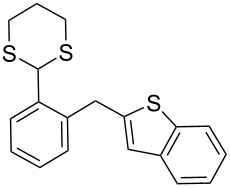 **7c**	70
**5**	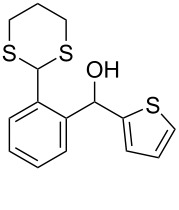 **5d**	DCE	2	rt	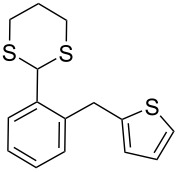 **7d**	95
**6**	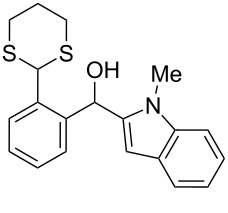 **5e**	DCE	2	rt	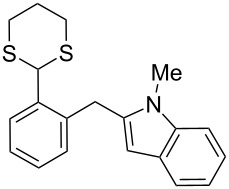 **7e**	26
**7**	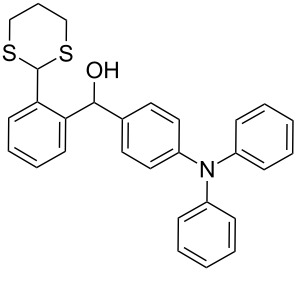 **5f**	DCE	1	rt	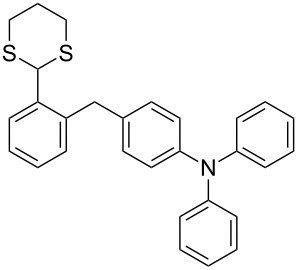 **7f**	95
**8**	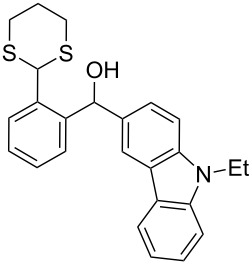 **5g**	DCE	3	rt	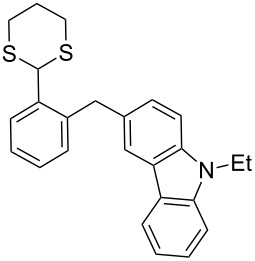 **7g**	59
**9**	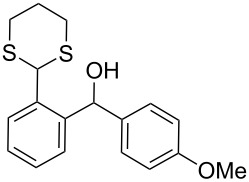 **5h**	DCE	5	rt	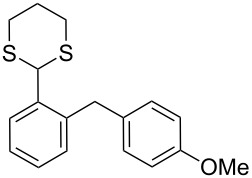 **7h**	64
**10**	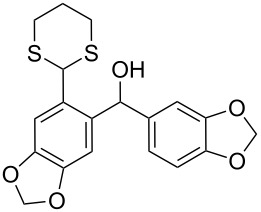 **6a**	C_6_H_6_	24	rt	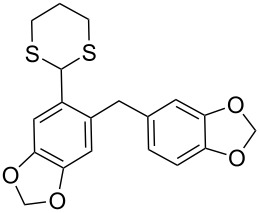 **8a**	95
**11**	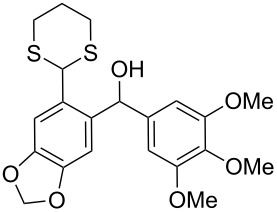 **6b**	C_6_H_6_	72	rt	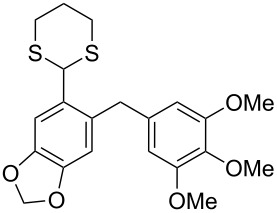 **8b**	95^a^
**12**		C_6_H_6_	24	reflux		60

^a^Yield calculated based on the consumed substrate.

**Scheme 3:**
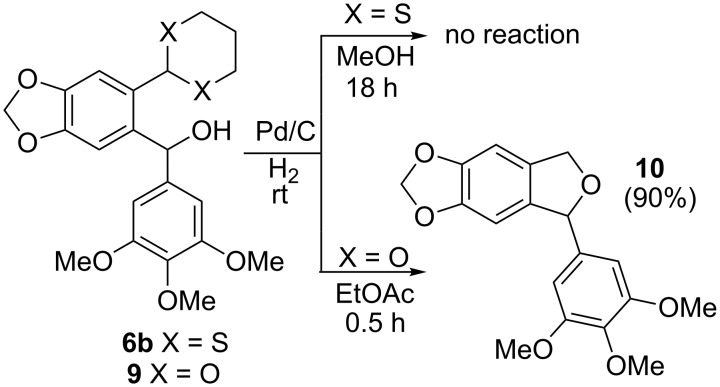
Attempts of the OH removal in *ortho*-1,3-dithianyl- **6b** and *ortho*-1,3-dioxanylaryl(aryl)methanols **9** using the Pd-catalyzed hydrogenolysis reaction.

Surprisingly, the reduction of the Ar^1^ piperonyl series **6a**,**b** failed under the same reaction conditions and only the substrates could be isolated. In this case, a replacement of DCE by benzene resulted in a successful formation of the desired diarylmethanes **8a**,**b** in 95% yield (Table 1, entries 10–12). In addition, diarylmethanols **5b** and **6b** with the bulky group Ar^2^ = 3,4,5-trimethoxyphenyl required refluxing in the relevant solvent (benzene or DCE) to complete the reduction (Table 1, entries 3 and 12). At room temperature, only 40% of the substrates underwent conversion to diarylmethanes **7b** and **8b** due to their low solubility at this temperature. Attempts to synthesize diarylmethanols **6** with electron-withdrawing groups (Ar^2^ = *p*-NO_2_, *p*-CF_3_) were also unsuccessful. They proved to be unstable: the reaction of lithiated **4** with *p*-nitro and *p*-trifluoromethylbenzaldehyde gave five products in the crude reaction mixture and even more complex mixtures of products after silica gel column chromatography. We also tried to remove the OH group using the Pd-catalytic hydrogenolysis (Scheme 3).

In case of **6b** (X = S), no reaction occurred and only the substrate was recovered, most probably due to poisoning of the catalyst by the 1,3-dithianyl sulfur atoms. However, the successful catalytic hydrogenolysis (5% Pd/C) of diphenylmethanol to diphenylmethane, described in literature [64], encouraged us to try the Pd-catalyzed reduction of the oxygen analog **9** (X = O) as a representative of the *orth*o-1,3-dioxanyl series. In this case, instead of the expected *ortho*-1,3-dioxanyl diarylmethane analog of **8b**, 1,3-dihydroisobenzofuran **10** was obtained in 90% yield, within 30 min, as a result of preferential deacetalization over the dibenzylic OH deoxygenation. This is followed by cyclization and final deoxygenation of the secondary monobenzylic OH group in the resulting lactol. This product is known in literature and was obtained by hydrogenolysis or pyrolysis of the corresponding *ortho*-1,3-dioxolanyl [65] and *ortho*-hydroxymethyl [66] substituted diarylmethanols, respectively. It is worth mentioning that the reduction of **9** with ZnI_2_–Na(CN)BH_3_ caused decomposition of the starting material. The described reduction process involving 1,3-dithianyl derivatives is facilitated by oxophilic zinc which preferentially binds to the OH oxygen atoms (bond dissociation energies for Zn–O and Zn–S are 284 and 205 kJ/mol, respectively) [67]. The weakened C–O bond is thus more susceptible to the borohydride attack to produce the corresponding diarylmethanes. Most probably, electron-donating groups on aryls facilitate both Zn complexation and stabilization of the intermediate carbocationic species formed.

## Conclusion

In summary, a new example of the selective functional group transformation of diarylmethanols (Ar^1^Ar^2^CH(OH)) to diarylmethanes (Ar^1^Ar^2^CH_2_) has been performed. It is important for the sulfur-containing substrates for which metal-catalyzed cross-coupling reactions fail. The dibenzylic OH group has been preferentially reduced with the ZnI_2_/Na(CN)BH_3_ system in the presence of an *ortho*-dithioacetal moiety to give *ortho*-1,3-dithianylaryl(aryl)methanes in 26–95% yields under mild reaction conditions. Thus, the *ortho*-1,3-dithianyl moiety joins other functionalities tolerated by this reagent system [47]. Since analogous 1,3-dioxanyl derivatives, in our hands, decomposed or led to other products during the attempted catalytic hydrogenolysis, the elaborated protocol is the only one up to date that enables synthesis of *ortho*-formyl-protected diarylmethane derivatives under reductive conditions. The latter may be used as key substrates both for the synthesis of biologically active diarylmethanes or for the synthesis of (hetero)acenes via the Bradsher protocol.

## Supporting Information

File 1 General experimental information, characterization data and copies of ^1^H, ^13^C NMR spectra.
