# Supplementary material for: A selective removal of the secondary hydroxy group from ortho-dithioacetal-substituted diarylmethanols
Source: Beilstein J Org Chem. 2018 May 29;14:1229–37. doi: 10.3762/bjoc.14.105 (PMC6009171; doi:10.3762/bjoc.14.105)

Supporting Information

for

A selective removal of the secondary hydroxy group from *ortho*-dithioacetal-substituted diarylmethanols

Anna Czarnecka,[1] Emilia Kowalska,[1] Agnieszka Bodzioch,[1] Joanna Skalik,[1] Marek Koprowski,[1] Krzysztof Owsianik[1] and Piotr Bałczewski*[1,2]

Address: [1]Group of Synthesis of Functional Materials, Centre of Molecular and Macromolecular Studies, Polish Academy of Sciences, Sienkiewicza 112, 90-363 Łódź, Poland, and [2]Department of Structural and Material Research, Institute of Chemistry, Environmental Protection and Biotechnology, Faculty of Mathematics and Natural Sciences Jan Długosz University in Częstochowa, Armii Krajowej 13/15, 42-200 Częstochowa, Poland

Email: Piotr Bałczewski - pbalczew@cbmm.lodz.pl

*Corresponding author

General experimental information, characterization data and copies of ^{1}H, ^{13}C NMR spectra

Table of Contents

1. Experimental procedures.. S2

2. Copies of ^{1}H and ^{13}C NMR spectra.. S15

1. Experimental procedures

Experimental details. ^{1}H NMR (200 or 500 MHz) and ^{13}C NMR (50 or 125 MHz) spectra were recorded with a Bruker AV 200 or DRX 500 spectrometers at ambient temperature. The mass spectra of pure compounds were obtained using a Finnigan MAT 95 spectrometer. Melting points were determined using a Boetius apparatus. Column chromatography was performed on Merck silica gel 60 (F254, 270–400 mesh). The starting substrates **1** and **2** are commercially available and were obtained from Sigma-Aldrich.

General procedure for preparation of dithioacetals 3 and 4: To a solution of the aldehyde **1** or **2** (0.1 mol) in benzene (300 mL) were added *p*-TsOH (10 mol %) and 1,3-propanedithiol (0.11 mol). The resulting mixture was stirred at 80 °C for 2 h, and at room temperature for 2 days. Then, the crude mixture was diluted with diethyl ether (150 mL), washed with aqueous solution of 2 M NaOH (50 mL) and H_2O (3 × 50 mL), and dried over anhydrous $MgSO_4$. After removal of the solvent, the crude product was recrystallized from the mixture of benzene/hexane (1:1, v/v) to give analytically pure **3** or **4**.

2-(2-Bromophenyl)-1,3-dithiane (3) [1]: By following the general procedure **3** was obtained from aldehyde **1** in 90% yield, 24,7 g; m.p.: 96-98 °C. ^{1}H NMR (200 MHz, $CDCl_3$): δ = 7.65 (d, J = 7.5 Hz, 1 H) 7.49 (d, J = 7.5 Hz, 1 H), 7.26 (dd, J = 7.5, 7.5 Hz, 1 H), 7.06 (dd, J = 7.5, 7.5 Hz, 1 H), 5.56 (s, 1 H), 3.08-2.71 (m, 4 H), 2.10-1.71 (m, 2 H). ^{13}C NMR (50 MHz, $CDCl_3$): δ = 137.0, 131.7, 128.6 (2 C), 126.9, 121.8, 23.9, 31.0 (2 C), 49.4. MS (EI, 70 eV): *m/z* (%): 274 [M^+, 75], 200 [M^+, -SC_3H_6, 90], 195 [M^+, -Br, 34], 121 [M^+, -$SCH_2CH_2CH_2$, -Br, 100]. HRMS (EI): *m/z* [M^+] calcd. for

$C_{10}H_{11}BrS_2$ 273.9485; found 273.9480. Anal. calcd for $C_{10}H_{11}BrS_2$: C, 43.64; H, 4.03; Br, 29.03; S, 23.30; found: C, 43.75; H, 4.07; Br, 29.57; S, 23.32.

5-Bromo-6-(1,3-dithian-2-yl)-1,3-benzodioxole (4) [2]: By following the general procedure **4** was obtained from aldehyde **2** in 84% yield, 26,7 g; M.p.: 104-106 °C. ^{1}H NMR (200 MHz, $CDCl_3$): δ = 7.16 (s, 1 H), 6.96 (s, 1 H), 5.96 (s, 2 H), 5.51 (s, 1 H), 3.16-3.02 (m, 2 H), 2.93-2.82 (m, 2 H), 2.21-2.08 (m, 1 H), 1.99-1.77 (m, 1 H). ^{13}C NMR (50 MHz, $CDCl_3$): δ = 147.0, 146.6, 130.0, 112.3, 111.3, 108.1, 100.7, 49.3, 31.0 (2 C), 23.9. MS (CI, isobutane): *m/z* (%): 320 [M^+, 100], 246 [M^+, -$SCH_2CH_2CH_2$, 18], 239 [M^+, -Br, 46], 213 [M^+, -$SCH_2CH_2CH_2S$, 28]. HRMS (EI): *m/z* [M^+] calcd for $C_{11}H_{11}BrO_2S_2$: 317.9384; found: 317.9385. Anal. calcd for $C_{11}H_{11}BrO_2S_2$: C, 41.39; H, 3.47; S, 20.09; found: C, 41.49; H, 3.52; S, 20.42.

General procedure for preparation of *ortho*-1,3-dithianylaryl(aryl)methanols 5a–h and 6a,b [3]: To a solution of dithioacetals **3** or **4** (1 mmol) in dry THF (20 mL), cooled to –78 °C, was added *n*-BuLi (1.4 mmol, 2.7 M solution in hexanes). The resulting mixture was stirred at this temperature for 30 min under argon atmosphere. Then, the corresponding aldehyde (Ar^2-CHO) (1.1 mmol) in THF (10 mL) was added. The reaction mixture was stirred for 3 h at –78 °C, warmed to room temperature and stirred for 4 h at this temperature. The mixture was diluted with ethyl acetate (25 mL), washed with a saturated aqueous solution of NH_4Cl (30 mL), and then water (20 mL). The organic layer was dried over anhydrous $MgSO_4$, filtered and concentrated under reduced pressure. The crude product was purified using a silica gel column chromatography with 10% acetone in petroleum ether as eluent.

(2-(1,3-Dithian-2-yl)phenyl)(benzo[*d*][1,3]dioxol-5-yl)methanol (5a): By following the general procedure **5a** was obtained as a white solid from dithioacetals **3** in 82% yield, 0.284 g; m.p.: 142-143 °C. ^{1}H NMR (500 MHz, C_6D_6): δ = 7.89 (d, *J* = 7.3 Hz, 1

H), 7.20 (d, J = 7.3 Hz, 1 H), 7.01 (s, 1 H), 6.98 (dd, J = 7.5, 7.5, 1 H), 6.92 (dd, J = 7.5, 7.5, 1 H), 6.79 (d, J = 8.0 Hz, 1 H), 6.59 (d, J = 8.0 Hz, 1 H), 6.04 (s, 1 H), 5.50 (s, 1 H), 5.21 (d, J = 3.1 Hz, 2 H), 2.45-2.38 (m, 2 H), 2.33-2.18 (m, 2 H), 2.13 (d, J = 4.1 Hz, 1 H), 1.56-1.47 (m, 1 H), 1.31-1.26 (m, 1 H). ^{13}C NMR (125 MHz, C_6D_6): δ = 149.0. 148.0, 142.1, 138.5, 138.4 130.1 129.4, 129.3, 129.3, 121.1, 108.9, 108.5, 101.6, 73.6, 49.3, 33.1 (2 C) 25.9. MS (EI, 70 eV) *m/z* (%): 346 [M^+, 1], 240 [M^+, -$S_2C_3H_6$, 100], 239 [M^+, -$HS_2C_3H_6$, 74], 210 [M^+, -$S_2C_4H_7$, -OH, 34]. HRMS (EI): *m/z* [M^+] calcd for $C_{18}H_{18}O_3S_2$: 346.0697; found: 346.0704. Anal. calcd for $C_{18}H_{18}O_3S_2$: C, 62.40; H, 5.24; S, 18.51; found: C, 62.31; H, 5.27; S, 18.68.

(2-(1,3-Dithian-2-yl)phenyl)(3,4,5-trimethoxyphenyl)methanol (5b): By following the general procedure **5b** was obtained as a white solid from dithioacetals **3** in 79% yield, 0.310 g; m.p.: 103-105 °C. ^{1}H NMR (500 MHz, C_6D_6): δ = 7.94 (d, J =7.5 Hz, 1 H), 7.29 (d, J = 7.5 Hz, 1 H), 7.00 (dd, J = 7.5, 7.5 Hz, 1 H), 6.97 (dd, J = 7.5, 7.5 Hz, 1 H), 6.74 (s, 2 H), 6.21 (s, 1 H), 5.59 (s, 1 H), 3.79 (s, 3 H), 3.37 (s, 6 H), 2.60 (s, 1 H), 2.50-2.38 (m, 2 H), 2.31-2.15 (m, 2 H), 1.60-1.49 (m, 1 H), 1.34-1.29 (m, 1 H). ^{13}C NMR (125 MHz, C_6D_6): δ = 154.8 (2 C), 142.3, 139.6, 139.1, 138.6, 130.2, 129.6, 129.5 (2 C), 105.2 (2 C), 73.7, 61.2, 56.5 (2 C), 49.4, 33.1 (2 C), 25.9. MS (EI, 70 eV) *m/z*: 392 [M^+, 11], 286 [M^+, -$S_2C_3H_6$, 66], 285 [M^+, -$HS_2C_3H_6$, 44], 284 [M^+, -$H_2S_2C_3H_6$, 69], 255 [M^+, -$S_2C_4H_7$, -H_2O, 100]. HRMS (EI): *m/z* [M^+] calcd for $C_{20}H_{24}O_4S_2$: 392.1116; found: 392.1119. Anal. calcd for $C_{20}H_{24}O_4S_2$: C, 61.20; H, 6.16; S, 16.34; found: C, 61.18; H, 6.01; S, 16.07.

(2-(1,3-Dithian-2-yl)phenyl)(benzo[*b*]thien-2-yl)methanol (5c): By following the general procedure **5c** was obtained as a slightly yellow foam from dithioacetals **3** in 74% yield, 0.265 g; m.p.: 67-69 °C. ^{1}H NMR (500 MHz, C_6D_6): δ = 7.95 (d, J = 7.6 Hz, 1 H), 7.53 (d, J= 7.6 Hz, 1 H), 7.43 (d, J = 7.6 Hz, 1 H), 7.36 (d, J = 7.6 Hz, 1 H),

7.12-7.04 (m, 2 H), 7.04-6.96 (m, 2 H), 6.98 (s, 1 H), 6.40 (s, 1 H), 5.59 (s, 1 H), 2.90 (s, 1 H), 2.45 (dd, *J* = 13.3, 13.3 Hz, 1 H), 2.37 (dd, *J* = 13.3, 13.3 Hz, 1 H) 2.25-2.13 (m, 2 H), 1.59-1.45 (m, 1 H), 1.37-1.22 (m, 1 H). ^{13}C NMR (125 MHz, C_6D_6): δ = 149.5, 141.1, 141.1, 140.9, 138.5, 130.3, 129.9, 129.5, 129.4, 125.1, 125.0, 124.6, 123.3, 122.6, 71.6, 49.6, 33.0 (2 C), 25.8. MS (EI, 70 eV) *m/z*: 358 [M^+, 7], 252 [M^+, -$S_2C_3H_6$, 45], 251 [M^+, -$HS_2C_3H_6$, 77], 250 [M^+, -$H_2S_2C_3H_6$, 100], 234 [M^+, -$S_2C_3H_6$, -H_2O, 31], 221 [M^+, - $S_2C_4H_7$, -H_2O, 29]. HRMS (EI): *m/z* [M^+] calcd for $C_{19}H_{18}OS_3$: 358.0520; found: 358.0521. Anal. calcd for $C_{19}H_{18}OS_3$: C, 63.65; H, 5.06; S, 26.83; found: C, 63.38; H, 4.91; S, 26.59.

2-(1,3-Dithian-2-yl)phenyl(thien-2-yl)methanol (5d): By following the general procedure **5d** was obtained as a yellow oil from dithioacetals **3** in 77% yield, 0.237 g. ^{1}H NMR (500 MHz, C_6D_6): δ = 7.87 (d, *J* =7.5 Hz, 1 H), 7.31 (d, *J* = 7.5 Hz, 1 H), 6.99 (dd, *J* = 7.5, 7.5 Hz, 1 H), 6.93 (dd, *J* =7.5, 7.5 Hz, 1 H), 6.84 (d, *J* = 6.8 Hz, 1 H), 6.71-6.67 (m, 1 H), 6.63 (dd, *J* = 4.9, 3.6 Hz, 1H), 6.29 (s, 1 H), 5.47 (s, 1 H), 2.73 (s, 1H), 2.45-2.35 (m, 2 H), 2.26-2.13 (m, 2 H), 1.57-1.43 (m, 1 H), 1.37-1.21 (m, 1 H). ^{13}C NMR (125 MHz, C_6D_6): δ = 148.8, 141.6, 138.1, 130.2, 129.7, 129.5, 127.7, 125.9 (3 C), 71.1, 49.4, 33.00 (2 C), 25.9. MS (EI, 70 eV) *m/z*: 308 [M^+, 1], 290 [M^+, -H_2O, 7], 243 [M^+, -SCH_4, -OH, 10], 216 [M^+, -SC_3H_6, -H_2O, 27], 202 [M^+, -$S_2C_4H_6$, 80], 201 [M^+, -$HS_2C_3H_6$, 59], 200 [M^+, -$H_2S_2C_3H_6$, 93], 184 [M^+, -$S_2C_3H_6$, -H_2O, 49], 171 [M^+, -$S_2C_4H_7$, -H_2O, 100]. HRMS (EI): *m/z* [M^+] calcd for $C_{15}H_{16}OS_3$: 308.0363; found: 308.0361. Anal. calcd for $C_{15}H_{16}OS_3$: C, 58.40; H, 5.23; S, 31.18; found: C, 58.61; H, 5.31; S, 31.23.

(2-(1,3-Dithian-2-yl)phenyl)(1-methyl-1*H*-indol-2-yl)methanol (5e): By following the general procedure **5e** was obtained as a yellow solid from dithioacetals **3** in 76% yield, 0.270 g; m.p.: 170-172 °C. ^{1}H NMR (500 MHz, C_6D_6): δ = 7.96 (d, *J* = 7.5 Hz,

1 H), 7.60 (d, *J* = 7.5 Hz, 1 H), 7.21-7.10 (m, 2 H), 7.03 (dd, *J* = 7.5, 7.5 Hz, 1 H), 7.00 (dd, *J* = 7.5, 7.5 Hz, 1 H), 6.91 (dd, *J* = 7.5, 7.5 Hz, 1 H), 6.43 (s, 1 H), 6.19 (d, *J* = 5.0 Hz, 1 H), 5.63 (s, 1 H), 3.17 (s, 3 H), 2.40 (dd, *J* = 13.3, 13.3 Hz, 1 H), 2.33 (dd, *J* = 13.3, 13.3 Hz, 1 H), 2.24-2.13 (m, 3 H), 1.55-1.47 (m, 1 H), 1.26-1.22 (m, 1 H). ^{13}C NMR (125 MHz, C_6D_6): δ = 140.4, 138.4, 137.8, 129.2, 128.7, 128.6, 128.3, 127.9, 127.5, 121.6, 120.9, 119.6, 108.9, 101.7, 67.7, 48.2, 31.8 (2 C), 29.6, 24.8. MS (EI, 70 eV) *m/z*: 355 [M^+, 21], 248 [M^+, -$S_2C_3H_7$, 100], 231 [M^+, -$S_2C_3H_7$, -OH, 22].HRMS (EI): *m/z* [M^+] calcd for $C_{20}H_{21}NOS_2$: 355.1058; found: 355.1065. Anal. Calcd for $C_{20}H_{21}NOS_2$: C, 67.57; H, 5.95; N, 3.94; S, 18.04; found: C, 67.51; H, 6.03; N, 4.03; S, 17.99.

(2-(1,3-Dithian-2-yl)phenyl)(4-(diphenylamino)phenyl)methanol (5f): By following the general procedure **5f** was obtained as a yellow solid from dithioacetals **3** in 81% yield, 0.380 g; m.p.: 71-73 °C. ^{1}H NMR (500 MHz, C_6D_6): δ = 7.90 (d, *J* = 7.3 Hz, 1 H), 7.27 (d, *J* = 7.3 Hz, 1 H), 7.23 (d, *J* = 7.3 Hz, 2 H), 7.07-6.92 (m, 12 H), 6.77 (dd, *J* = 7.3, 7.3 Hz, 2 H), 6.04 (d, *J* = 3.2 Hz, 1 H), 5.51 (s, 1 H), 3.47-2.41 (m, 2 H), 2.29-2.23 (m, 2 H), 2.15 (d, *J* = 3.2 Hz, 1 H), 1.62-1.51 (m, 1 H), 1.36-1.30 (m, 1 H). ^{13}C NMR (125 MHz, C_6D_6): δ = 149.1, 148.1, 142.3, 138.9, 138.4, 130.4, 130.2 (3 C), 129.4 (2 C), 129.3, 129.2 (2 C), 129.0 (3 C), 125.2 (2 C), 125.1 (3 C), 123.6 (2 C), 73.9, 49.3, 33.1 (2 C), 26.0. MS (EI, 70 eV) *m/z*: 469 [M^+, 80], 362 [M^+, -$S(CH_2)_3SH$, 100], 346 [M^+, -$S(CH_2)_3SH$, -OH, 71]. HRMS (EI): *m/z* [M^+] calcd for $C_{29}H_{27}NOS_2$: 469.1534; found 469.1534. Anal. calcd for $C_{29}H_{27}NOS_2$: C, 74.16; H, 5.79; N, 2.98; S, 13.65. Found: C, 74.25; H, 5.82; N, 2.99; S, 13.38.

(2-(1,3-Dithian-2-yl)phenyl)(9-ethyl-9*H*-carbazol-3-yl)methanol (5g): By following the general procedure **5g** was obtained as a white foam from dithioacetals **3** in 88% yield, 0.369 g; m.p.: 79-81 °C. ^{1}H NMR (500 MHz, C_6D_6): δ = 8.35 (s, 1 H), 7.99 (d, *J*

= 7.5 Hz, 1 H), 7.93 (d, J = 7.5 Hz, 1 H), 7.52 (d, J= 7.5 Hz, 1 H), 7.42 (d, J= 7.5 Hz, 1 H), 7.32 (dd, J = 7.5, 7.5 Hz, 1 H), 7.15-7.12 (m, 1 H), 7.07-6.93 (m, 4 H), 6.49 (s, 1 H), 5.71 (s, 1 H), 3.60 (q, J = 7.2 Hz, 2 H), 2.46-2.36 (m, 3 H), 2.23-2.12 (m, 2 H), 1.51 (q, J = 12.7 Hz, 1 H), 1.26-1.15 (m, 1 H), 0.83 (t, J = 7.2 Hz, 3 H). ^{13}C NMR (125 MHz, C_6D_6): δ = 142.8, 141.4, 140.4, 138.6, 135.1, 130.2, 129.5, 129.4, 129.3, 126.6, 126.0, 124.2, 124.0, 121.7, 119.9 (2 C), 109.4, 109.3, 74.37, 49.5, 49.4, 38.0, 33.1 (2 C), 26.0, 14.2. MS (EI, 70 eV) *m/z*:419 [M^+, 6]; 313 [M^+, -$S(CH_2)_3S$, 100]; 296 [M+, -$S(CH_2)_3S$, -OH, 67]. HRMS (EI): *m/z* [M^+] calcd for $C_{25}H_{25}NOS_2$: 419.1378; found: 419.1383. Anal. calcd for $C_{25}H_{25}NOS_2$: C, 71.56; H, 6.01; N, 3.34; S, 15.28; found: C, 71.59; H, 6.08; N, 3.31; S, 15.34.

(2-(1,3-Dithian-2-yl)phenyl)(4-methoxyphenyl)methanol (5h): By following the general procedure **5h** was obtained as a white solid from dithioacetals **3** in 79% yield, 0.262 g. ^{1}H NMR (500 MHz, C_6D_6): δ = 7.92 (dd, J = 7.6, 1.1 Hz, 1 H), 7.30 (d, J =8.6 Hz, 2 H), 7.27 (dd, J = 7.6, 1.1 Hz, 1 H), 7.03-6.93 (m, 2 H), 6.78-6.66 (m, 2 H), 5.52 (s, 1 H), 6.16 (s, 1 H), 3.21 (s, 3 H), 2.53-2.31 (m, 3 H), 2.30-2.07 (m, 2 H), 1.64-1.44 (m, 1 H), 1.35-1.22 (m, 1 H). ^{13}C NMR (125 MHz, C_6D_6): δ = 159.1, 141.2, 137.4, 135.4, 129.1, 128.4, 128.33, 128.31, 128.1, 128.0, 113.7 (2 C), 72.5, 54.4, 48.3, 32.08, 32.04, 25.0. Anal. calcd for $C_{18}H_{20}O_2S_2$: C, 65.02; H, 6.06; S, 19.29; found: C, 64.87; H, 6.15; S, 19.21.

(6-(1,3)-Dithian-2-yl-benzo[*d*][1,3]dioxol-5-yl)(benzo[*d*][1,3]dioxol-5-yl)methanol (6a): By following the general procedure **6a** was obtained as a white foam from dithioacetals **4** in 76% yield; 0.296 g; m.p.: 149-151 °C. ^{1}H NMR (500 MHz, C_6D_6): δ = 7.55 (s, 1 H), 7.08 (s, 1 H), 6.89 (d, J = 8.0 Hz, 1 H), 6.86 (s, 1 H), 6.66 (d, J = 8.0 Hz, 1 H), 6.06 (s, 1 H), 5.50 (s, 1 H), 5.27 (s, 2 H), 5.20 (d, J = 1.1 Hz, 1 H), 5.16 (d, J = 1.1 Hz, 1 H), 2.46 (m, 2 H), 2.32-2.18 (m, 2 H), 2.12 (s, 1 H), 1.62-1.44 (m, 1 H),

1.39–1.27 (m, 1 H), ^{13}C NMR (125 MHz, C_6D_6): δ = 149.0, 148.7, 148.0, 148.9, 138.5, 136.5, 131.8, 121.0, 110.0, 109.3, 108.9, 108.4, 102.00, 101.6, 73.0, 49.0, 33.0, 32.9, 25.8. MS (EI, 70 eV): *m/z*: 390 [M^+, 2], 284 [M^+, -$S_2C_3H_6$, 100], 283 [M^+, -$HS_2C_3H_6$, 96], 267 [M^+, - $S_2C_3H_6$, -OH, 27], 254 [M^+, -$S_2C_4H_7$, -OH, 18]. HRMS (EI): *m/z* [M^+] calcd for $C_{19}H_{18}O_5S_2$: 390.0596; found: 390.0602. Anal. calcd for $C_{19}H_{18}O_5S_2$: C, 58.44; H, 4.65; S, 16.42; found: C, 58.44; H, 4.88; S, 16.66.

(6-(1,3)-Dithian-2-yl-benzo[*d*][1,3]dioxol-5-yl)(3,4,5-trimethoxyphenyl)methanol (6b): By following the general procedure **6b** was obtained as a white foam from dithioacetals **4** in 72% yield**,** 0.314 g; m.p.: 214–216 °C. ^1H NMR (200 MHz, $CDCl_3$): δ = 7.12 (s, 1 H), 6.65 (s, 1 H), 6.61 (s, 2H), 6.11 (s, 1 H), 5.92 (s, 2 H), 5.44 (s, 1 H), 3.81 (s, 3 H), 3.80 (s, 6 H), 3.08–2.77 (m, 4 H), 2.68 (s, 1 H), 2.18–1.74 (m, 2 H). ^{13}C NMR (50 MHz, $CDCl_3$): δ = 151.9 (2 C), 146.5, 146.3, 143.7, 137.1, 133.7, 129.3, 107.5, 107.2, 102.0 (2 C), 100.2, 70.7, 59.6, 54.9 (2 C), 46.8, 31.2 (2 C), 23.7.MS (EI, 70 eV): *m/z* (%): 436 [M^+, 15], 329 [M^+, -$HS_2C_3H_6$, 96], 328 [M^+, -$H_2S_2C_3H_6$, 100], 299 [M^+, -$S_2C_4H_7$, -H_2O, 79]. HRMS (EI): *m/z* [M^+] calcd for $C_{21}H_{24}O_6S_2$: 436.1014; found: 436.1023. Anal. calcd for $C_{21}H_{24}O_6S_2$: C, 57.78; H, 5.54; S, 14.69; found: C, 57.68; H, 5.38; S, 14.42.

General procedure for preparation of *ortho*-1,3-dithianylaryl(aryl)methanes 7a–h and 8a-b

To a solution of the corresponding diarylmethanol (**5a-h**, **6a,b**) (1 mmol) in 1,2-dichloroethane or benzene (30 mL, Table 1) were added solid zinc iodide (1.5 mmol) and sodium cyanoborohydride (7 mmol). The mixture was stirred at room temperature or at reflux (due to a weak solubility) for 2–24 h. Then, the mixture was

filtered through the Celite® pad and eluted with dichloroethane (100 mL). The filtrate was washed with saturated ammonium chloride (20 mL), water (20 mL) and dried over anhydrous $MgSO_4$. After removal of the solvent under reduced pressure, the residue was purified with silica gel column chromatography using petroleum ether as eluent to give the desired diarylmethanes (**7a–h**, **8a–b**).

5-(1,3-Dithian-2-yl)benzyl)benzo[*d*][1,3]dioxole (7a): By following the general procedure **7a** was obtained as a yellow solid from diarylmethanol **5a** in 95% yield, 0.313 g; m.p.: 125-127 °C. ^{1}H NMR (500 MHz, C_6D_6): δ = 7.94 (d, *J* = 7.4 Hz, 1 H), 6.99 (dd, *J* =7.4, 7.4 Hz, 1 H), 6.90 (dd, *J* =7.4, 7.4 Hz, 1 H), 6.87 (d, *J* = 7.4 Hz, 1 H), 6.64 (s, 1 H), 6.57 (d, *J* = 7.9 Hz, 1 H), 6.47 (d, *J* = 7.9 Hz, 1 H), 5.34 (s, 1 H), 5.22 (s, 2 H), 3.93 (s, 2 H), 2.49-2.37 (m, 2 H), 2.28-2.21 (d, 2 H), 1.63-1.50 (m, 1 H), 1.38-1.25 (m, 1 H). ^{13}C NMR (125 MHz, C_6D_6): δ = 148.1, 146.2, 137.9, 137.8, 134.2, 130.5, 128.9, 128.3, 127.3, 121.7, 109.3, 108.1, 100.5, 48.5, 38.2, 32.1 (2 C), 25.0.MS (EI, 70 eV) *m/z* (%): 330 [M^+, 5], 255 [M^+, -HSC_3H_6, 70], 223 [M^+, -$S_2C_3H_7$, 59], 222 [M^+, -$HS_2C_3H_7$, 100], 165 [M^+, -$S_2C_3H_7$, -O_2CH_2, 34]. HRMS (EI): *m/z* [M^+] calcd for $C_{18}H_{18}O_2S_2$: 330.0748; found: 330.0747. Anal. Calcd for $C_{18}H_{18}O_2S_2$: C, 65.42; H, 5.49; S, 19.41; found: C, 65.26; H, 10.66, S, 19.48.

2-(2-(3,4,5-Trimethoxybenzyl)phenyl)-1,3-dithiane (7b): By following the general procedure **7b** was obtained as a yellow oil from diarylmethanol **5b** in 95% yield, 0.350 g. ^{1}H NMR (500 MHz, C_6D_6): δ = 8.00 (d, *J* = 7.6 Hz, 1 H), 7.07-6.89 (m, 3 H), 6.35 (s, 2 H), 5.41 (s, 1 H), 4.08 (s, 2 H), 3.80 (s, 3 H), 3.35 (s, 6 H), 2.47-2.39 (m, 2 H), 2.25 (dd, *J* = 4.0, 3.2 Hz, 1 H), 2.28 (dd, *J* = 4.0, 3.2 Hz, 1 H), 1.64-1.53 (m, 1 H), 1.36-1.28 (m, 1 H). ^{13}C NMR (125 MHz, C_6D_6): δ = 154.0 (2 C), 138.0, 137.5 (2 C), 135.4, 130.4, 128.9 (2 C), 128.4, 106.2 (2 C), 60.1, 55.4 (2 C), 48.6, 38.7, 31.9 (2 C), 24.9. MS (EI, 70 eV) *m/z*: 376 [M^+, 23], 301 [M^+, -HSC_3H_6, 35], 269 [M^+, -$HS_2C_3H_6$,

41], 268 [M^+, -$H_2S_2C_3H_6$, 100]. HRMS (EI): *m/z* [M^+] calcd for $C_{20}H_{24}O_3S_2$: 376.1167; found: 376.1161. Anal. calcd for $C_{20}H_{24}O_3S_2$: C, 63.80; H, 6.42; S, 17.03; found: C, 64.09; H, 6.54; S, 16.91.

2-(2-[1,3]Dithian-2-yl-benzyl)benzo[*b*]thiophene (7c): By following the general procedure **7c** was obtained as a yellow solid from diarylmethanol **5c** in 70% yield, 0.239 g; m.p.: 115-116 °C. ^{1}H NMR (500 MHz, C_6D_6): δ = 7.95 (d, *J* = 7.9 Hz, 1 H), 7.44 (d, *J* = 7.9 Hz, 1 H), 7.37 (d, *J* = 7.9 Hz, 1 H), 7.06-6.98 (m, 2 H), 6.97-6.92 (m, 3 H), 6.75 (s, 1 H), 5.38 (s, 1 H), 4.20 (s, 2 H), 2.42-2.35 (m, 2 H), 2.21 (dd, *J* = 3.3, 3.3 Hz, 1 H), 2.19 (dd, *J* = 3.3, 3.3 Hz, 1 H), 1.60-1.44 (m, 1 H), 1.32-1.20 (m, 1 H). ^{13}C NMR (125 MHz, C_6D_6, 25 ^{o}C): δ = 145.3, 141.3, 140.9, 139.0, 137.4, 131.5, 130.0, 129.4, 129.0, 125.2, 124.8, 124.1, 123.1, 123.1, 49.8, 34.8, 33.0 (2 C), 26.0. MS (EI, 70 eV) *m/z* (%): 342 [M^+, 4], 267 [M^+, -HSC_3H_6, 11], 234 [M^+, -$S_2C_4H_6$, 27], 235 [M^+, -$S_2C_4H_7$, 78], 234 [M^+, -$HS_2C_4H_7$, 100]. HRMS (EI): *m/z* [M^+] calcd for $C_{19}H_{18}S_3$: 342.0571; found: 342.0567. Anal. calcd for $C_{19}H_{18}S_3$: C, 66.62; H, 5.30; S, 28.08. Found: C, 66.43; H, 5.21; S, 28.14.

2-(2-(Thien-2-ylmethyl)phenyl)-1,3-dithiane (7d): By following the general procedure **7d** was obtained as a yellow solid from diarylmethanol **5d** in 95% yield, 0.277 g; m.p.: 53-55 °C. ^{1}H NMR (500 MHz, C_6D_6): δ = 8.02 (d, *J* = 7.3 Hz, 1 H), 7.08 (dd, *J* = 7.3, 7.3 Hz, 1 H), 7.04-6.96 (m, 2 H), 6.85 (d, *J* = 4.9 Hz, 1 H), 6.74 (dd, *J* = 4.9, 3.6 Hz, 1 H), 6.72-6.68 (m, 1 H), 5.47 (s, 1 H), 4.26 (s, 2 H), 2.59-2.46 (m, 2 H), 2.36 (dd, *J* = 3.3, 3.3 Hz, 1 H), 2.34 (dd, *J* = 3.3, 3.3 Hz, 1H), 1.74-1.58 (m, 1 H), 1.47-1.33 (m, 1 H). ^{13}C NMR (125 MHz, C_6D_6): δ = 143.3, 137.8, 137.3, 130.3, 129.0, 128.5, 127.0, 125.5, 123.9 (2 C), 48.7, 33.1, 32.1 (2 C), 25.1. MS (EI, 70 eV) *m/z* (%): 292 [M^+, 3], 217 [M^+, -HSC_3H_6, 50], 185 [M^+, -$HS_2C_3H_6$, 73], 184 [M^+, -$H_2S_2C_3H_6$,

100]. HRMS (EI): *m/z* [M^+] calcd for $C_{15}H_{16}S_3$: 292.0414; found: 292.0404. Anal. calcd for $C_{15}H_{16}S_3$: C, 61.60; H, 5.51; S, 32.89; found: C, 61.50; H, 5.53; S, 32.68.

2-(2-[1,3]Dithian-2-yl-benzyl)-1-methyl-1*H*-indole (7e): By following the general procedure **7e** was obtained as a white solid from diarylmethanol **5e** in 26% yield, 0.088 g; m.p.: 165-167 °C. ^{1}H NMR (500 MHz, C_6D_6): δ = 7.95 (d, *J* = 7.6 Hz, 1 H), 7.68-7.55 (m, 1 H), 7.22-7.13 (m, 2 H), 6.99 (dd, *J* = 7.6, 7.6 Hz, 2 H), 6.86 (dd, *J* = 7.6, 7.6 Hz, 1 H), 6.76 (d, *J* = 7.6 Hz, 1 H), 6.28 (s, 1 H), 5.36 (s, 1 H), 4.03 (s, 2 H), 2.95 (s, 3 H), 2.50-2.40 (m, 2 H), 2.29 (dd, *J* = 3.3, 3.3 Hz, 1 H), 2.26 (dd, *J* = 3.3, 3.3 Hz, 1 H), 1.66-1.51 (m, 1 H), 1.40-1.27 (m, 1 H). ^{13}C NMR (125 MHz, C_6D_6): δ 139.1, 138.7, 138.5, 136.7, 130.4, 129.6, 129.5, 129.3, 129.0, 122.0, 121.3, 120.6, 109.9, 102.9, 49.4, 32.9 (2 C), 31.1, 30.0, 26.0. MS (EI, 70 eV) *m/z*: 339 [M^+, 22], 232 [M^+, -$HS_2C_3H_6$, 100], 217 [M^+,-$HS_2C_3H_6$, -CH_3, 28]. HRMS (EI): *m/z* [M^+] calcd for $C_{20}H_{21}NS_2$: 339.1115; found: 339.1107. Anal. Calcd for $C_{20}H_{21}NS_2$: C, 70.75; H, 6.23; N, 4.13; S, 18.89; found: C, 70.87; H, 6.07; N, 4.23; S, 19.01.

2-(4-(Diphenylamino)phenylmethyl)phenyl)-1,3-dithiane (7f): By following the general procedure **7f** was obtained as a white solid from diarylmethanol **5f** in 95% yield, 0.430 g; m.p.: 57-59 °C. ^{1}H NMR (500 MHz, C_6D_6): δ = 8.00 (d, *J* = 7.8 Hz, 1 H), 7.11-6.96 (m, 15 H), 6.85-6.79 (m, 2 H), 5.43 (s, 1 H), 4.04 (s, 2 H), 2.53-2.45 (m, 2 H), 2.38-2.30 (m, 2 H), 1.74-1.54 (m, 1 H), 1.47-1.32 (m, 1 H). ^{13}C NMR (125 MHz, C_6D_6): δ = 148.1 (2 C), 146.2, 138.0, 137.9, 135.2, 130.6, 129.8 (2 C), 129.2 (4 C), 129.0, 128.3, 127.3, 124.8 (2 C), 124.0 (4 C), 122.4 (2 C), 48.6, 38.1, 32.1 (2 C), 25.1. MS (EI, 70 eV) *m/z*: 453 [M^+, 87], 346 [M^+, -$HS_2C_3H_6$, 100]. HRMS (EI): *m/z* [M^+] calcd for $C_{29}H_{27}NS_2$: 453.1585; found: 453.1583. Anal. calcd for $C_{29}H_{27}NS_2$: C, 76.78; H, 6.00; N, 3.09; S, 14.14; found: C, 76.82; H, 5.94; N, 3.11; S, 14.17.

3-(2-[1,3]Dithian-2-yl-benzyl)-9-ethyl-9*H*-carbazole (7g): By following the general procedure **7g** was obtained as a white solid from diarylmethanol **5g** in 59% yield; 0.238 g; m.p.: 140-142 ^{o}C. ^{1}H NMR (500 MHz, C_6D_6): δ = 8.09-7.98 (m, 1 H), 7.98-7.87 (m, 2 H), 7.33 (ddd, *J* = 8.3, 7.3, 1.1 Hz, 1H), 7.27 (dd, *J* = 8.3, 1.6 Hz, 1 H), 7.08-6.92 (m, 5 H), 7.18-7.12 (m, 1 H), 5.57 (s, 1 H), 4.36 (s, 2 H), 3.62 (q, *J* = 7.2 Hz, 2 H), 2.49-2.39 (m, 2 H), 2.26 (dd, *J* = 3.2, 3.2 Hz, 1 H), 2.24 (dd, *J* = 3.2, 3.2 Hz, 1 H), 1.57 (dtt, *J* = 15.5, 12.6, 3.2 Hz, 1 H), 1.33-1.25 (m, 1 H), 0.84 (t, *J* = 7.2 Hz, 3 H). ^{13}C NMR (125 MHz, C_6D_6): δ = 141.3, 139.8 (2 C), 139.0, 131.6 (2 C), 129.9, 129.3, 128.2, 127.7, 126.5, 124.4, 124.1, 121.6, 121.6, 119.8, 109.5, 109.4, 49.7, 39.6, 38.0, 33.1 (2 C), 26.1, 14.2. MS (EI, 70 eV) *m/z* (%): 403 [M^+, 38]; 396 [M^+, -$S(CH_2)_2CH_3$, 100]. HRMS (EI): *m/z* [M^+] calcd for $C_{25}H_{25}NS_2$: 403.1428; found: 403.1418. Anal. calcd for $C_{25}H_{25}NS_2$: C, 74.40; H, 6.24; N, 3.47; S, 15.89; found: C, 74.36; H, 6.20; N, 3.51; S, 16.02.

2-(2-(4-Methoxybenzyl)phenyl)-1,3-dithiane (7h): By following the general procedure **7h** was obtained as a white solid from diarylmethanol **5h** in 64% yield, 0.202 g; m.p.: 115-117 ^{o}C. ^{1}H NMR (500 MHz, C_6D_6): δ = 1.38-1.26 (m, 1 H), 1.66-1.47 (m, 1 H), 2.25-2.22 (m, 1 H), 2.30-2.25 (m, 1 H), 2.50-2.35 (m, 2 H), 3.22 (s, 3 H), 4.03 (s, 2 H), 5.38 (s, 1 H), 6.74-6.68 (m, 2 H), 7.04-6.88 (m, 5 H), 7.97 (d, *J* =7.5 Hz, 1 H). ^{13}C NMR (125 MHz, C_6D_6): δ = 158.4, 138.1, 138.0, 132.2, 130.5, 129.7, 128.8, 128.3, 128.0, 127.2, 114.0 (2 C), 54.4, 48.6, 37.7, 32.1 (2 C), 25.1. MS (EI, 70 eV) *m/z*: 316 [M^+, 10], 241 [M^+, -HSC_3H_6, 100]. Anal. calcd for $C_{18}H_{20}OS_2$: C, 68.31; H, 6.37; S, 20.26; found: C, 68.12; H, 6.26; S, 16.99.

5-(Benzo[*d*][1,3]dioxol-5-ylmethyl)-6-(1,3-dithian-2-yl)benzo[*d*][1,3]dioxole (8a): By following the general procedure **8a** was obtained as a white solid from diarylmethanol **6a** in 95% yield, 0.355 g; m.p.: >250 °C. ^{1}H NMR (500 MHz, C_6D_6): δ

= 7.62 (s, 1 H), 6.72 (s, 1 H), 6.64 (d, *J* =8.0 Hz, 1 H), 6.54 (dd, *J* = 8.0, 8.0 Hz, 1 H), 6.48 (s, 1 H), 5.35 (s, 1 H), 5.27 (s, 2 H), 5.20 (s, 2 H), 3.85 (s, 2 H), 2.50-2.42 (m, 2 H), 2.31-2.24 (m, 2 H), 1.63-1.50 (m, 1 H), 1.38-1.30 (m, 1 H). ^{13}C NMR (125 MHz, C_6D_6): δ = 149.1, 148.8, 148.0, 147.3, 135.4, 132.6, 132.0, 122.5, 111.4, 110.2, 110.0, 109.1, 101.8, 101.5, 49.3, 39.0, 33.0 (2 C), 25.9. MS (EI, 70 eV) *m/z*: 374 [M^+, 9], 299 [M^+, -HSC_3H_6, 100], 267 [M^+, -$HS_2C_3H_6$, 86], 266 [M^+, -$H_2S_2C_3H_6$, 80]. HRMS (EI): *m/z* [M^+] calcd for $C_{19}H_{18}O_4S_2$: 374.0646; found: 374.0649. Anal. Calcd for $C_{19}H_{18}O_4S_2$: C, 60.94; H, 4.84; S, 17.13; found: C, 60.23; H, 4.58; S, 16.99.

5-(1,3-Dithian-2-yl)-6-(3,4,5-trimethoxybenzyl)benzo[*d*][1,3]dioxole (8b): By following the general procedure **8b** was obtained as a white solid from diarylmethanol **6b** in 60% yield, 0.252 g; m.p.: 146-148 °C. ^{1}H NMR (500 MHz, C_6D_6): δ = 7.67 (s, 1 H), 6.62 (s, 1 H), 6.44 (s, 2 H), 5.42 (s, 1 H), 5.20 (s, 2 H), 3.99 (s, 2 H), 3.85 (s, 3 H), 3.44 (s, 6 H), 2.50-2.40 (m, 2 H), 2.35-2.21 (m, 2 H), 1.65-1.53 (m, 1 H), 1.44-1.28 (m, 1 H). ^{13}C NMR (125 MHz, C_6D_6): δ = 155.0 (2 C), 149.0, 148.1, 138.6, 136.6, 132.4, 132.2, 111.3, 110.0, 107.1 (2 C), 101.9, 61.2, 56.6 (2 C), 49.4, 39.6, 32.9 (2 C), 25.9. MS (EI, 70 eV) *m/z*: 420 [M^+, 15], 345 [M^+, -HSC_3H_6, 55], 313 [M^+, -$HS_2C_3H_6$, 68], 312 [M^+, -$H_2S_2C_3H_6$, 100]. HRMS (EI): *m/z* [M^+] calcd for $C_{21}H_{24}O_5S_2$: 420.1065; found: 420.1076. Anal. calcd for $C_{21}H_{24}O_5S_2$: C, 59.98; H, 5.75; S, 15.25; found: C, 59.71; H, 5.54; S, 15.13.

5-(3,4,5-Trimethoxyphenyl)-5,7-dihydro-furo[3',4':4,5]benzo[1,2-d][1,3]dioxole (10): To a stirred solution of *ortho*-1,3-dioxanyl-diarylmethanol **9** (200 mg, 0.49 mmol) dissolved in EtOAc (5 mL), (10%) Pd/C (30 mg) was added. The mixture was stirred for 0.5 h at room temperature under hydrogen gas atmosphere (balloon). Then, the balloon with hydrogen gas was removed and the mixture was left for 18 h at rt. The crude mixture was filtered through the Celite® pad and concentrated in vacuo.

Preparative chromatography gave 330 mg (90%) of the product **10**. m.p.: 95 - 100 °C.[4,5] ^{1}H NMR (200 MHz, C_6D_6): δ = 3.40 (s, 6H), 3.91 (s, 3H), 4.98 (dd$_{AB}$, *J* = 2.3, 11.8, 1H), 5.15 (dd$_{AB}$, *J* = 2.3, 11.8, 1H), 5.35 (d, *J* = 1.0, 1H), 5.40 (d, *J* = 1.0, 1H), 6.07 (dd, *J* = 2.8, 2.8, 1H), 6.46 (s, 1H), 6.58 (s, 1H), 8.66 (s, 2H). ^{1}H NMR (500 MHz, CD_2Cl_2): δ = 3.76 (s, 3H), 3.81 (s, 6H), 5.06 (dd, *J* = 2.1, 11.7, 1H), 5.21 (dd, *J* = 3.0, 11.7, 1H), 5.95 (d, *J* = 1.1, 1H), 5.96 (d, *J* = 1.1, 1H), 5.96 (bs, 1H), 6.47 (s, 1H), 6.54 (s, 2H), 6.72 (s, 1H). ^{13}C NMR (137.5 MHz, CD_2Cl_2): δ = 56.5, 60.9, 73.7, 86.8, 101.9, 102.3, 103.0, 104.2, 132.4, 135.5, 138.3, 138.1, 148.2, 148.5, 154.0. Anal. Calcd for $C_{18}H_{18}O_6$: C, 65.45; H, 5.49. Found: C, 65.01; H, 5.79.

Literature

[1] Lai, J.; Du, W.; Tian, L.; Zhao, Ch.; She, X.; Tang, Sh. *Org. Lett.* **2014**, *16*, 4396−4399.

[2] Jung, M. E.; Lam, P. Y.-S.; Mansuri, M. M.; Speltz, L. M. *J.Org. Chem.* **1985**, *50*, 1087-1105.

[3] Takano, S.; Otaki, S.; Ogasawara, K. *Tetrahedron Lett.* **1985**, *26*, 1659-1660.

4] Arnold, B. J.; Mellows, S. M.; Sammes, P. G. *J. Chem. Soc., Perkin Trans. 1* **1973**, 1266–1270.

[5] Galletti, G. C.; Ward, R. S.; Pelter, A.; Goubet, D. *J. Anal. Appl. Pyrolysis* **1992**, *24*, 139–146.

2. Copies of ^{1}H and ^{13}C NMR spectra

^{1}H NMR spectrum of 2-(2-bromophenyl)-1,3-dithiane (3) (200 MHz, $CDCl_3$).

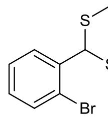

3

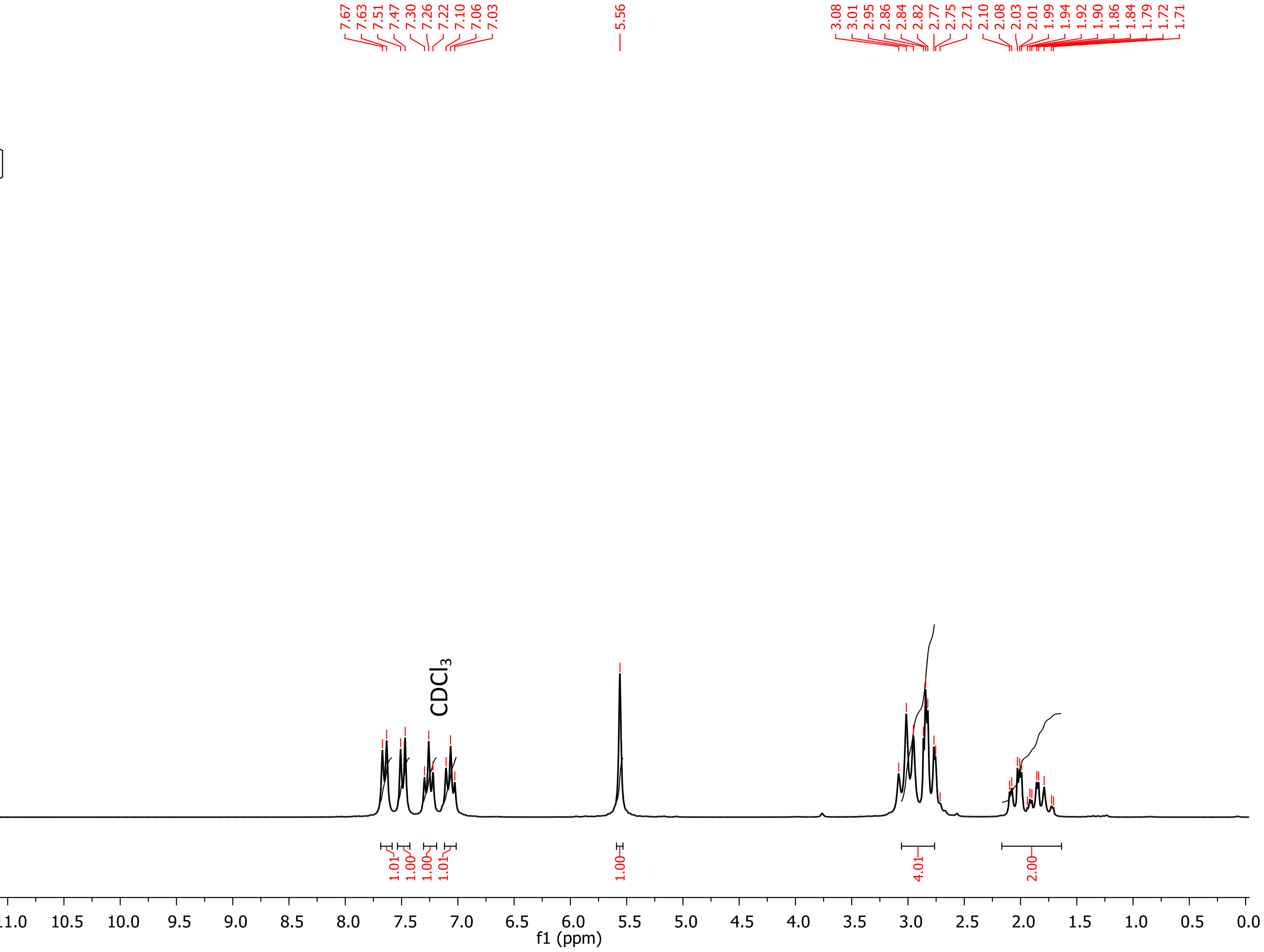

^{13}C NMR spectrum of 2-(2-bromophenyl)-1,3-dithiane (3) (50 MHz, $CDCl_3$).

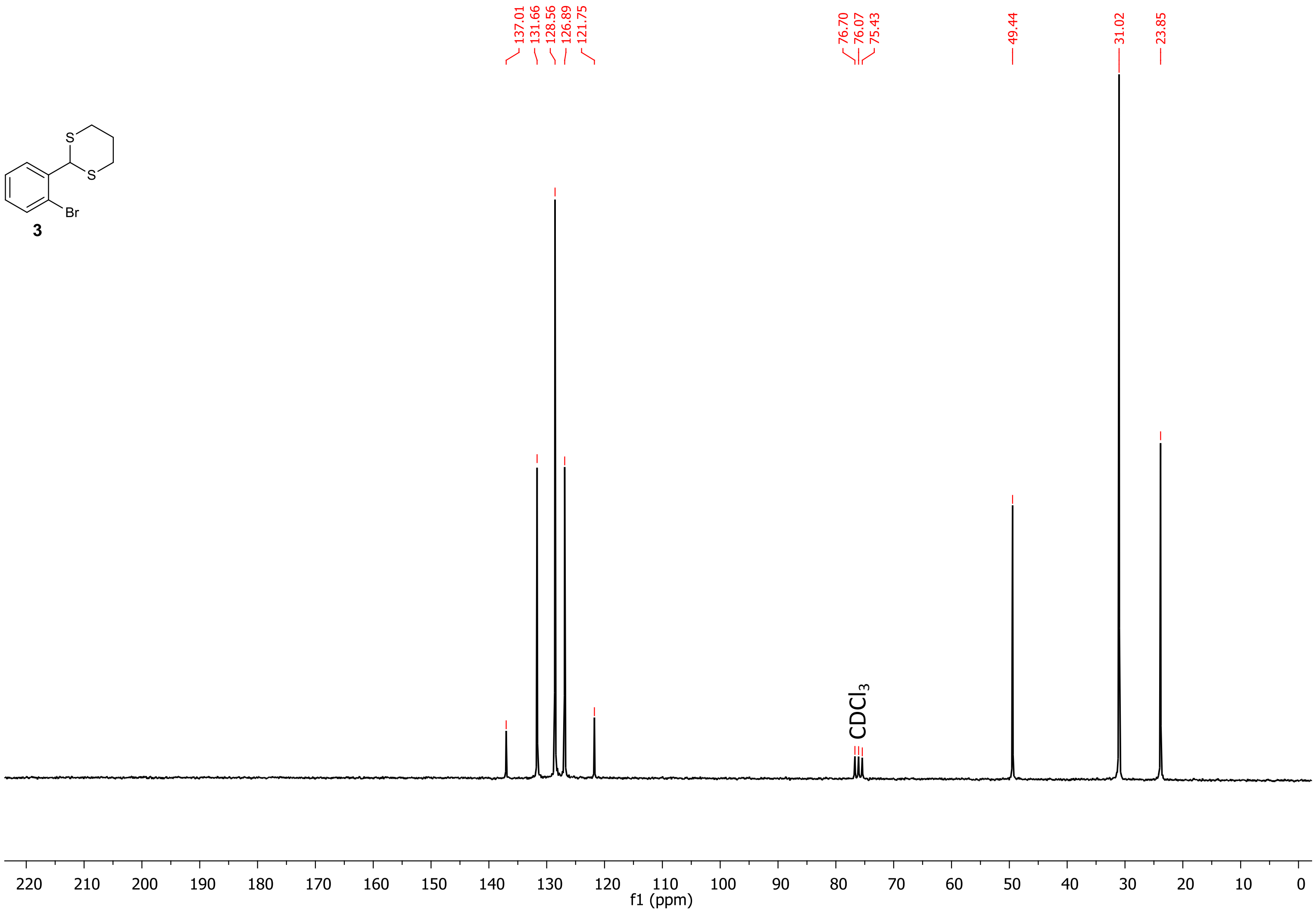

^{1}H NMR spectrum of 5-bromo-6-(1,3-dithian-2-yl)benzo-1,3-dioxole (4) (200 MHz, $CDCl_3$).

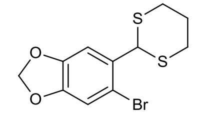

4

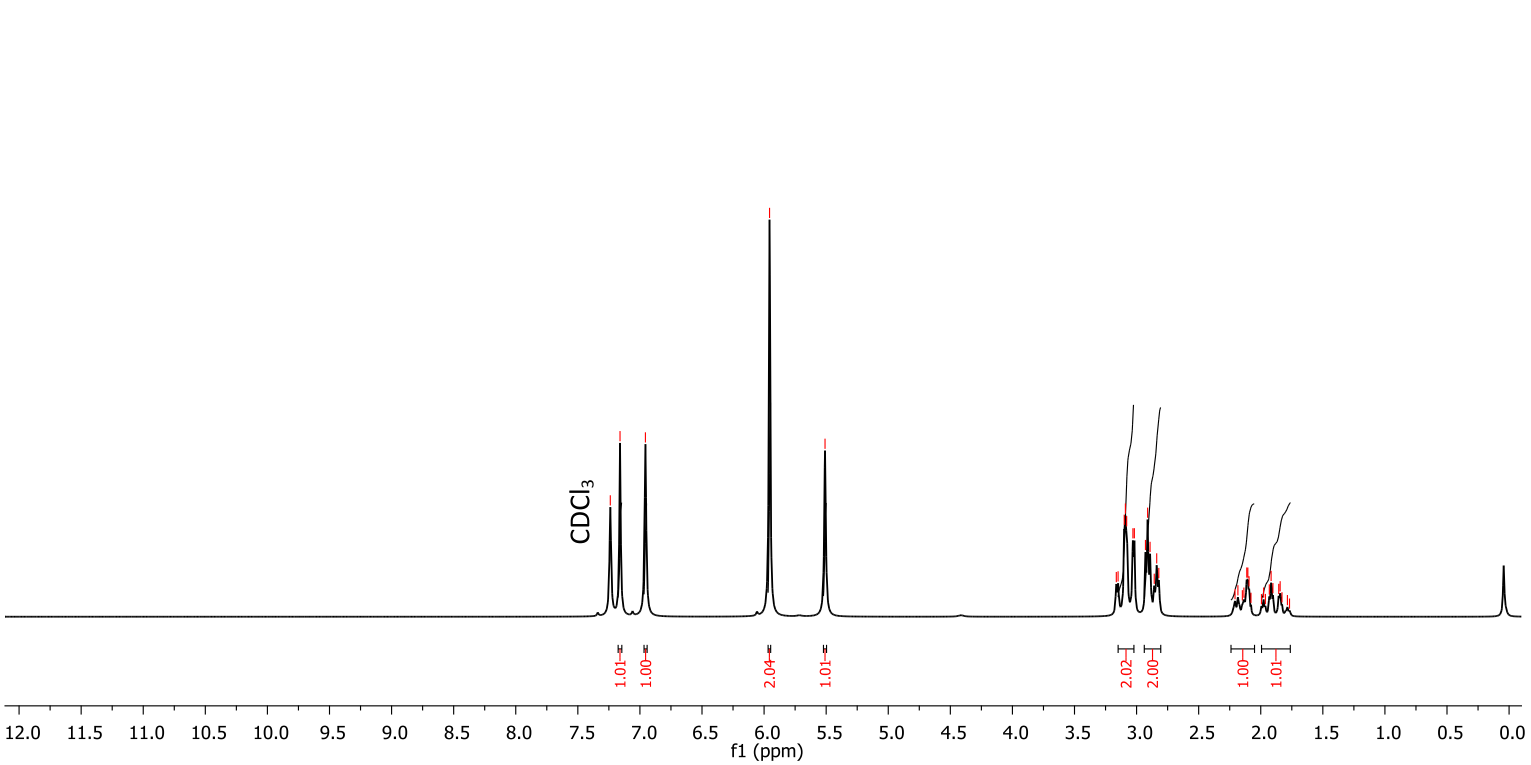

^{13}C NMR spectrum of 5-bromo-6-(1,3-dithian-2-yl)benzo-1,3-dioxole (3) (50 MHz, $CDCl_3$).

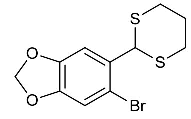

4

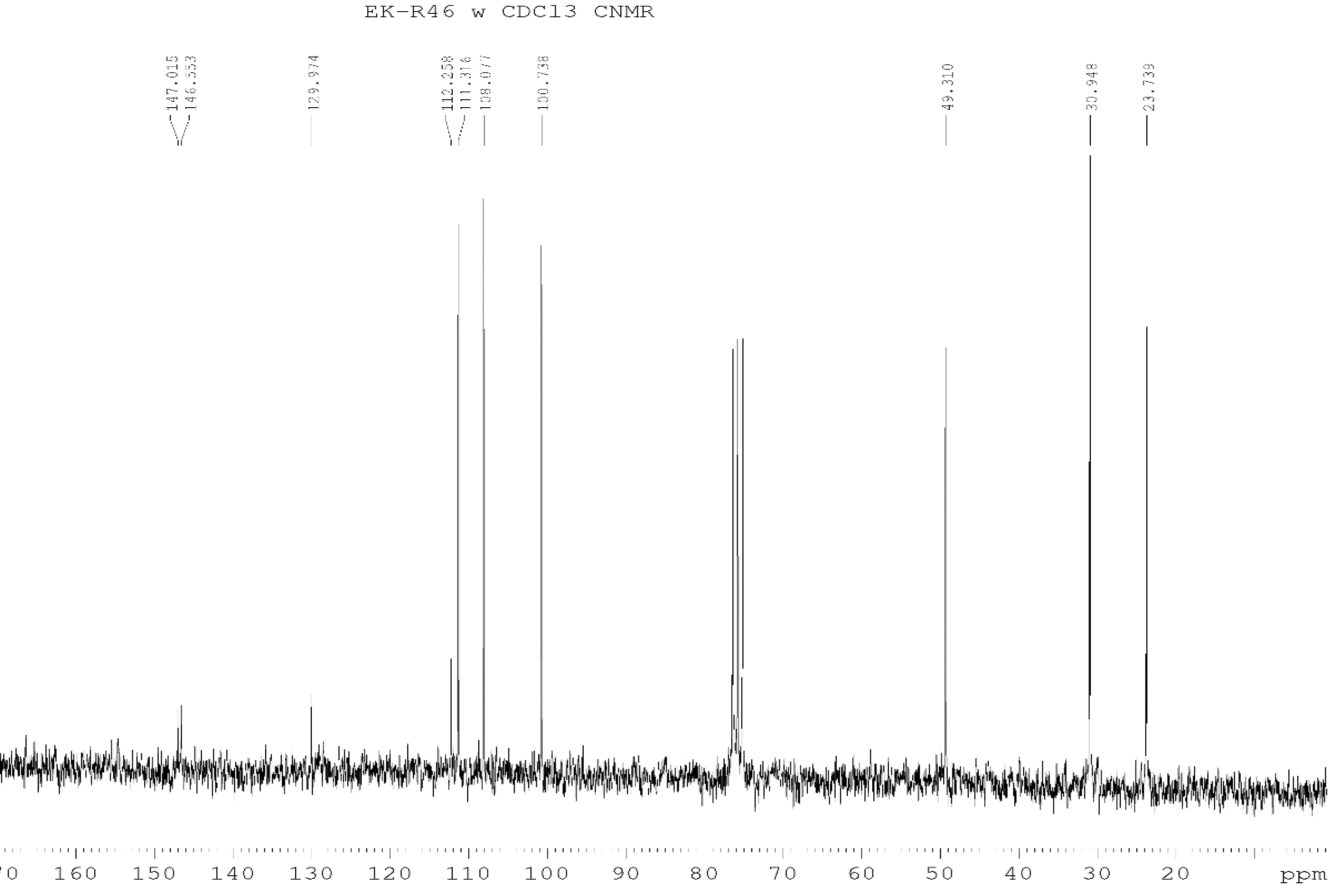

^{1}H NMR spectrum of (2-(1,3-dithian-2-yl)phenyl)(benzo[*d*][1,3]dioxol-5-yl)methanol (5a) (500 MHz, C_6D_6).

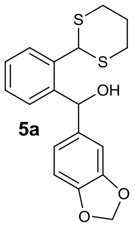

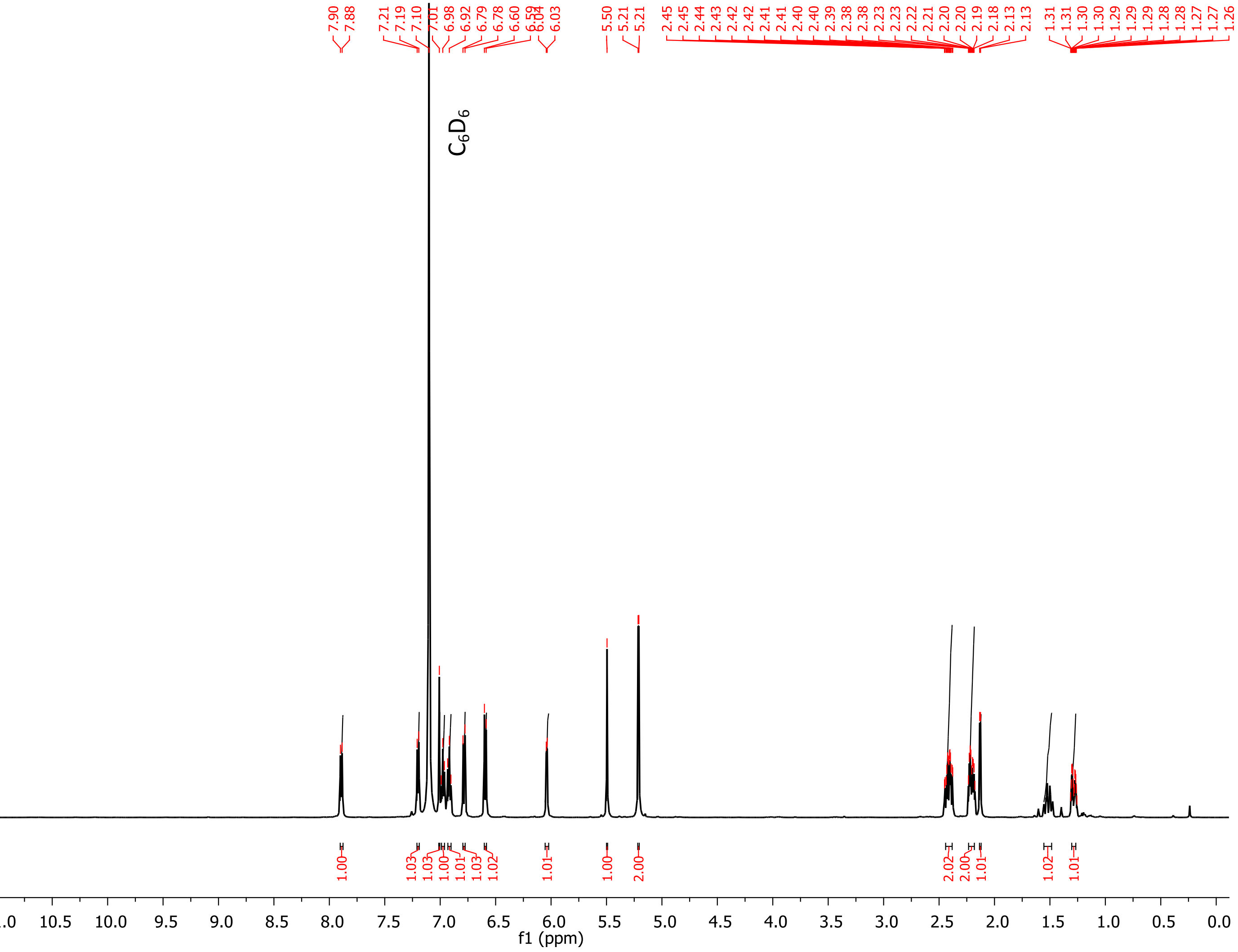

^{13}C NMR spectrum of (2-(1,3-dithian-2-yl)phenyl)(benzo[*d*][1,3]dioxol-5-yl)methanol (5a) (125 MHz, C_6D_6).

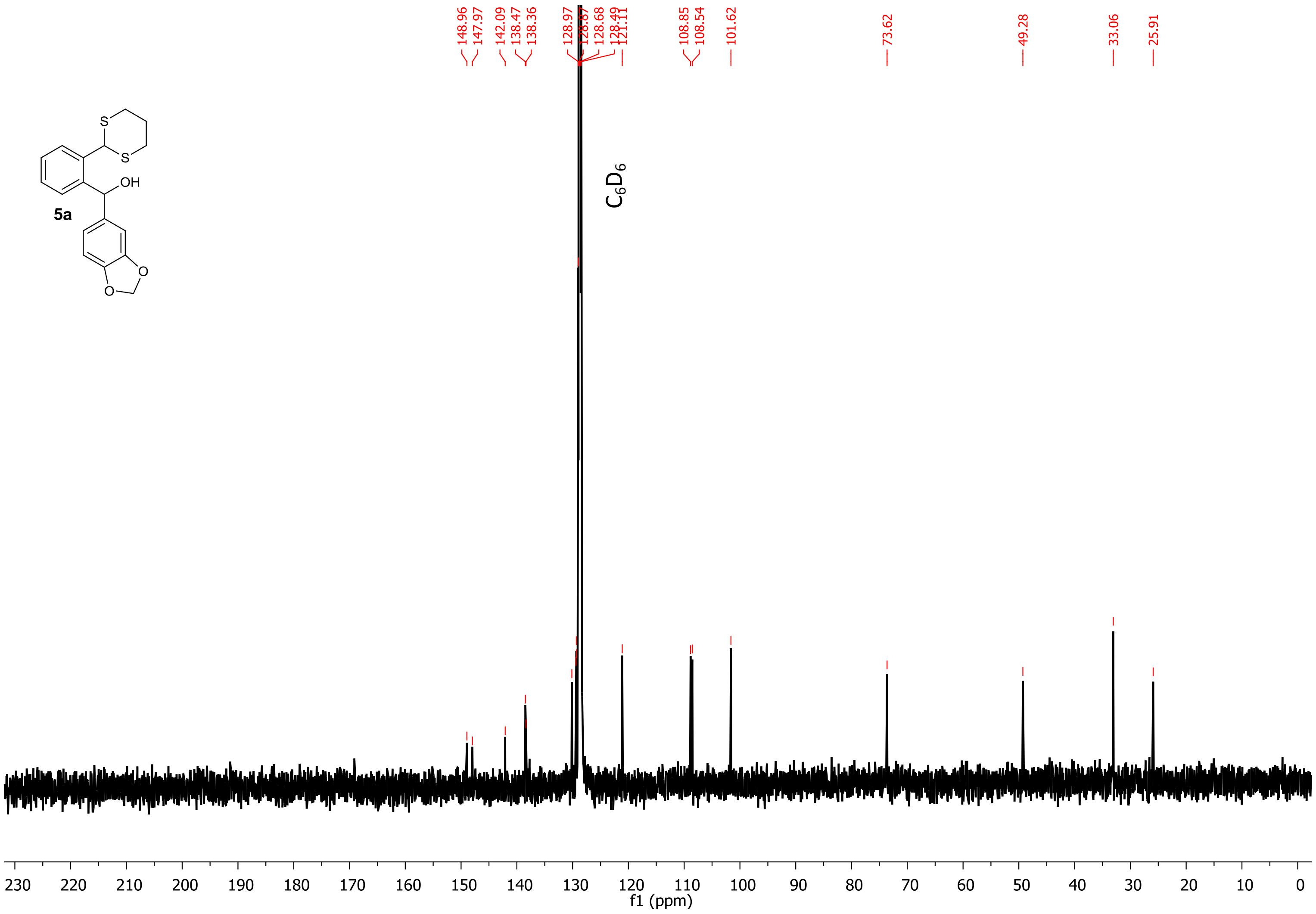

^{1}H NMR spectrum of 2-(1,3-dithian-2-yl)phenyl(3,4,5-trimethoxyphenyl)methanol (5b) (500 MHz, C_6D_6).

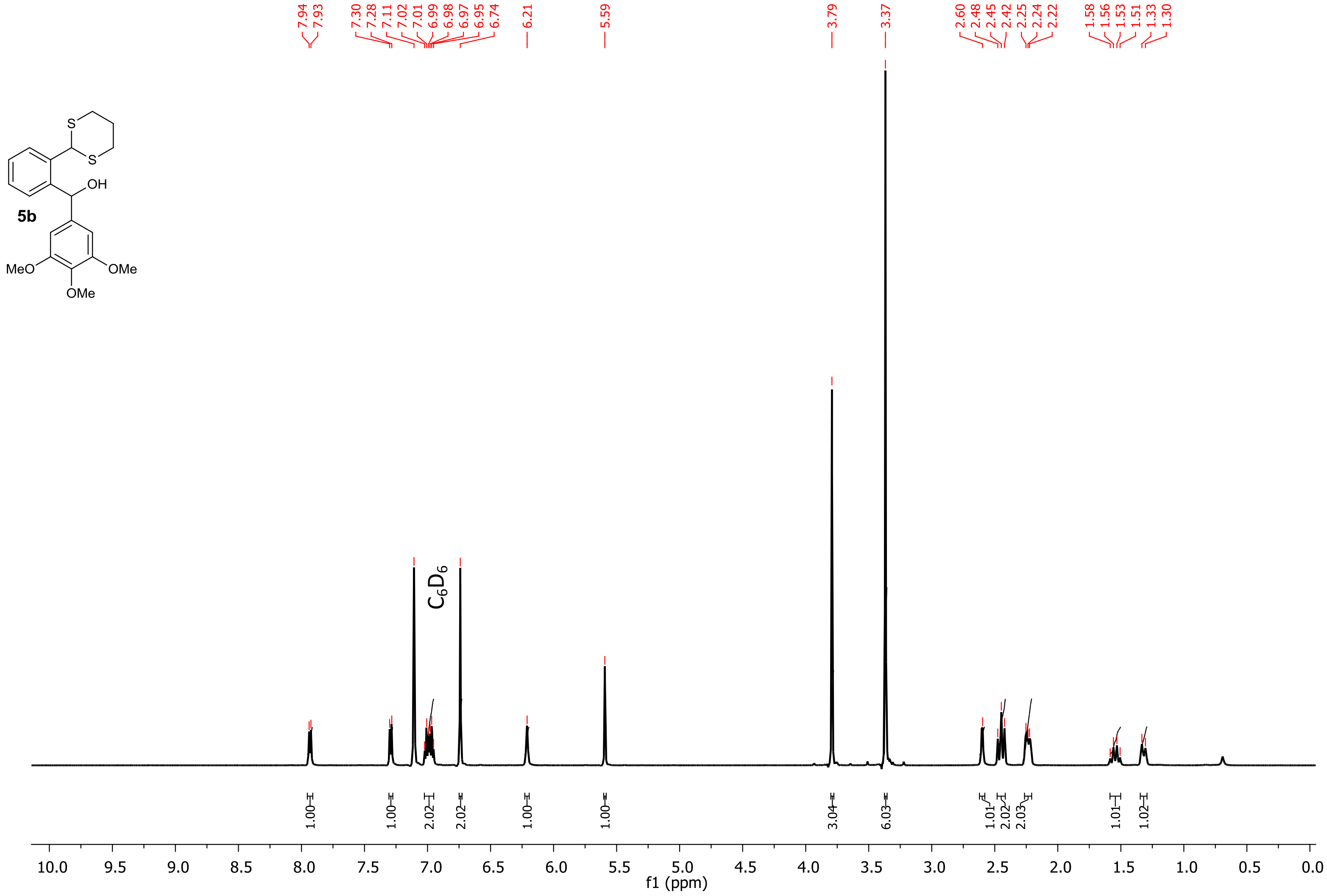

^{13}C NMR spectrum of 2-(1,3-dithian-2-yl)phenyl(3,4,5-trimethoxyphenyl)methanol (5b) (125 MHz, C_6D_6).

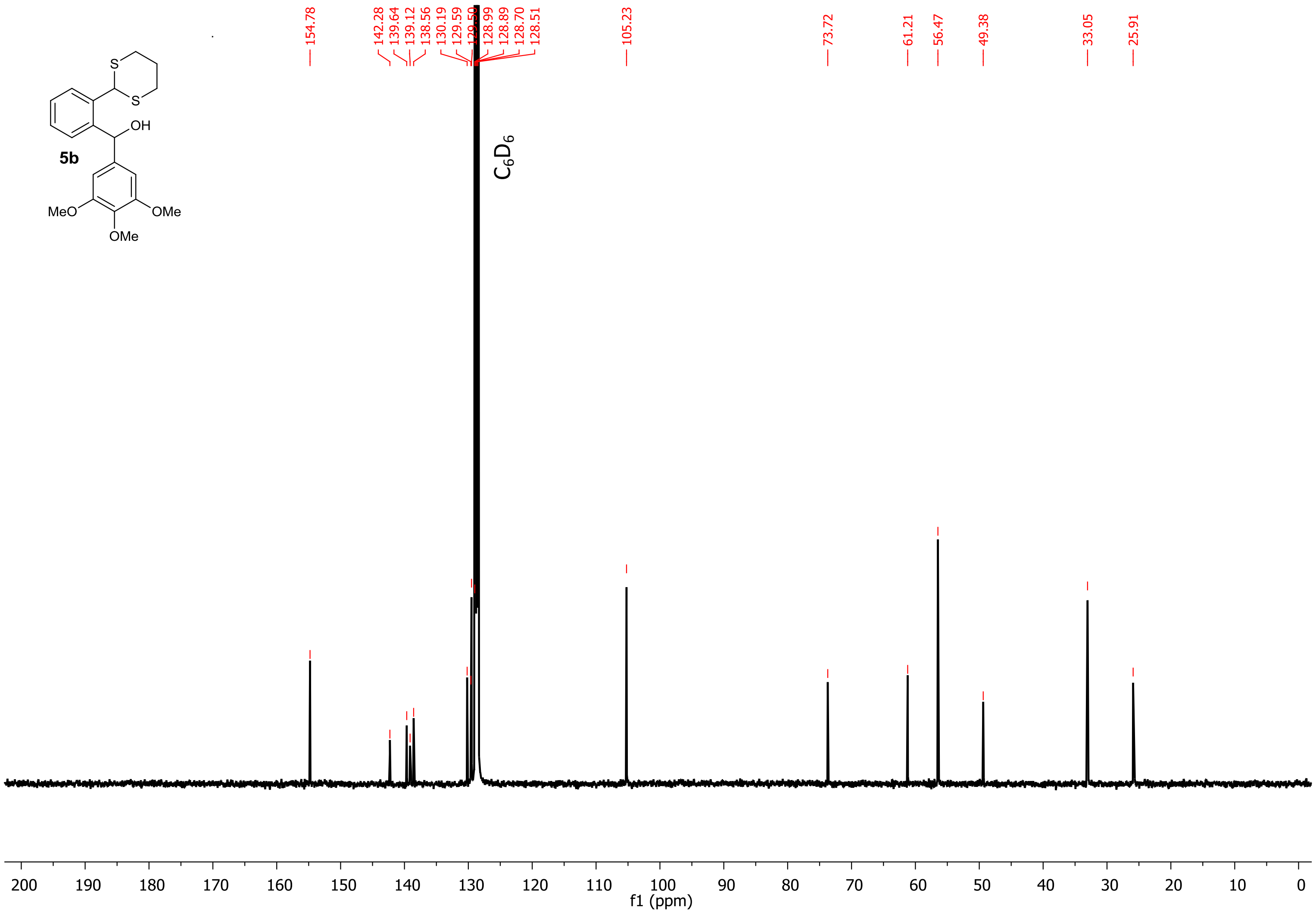

^{1}H NMR spectrum of (2-(1,3-dithian-2-yl)phenyl)(benzo[*b*]thien-2-yl)methanol (5c) (500 MHz, C_6D_6).

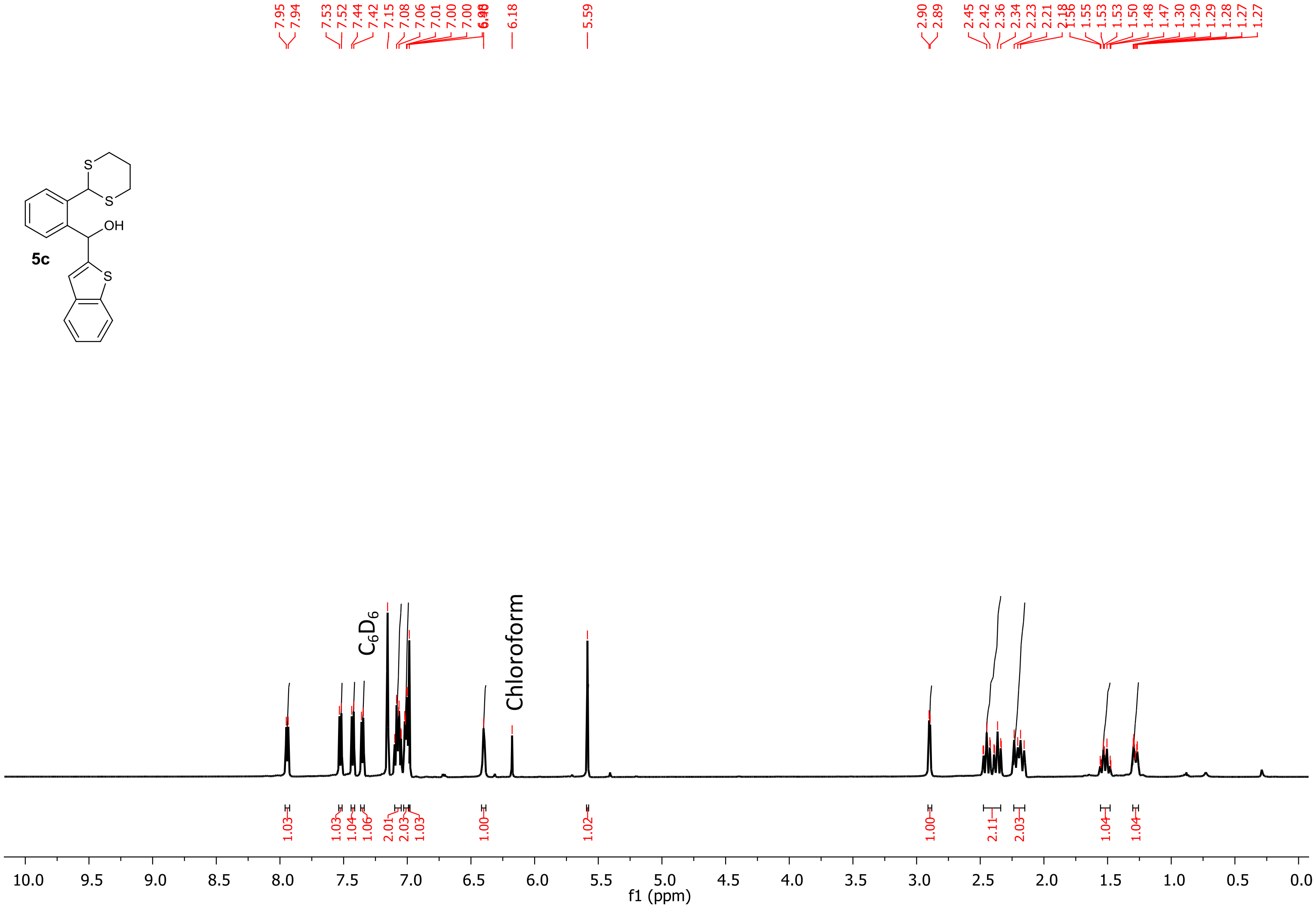

^{13}C NMR spectrum of (2-(1,3-dithian-2-yl)phenyl)(benzo[*b*]thien-2-yl)methanol (5c) (125 MHz, C_6D_6).

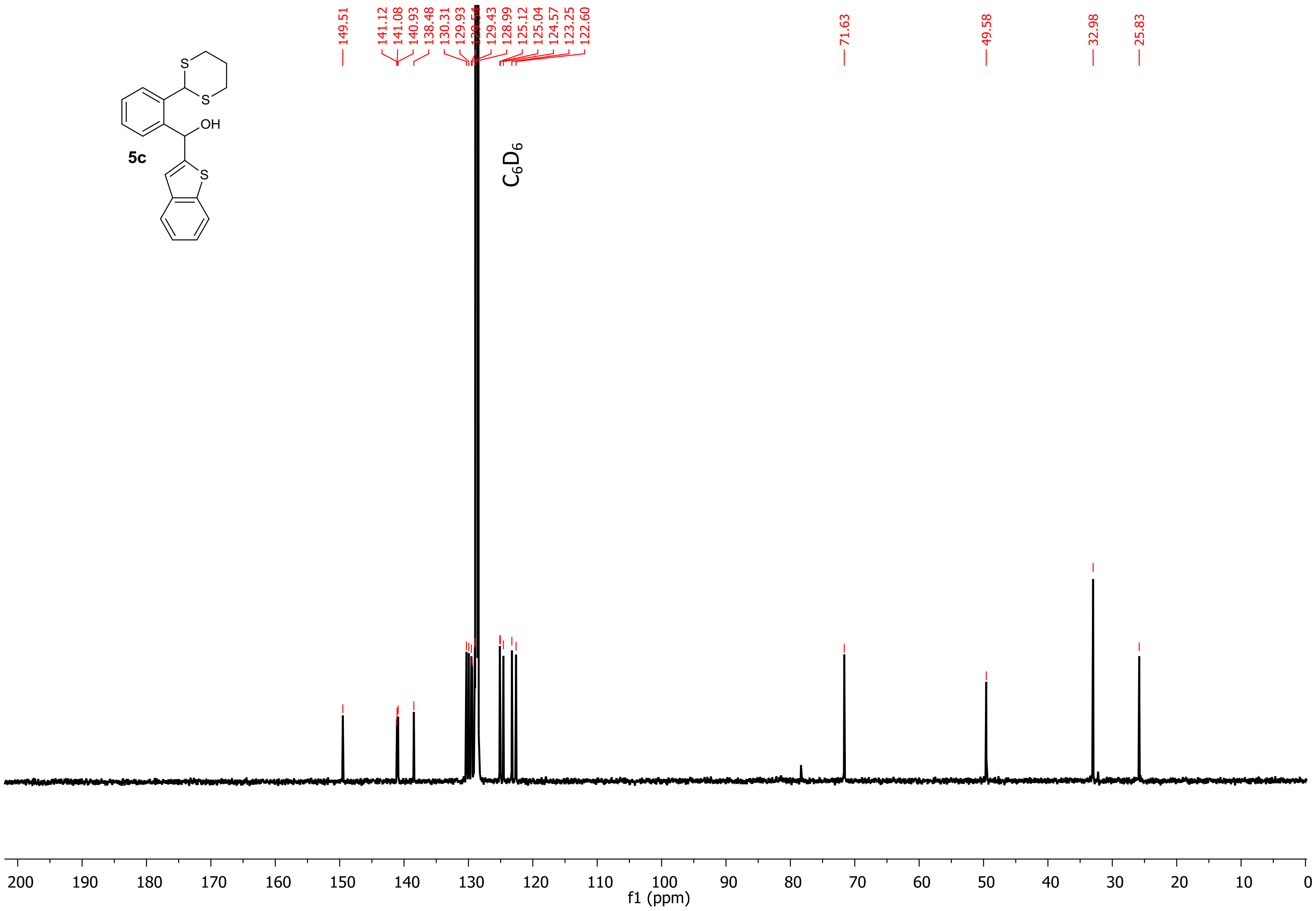

^{1}H NMR spectrum of 2-(1,3-dithian-2-yl)phenyl(thien-2-yl)methanol (5d) (500 MHz, C_6D_6).

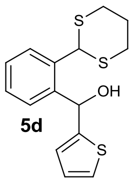

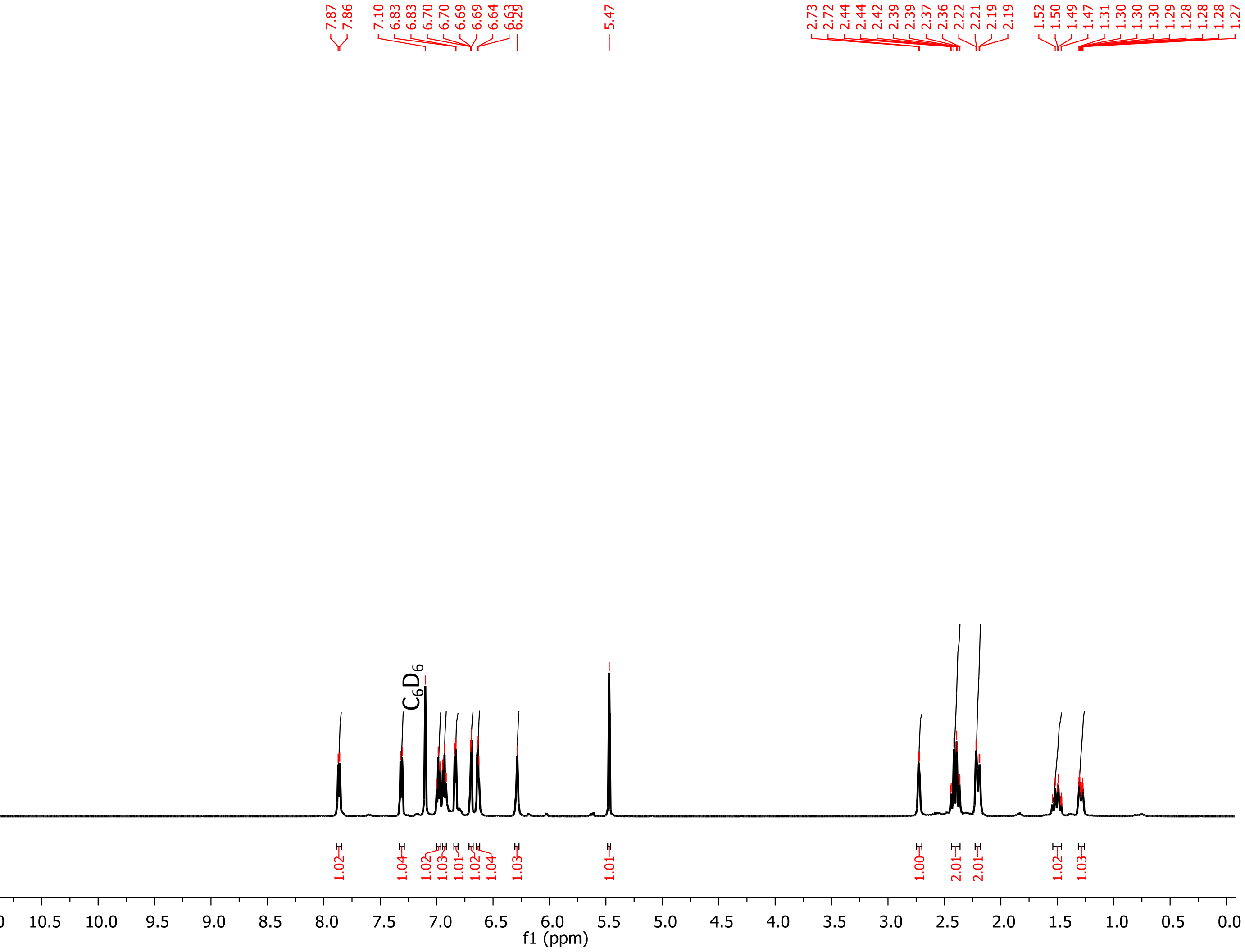

^{13}C NMR spectrum of 2-(1,3-dithian-2-yl)phenyl(thien-2-yl)methanol (5d) (125 MHz, C_6D_6).

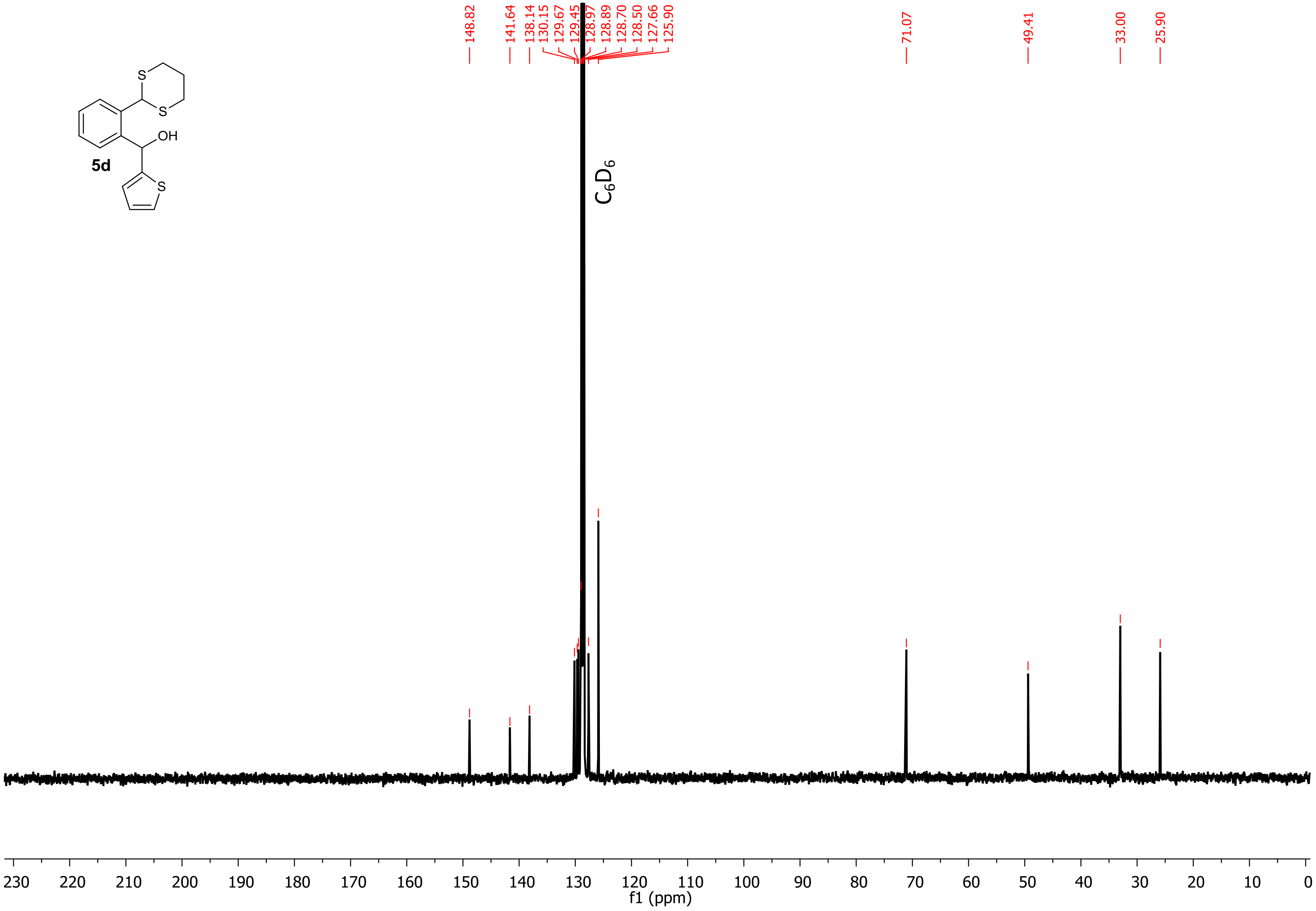

^{1}H NMR spectrum of (2-(1,3-dithian-2-yl)phenyl)(1-methyl-1*H*-indol-2-yl)methanol (5e) (500 MHz, C_6D_6).

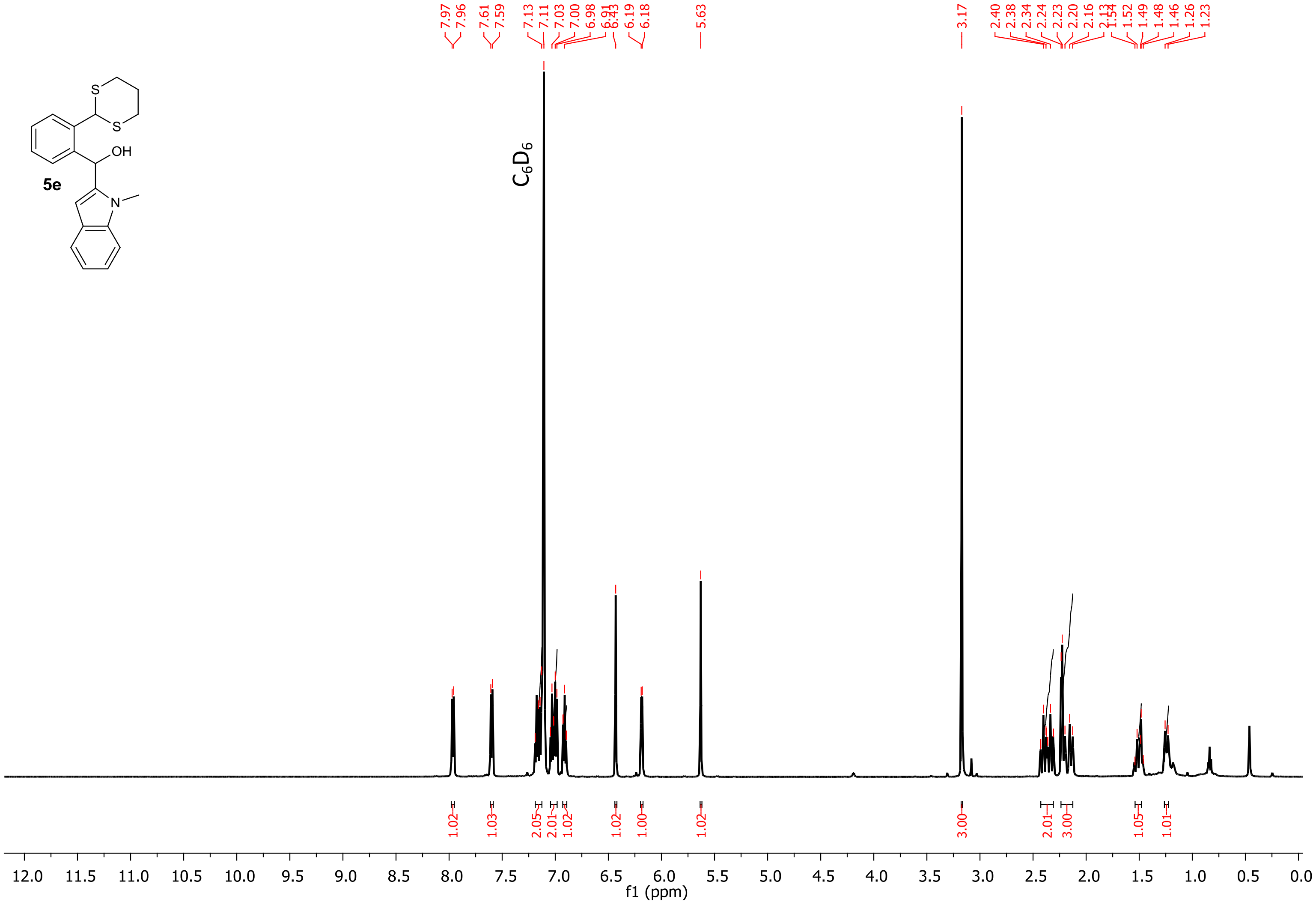

^{13}C NMR spectrum of (2-(1,3-dithian-2-yl)phenyl)(1-methyl-1*H*-indol-2-yl)methanol (5e) (125 MHz, C_6D_6).

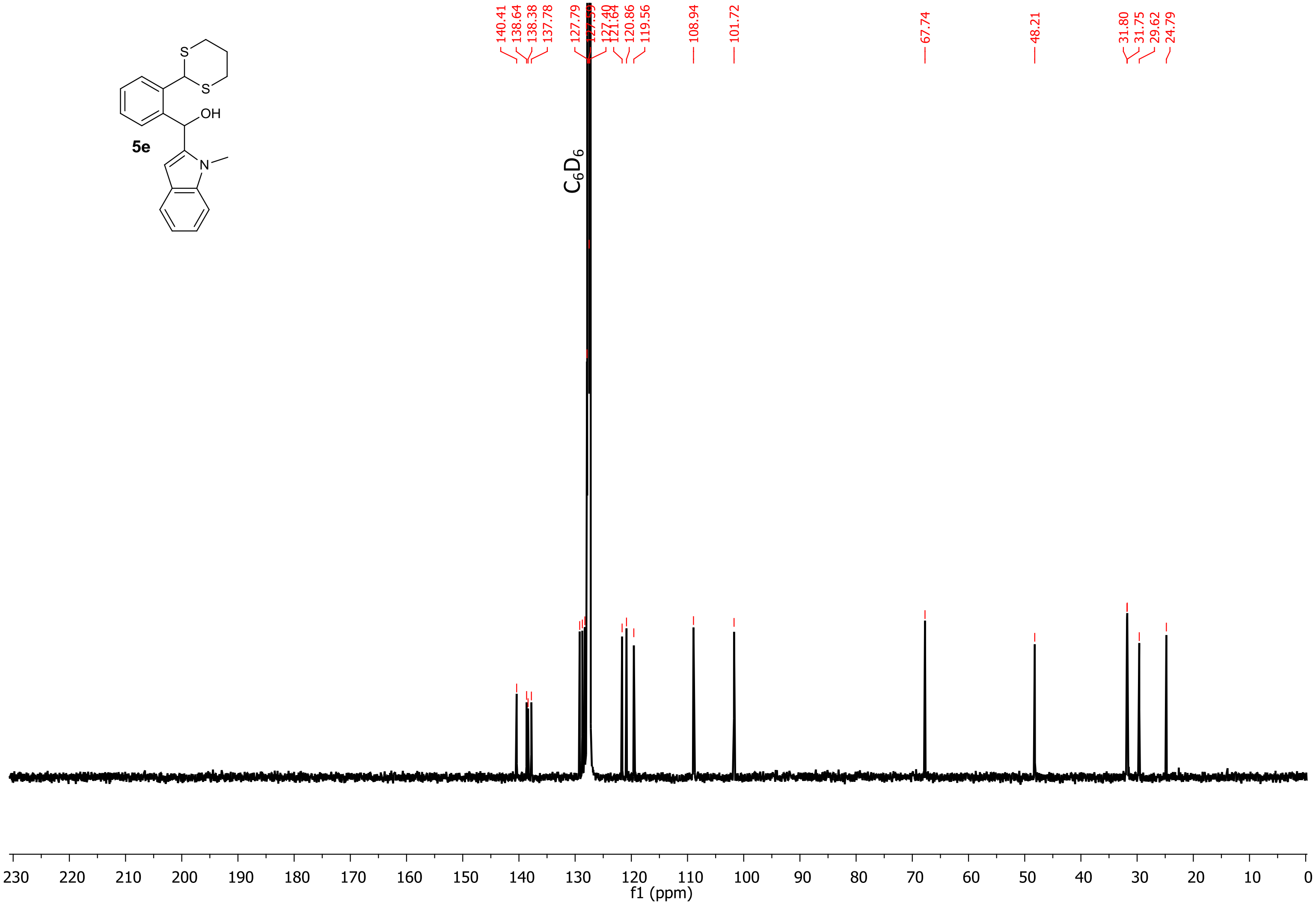

^{1}H NMR spectrum of (2-(1,3-dithian-2-yl)phenyl)(4-(diphenylamino)phenyl)methanol (5f) (500 MHz, C_6D_6).

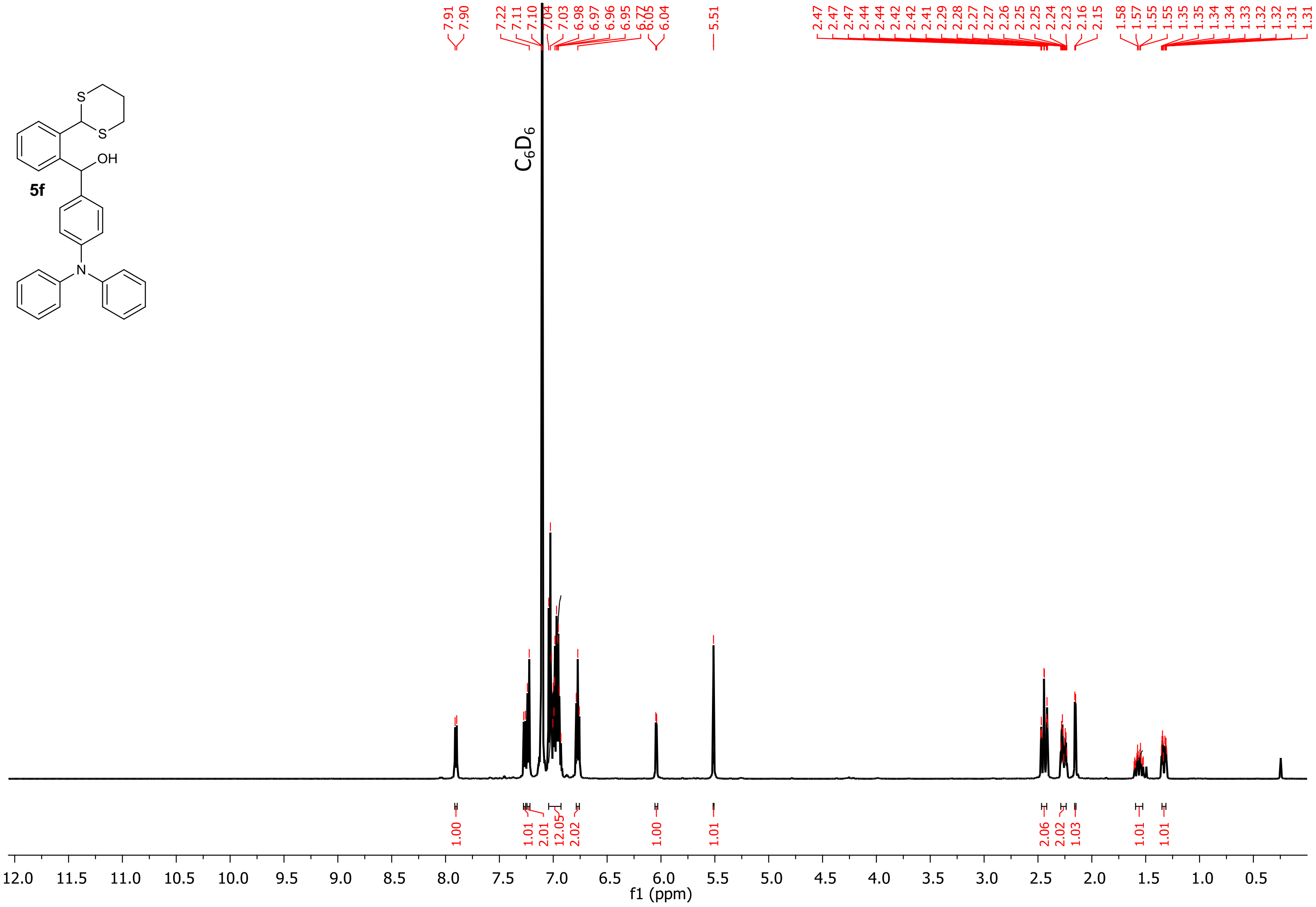

^{13}C NMR spectrum of (2-(1,3-dithian-2-yl)phenyl)(4-(diphenylamino)phenyl)methanol (5f) (125 MHz, C_6D_6).

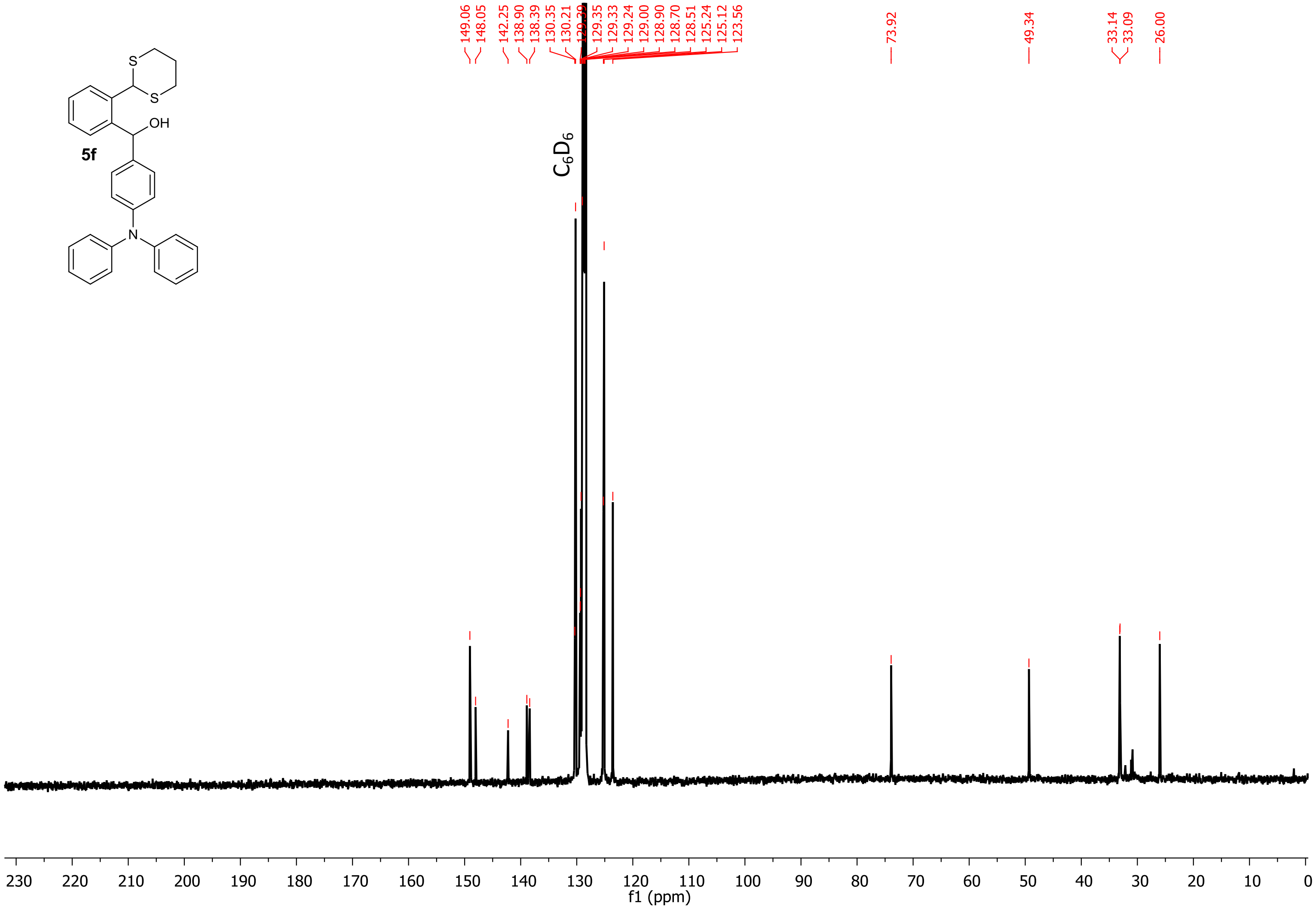

^{1}H NMR spectrum of 3-(2-(1,3-dithian-2-yl)phenyl(9-ethyl-9*H*-carbazol-3-yl)methanol (5g) (500 MHz, C_6D_6).

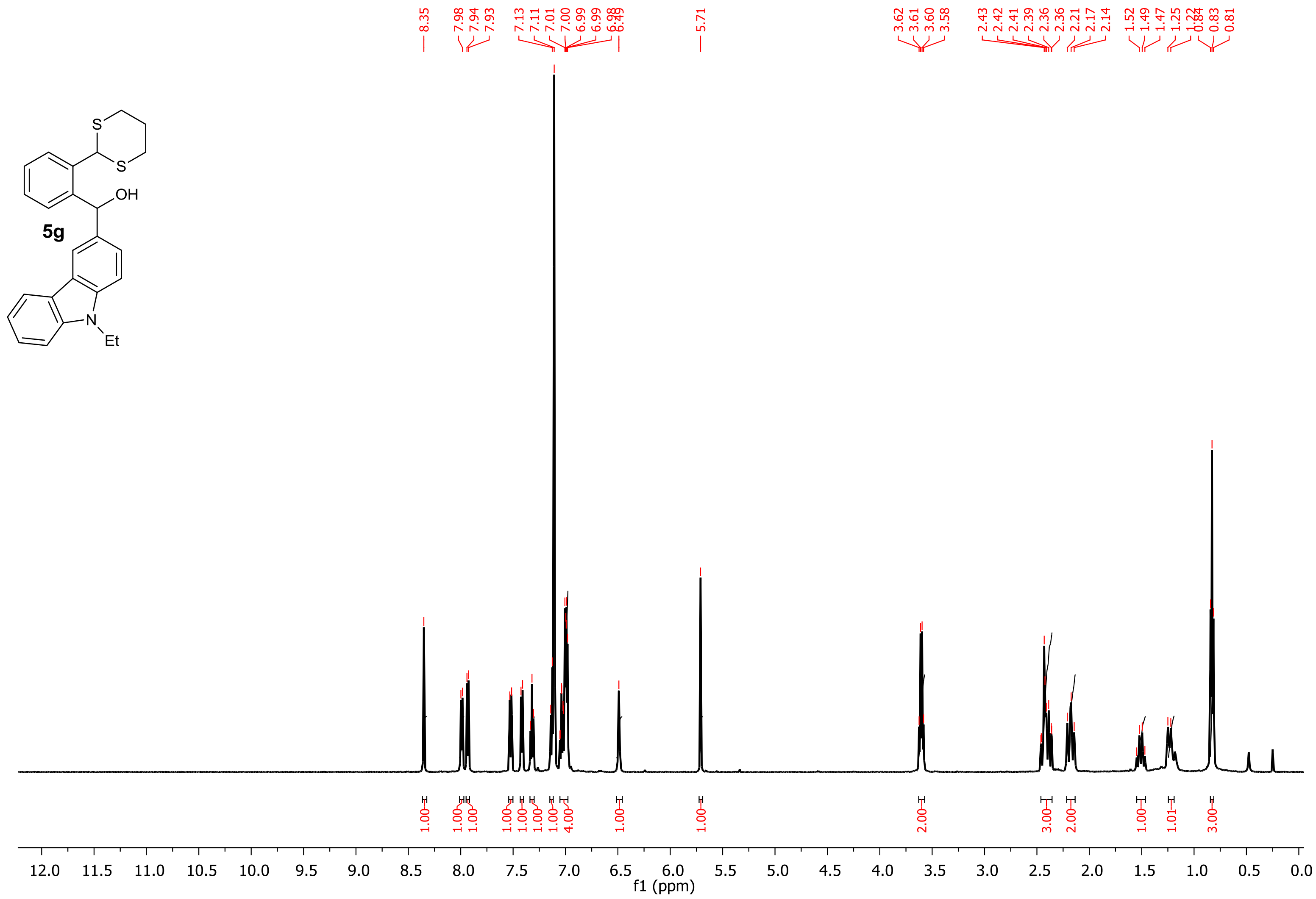

^{13}C NMR spectrum of 3-(2-(1,3-dithian-2-yl)phenyl(9-ethyl-9*H*-carbazol-3-yl)methanol (5g) (125 MHz, C_6D_6).

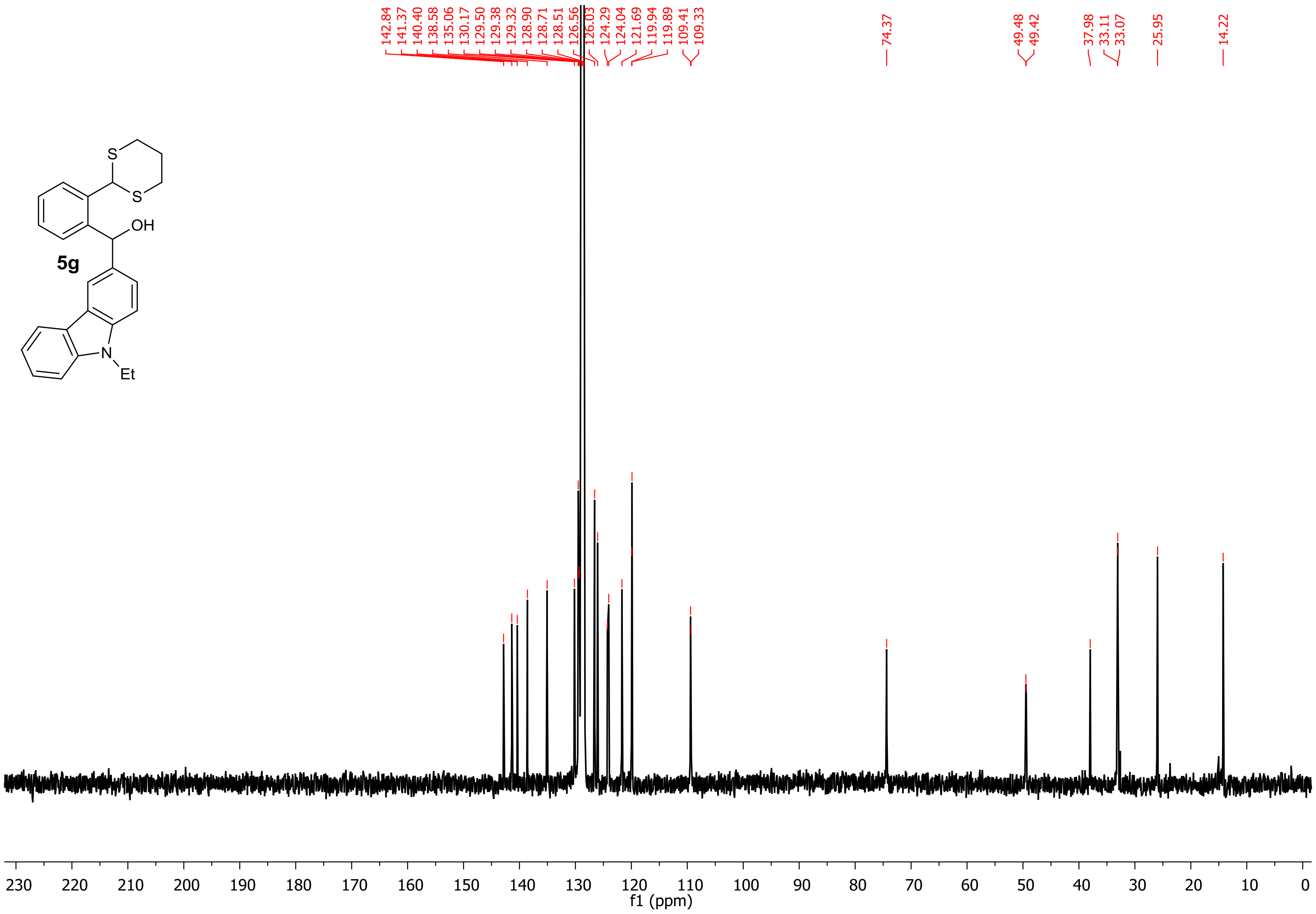

^{1}H NMR spectrum of (2-(1,3-dithian-2-yl)phenyl)(4-methoxyphenyl)methanol (5h) (500 MHz, C_6D_6).

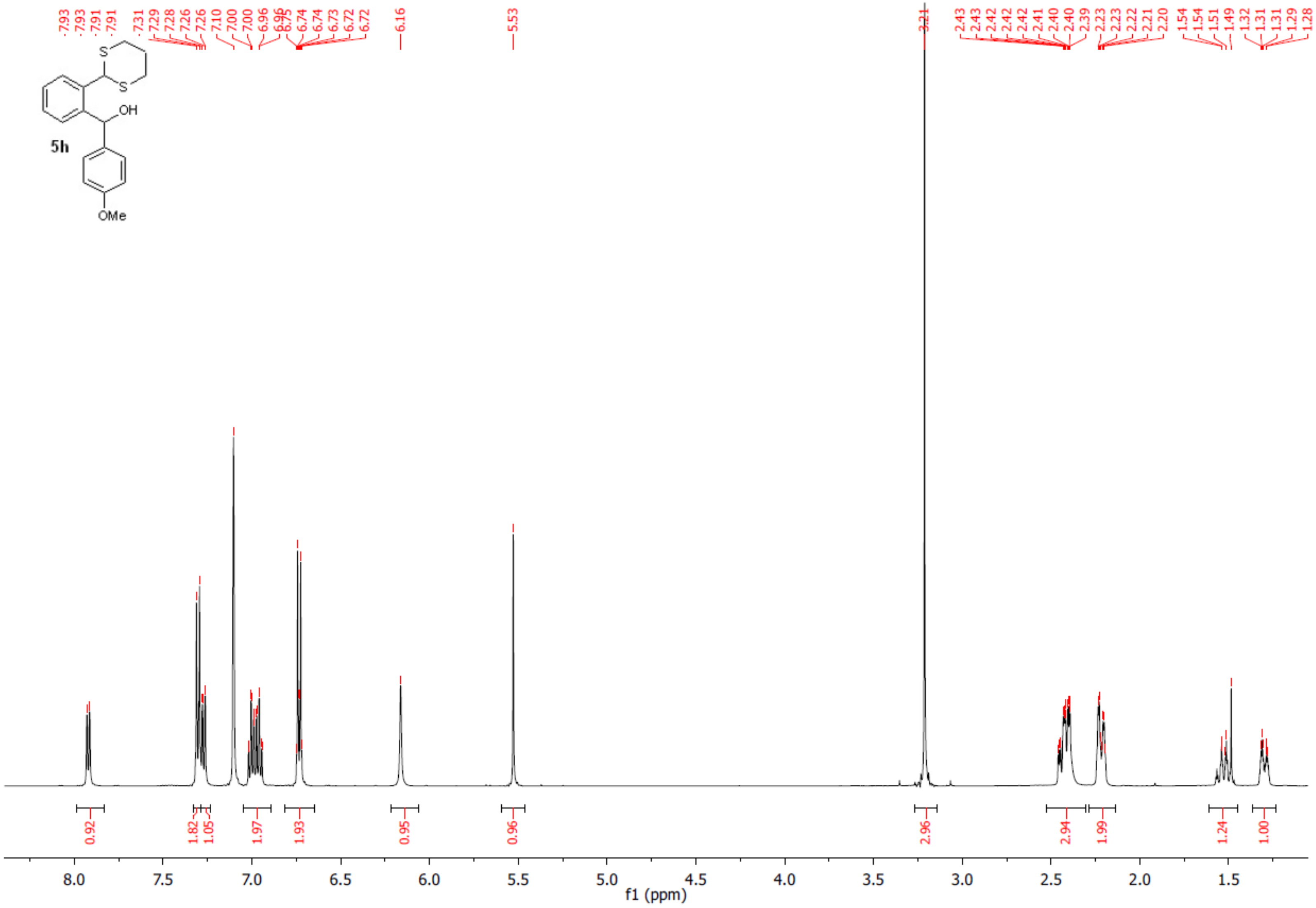

^{13}C NMR spectrum of (2-(1,3-dithian-2-yl)phenyl)(4-methoxyphenyl)methanol (5h) (125 MHz, C_6D_6).

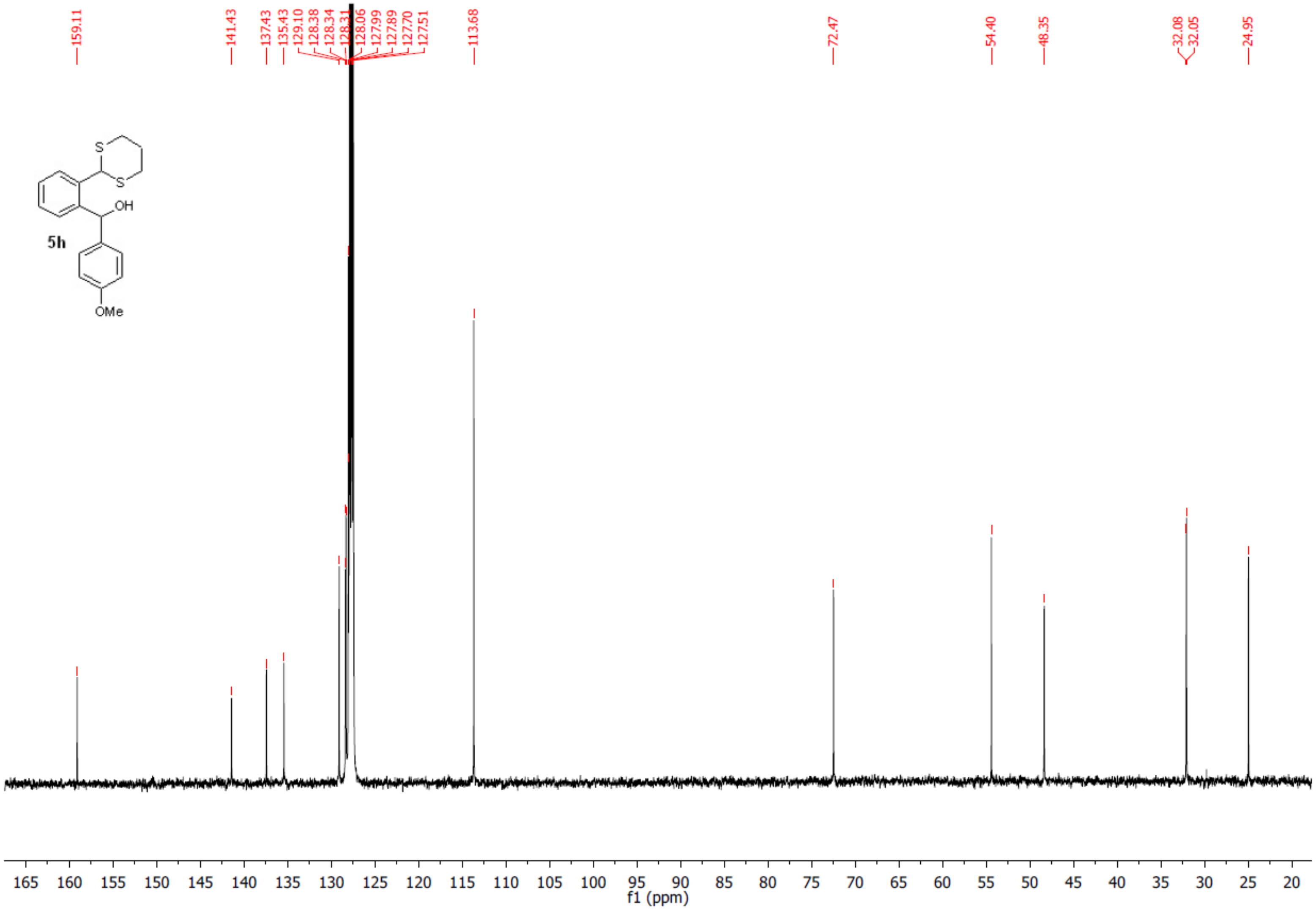

^{1}H NMR spectrum of (6-(1,3)-dithian-2-yl-benzo[*d*][1,3]dioxol-5-yl)(benzo[*d*][1,3]dioxol-5-yl)methanol (6a) (500 MHz, C_6D_6).

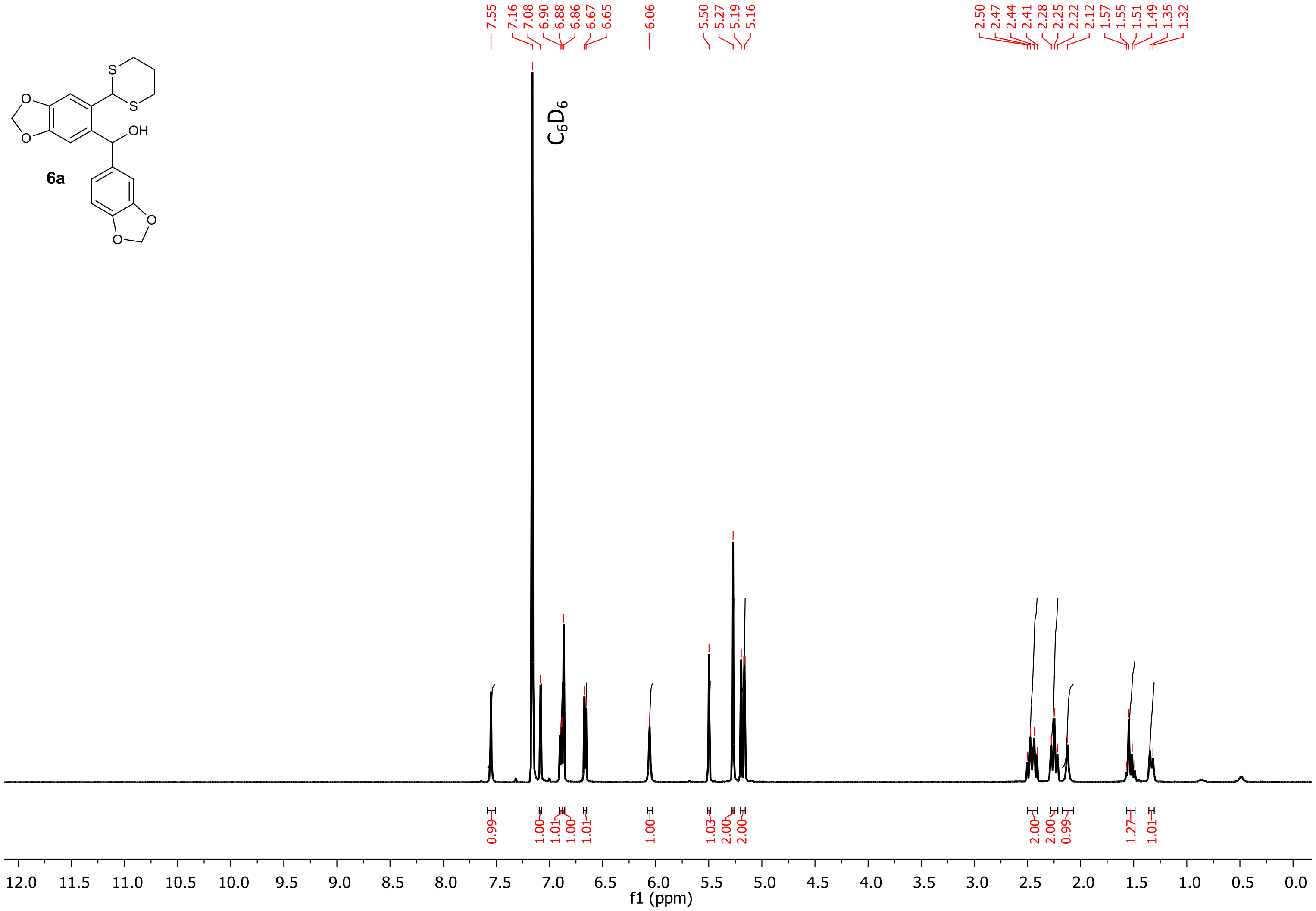

^{13}C NMR spectrum of (6-(1,3)-dithian-2-yl-benzo[*d*][1,3]dioxol-5-yl)(benzo[*d*][1,3]dioxol-5-yl)methanol (6a) (125 MHz, C_6D_6).

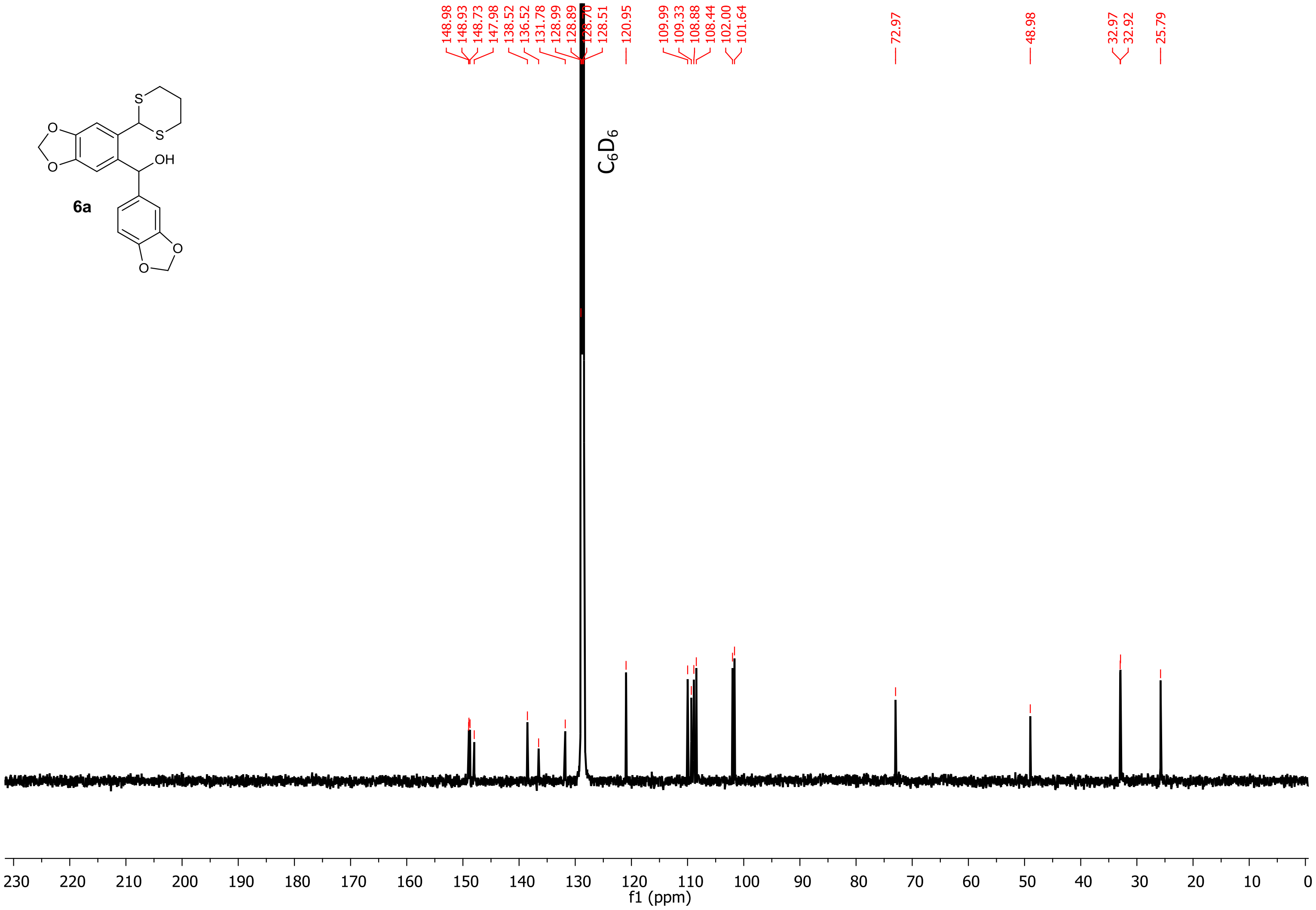

^{1}H NMR spectrum of 6-(1,3-dithian-2-yl)benzo[*d*][1,3]dioxol-5-yl)(3,4,5-trimethoxyphenyl)methanol (6b) (200 MHz, $CDCl_3$).

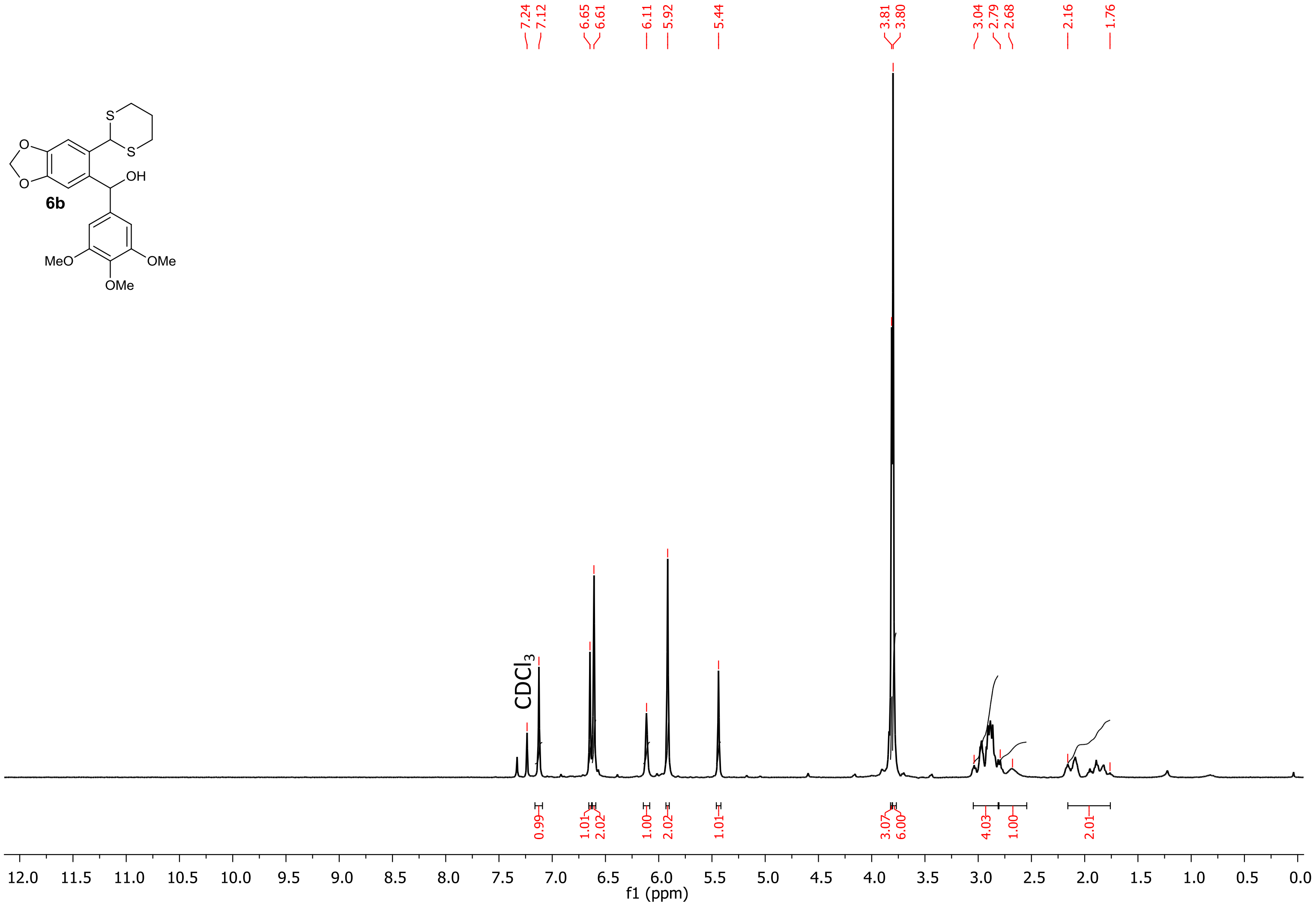

^{13}C NMR spectrum of 6-(1,3-dithian-2-yl)benzo[*d*][1,3]dioxol-5-yl)(3,4,5-trimethoxyphenyl)methanol (6b) (50 MHz, $CDCl_3$).

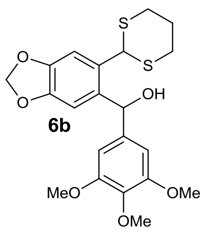

6b

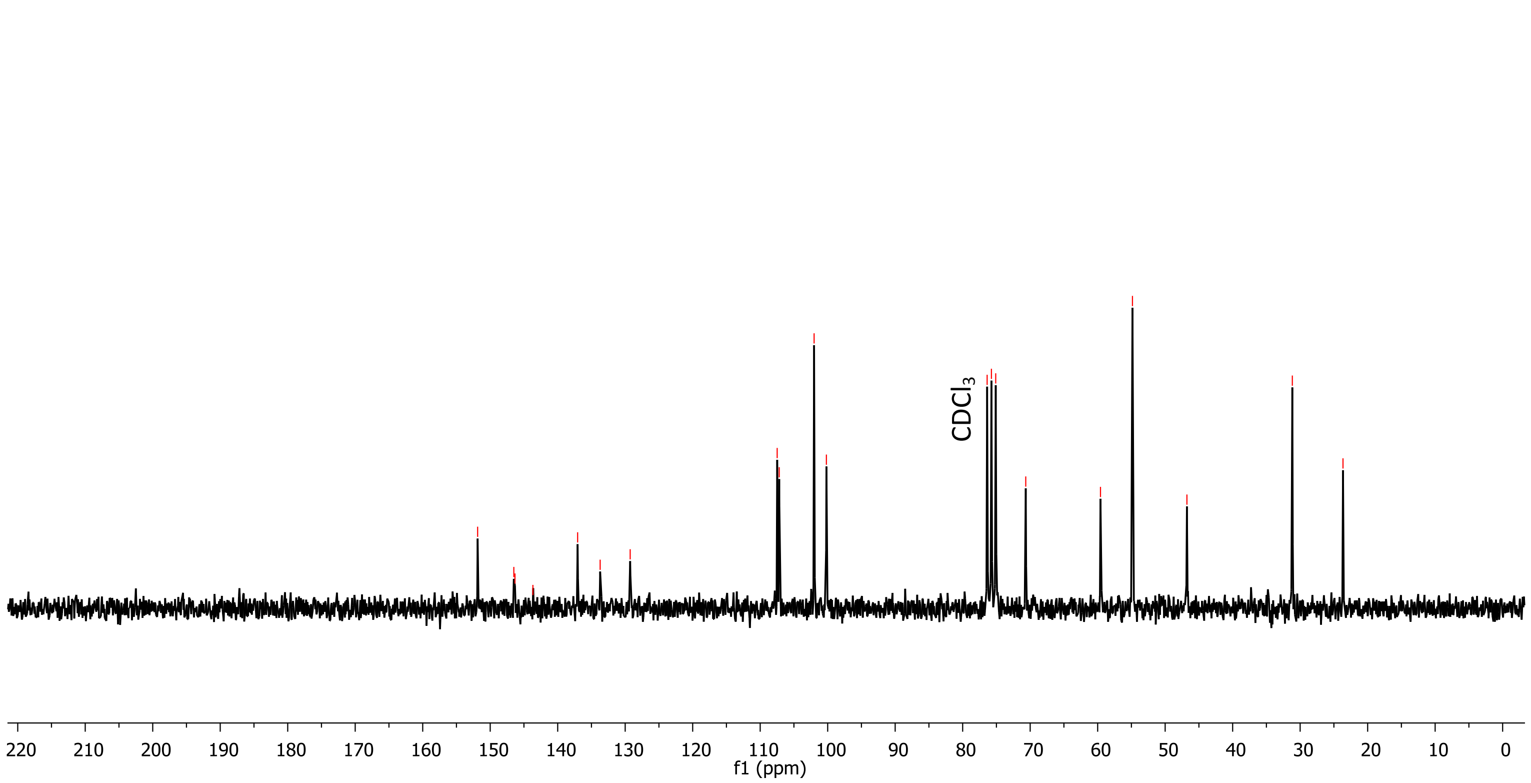

^{1}H NMR spectrum of 5-(1,3-dithian-2-yl)benzyl)benzo[*d*][1,3]dioxole (7a) (500 MHz, C_6D_6).

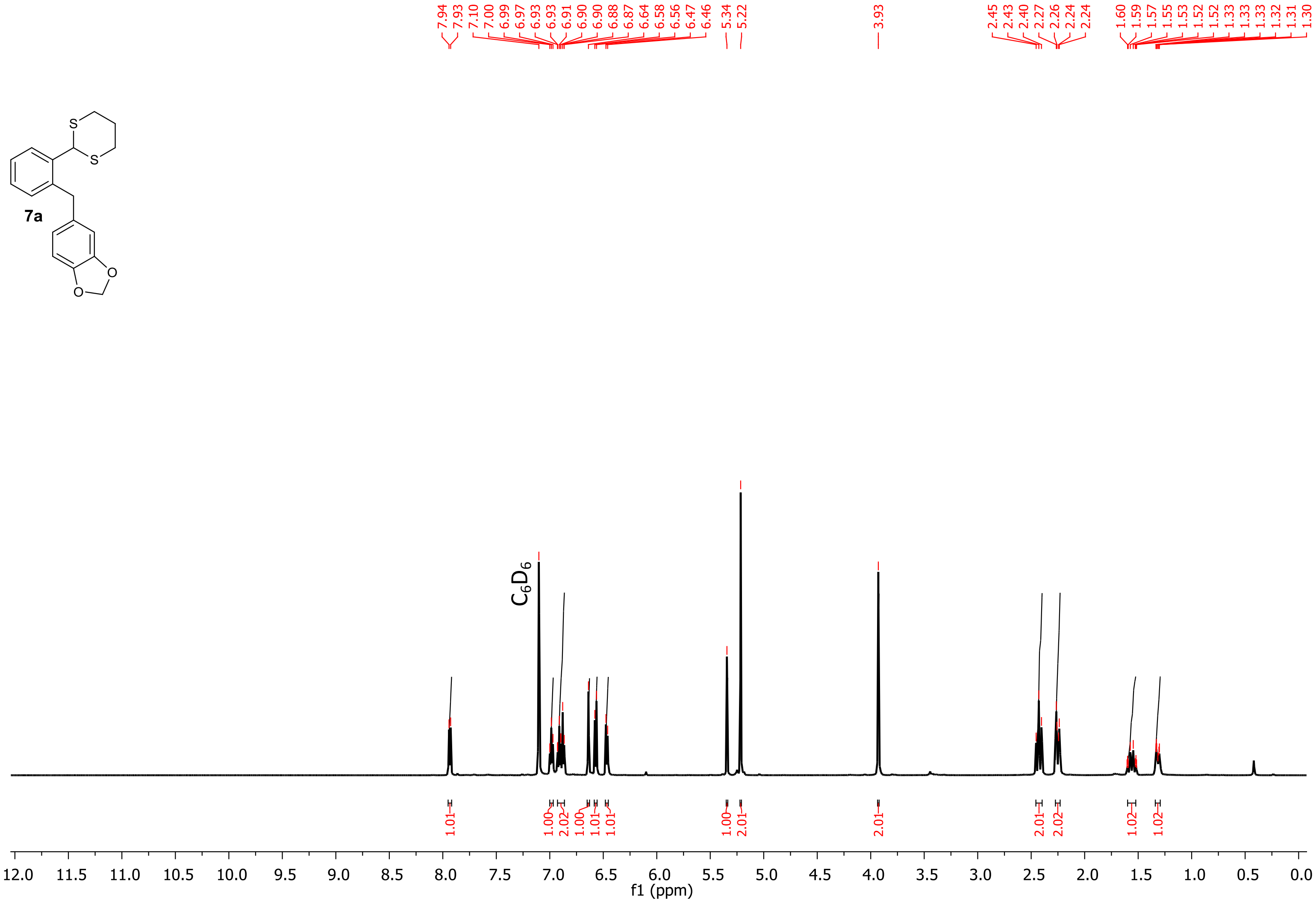

^{13}C NMR spectrum of 5-(1,3-dithian-2-yl)benzyl)benzo[*d*][1,3]dioxole (7a) (125 MHz, C_6D_6).

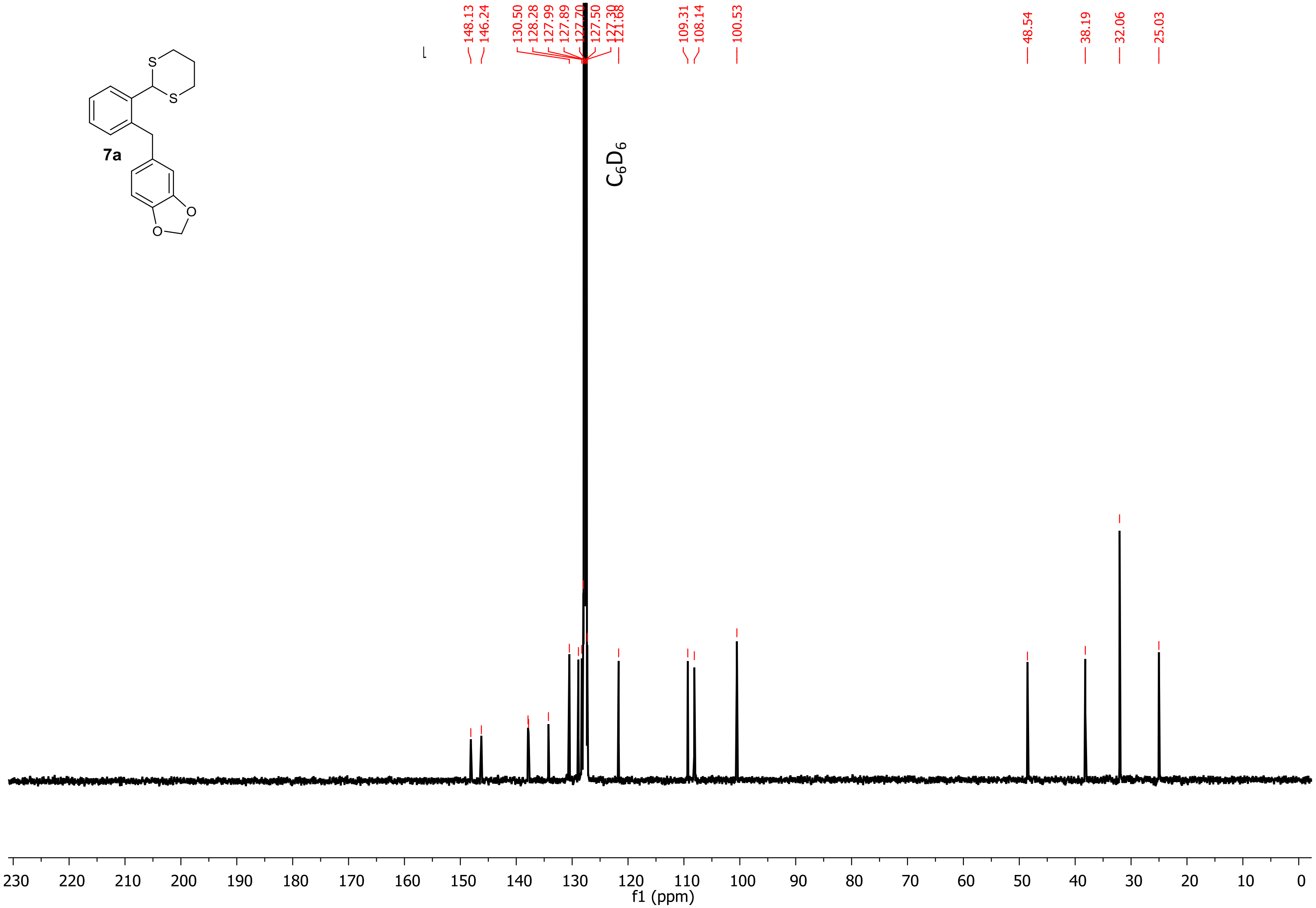

^{1}H NMR spectrum of 2-(2-(3,4,5-trimethoxybenzyl)phenyl)-1,3-dithiane (7b) (500 MHz, C_6D_6).

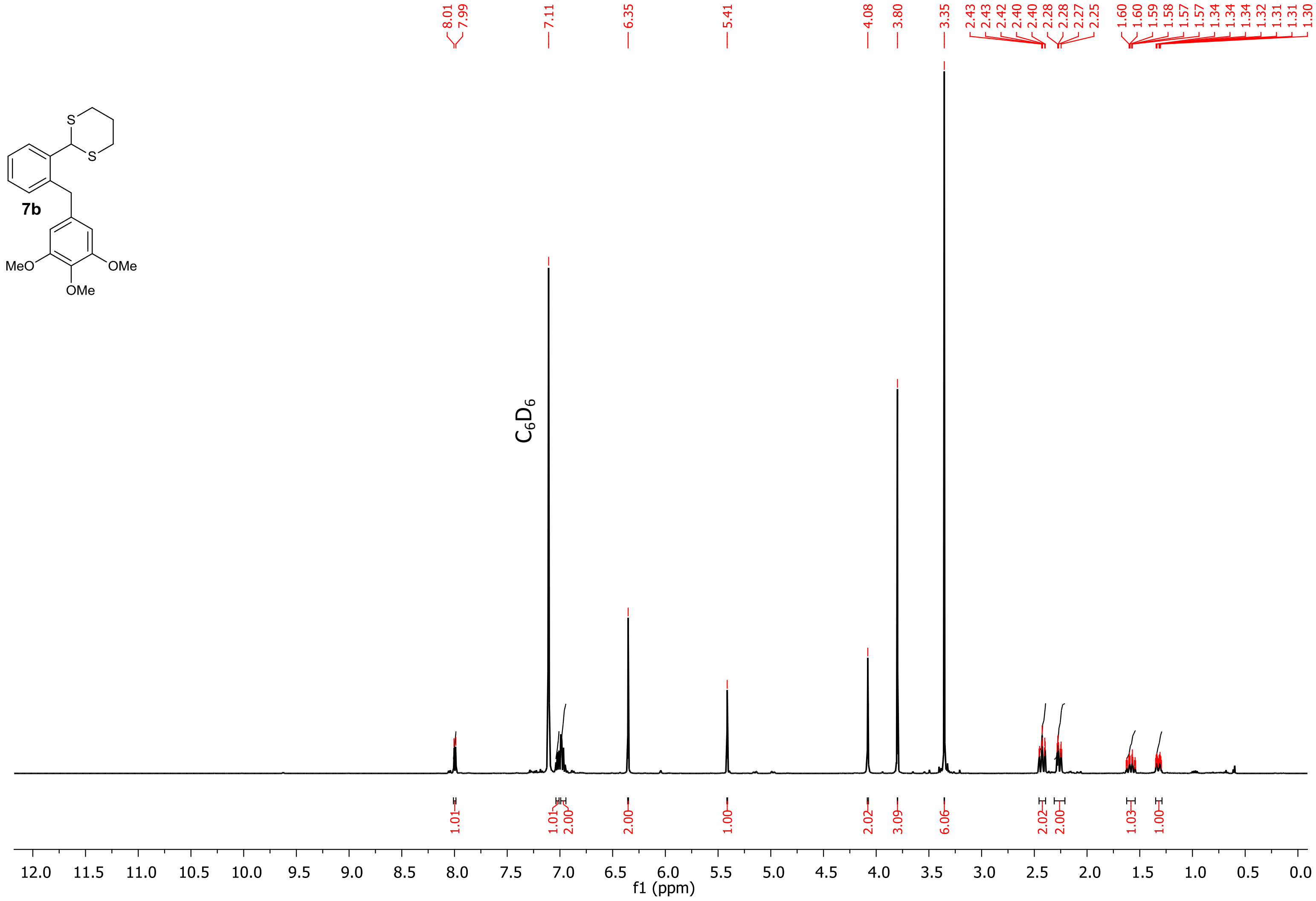

^{13}C NMR spectrum of 2-(2-(3,4,5-trimethoxybenzyl)phenyl)-1,3-dithiane (7b) (125 MHz, C_6D_6).

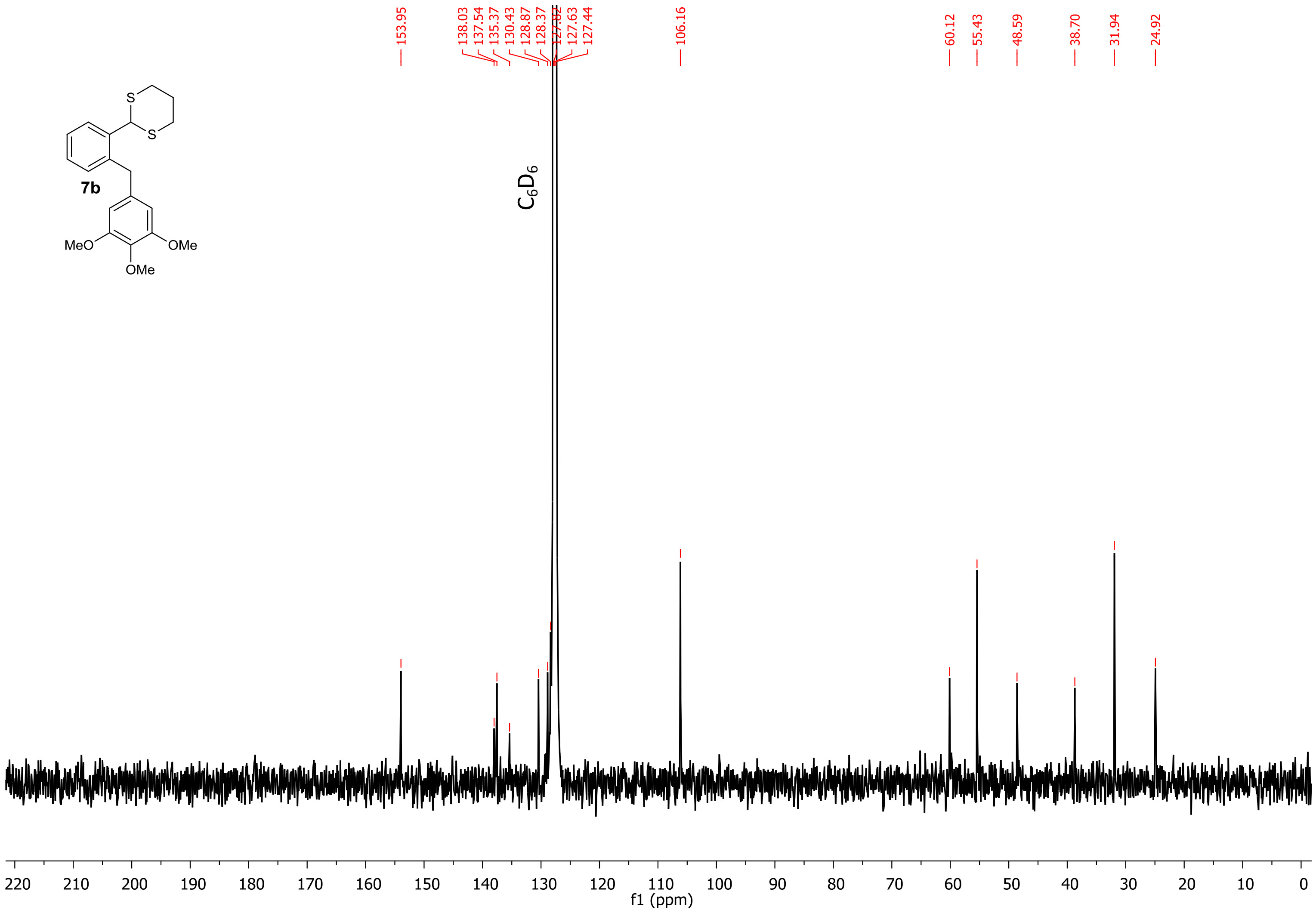

^{1}H NMR spectrum of 2-(2-(1,3)-dithian-2-yl)benzyl)benzo[*b*]thiophene (7c) (500 MHz, C_6D_6).

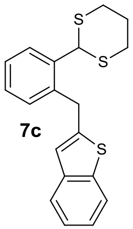

7c

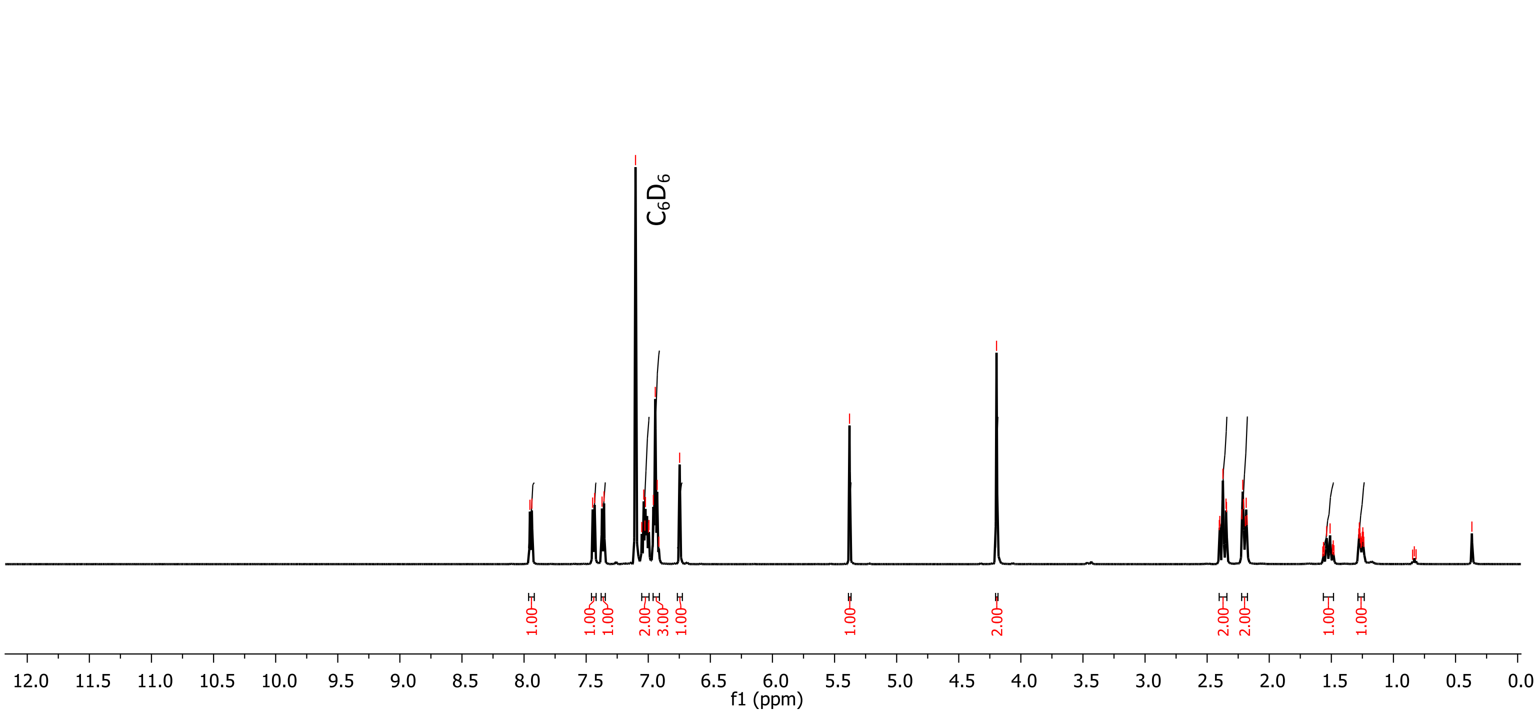

^{13}C NMR spectrum of 2-(2-(1,3)-dithian-2-yl)benzyl)benzo[*b*]thiophene (7c) (125 MHz, C_6D_6).

7c

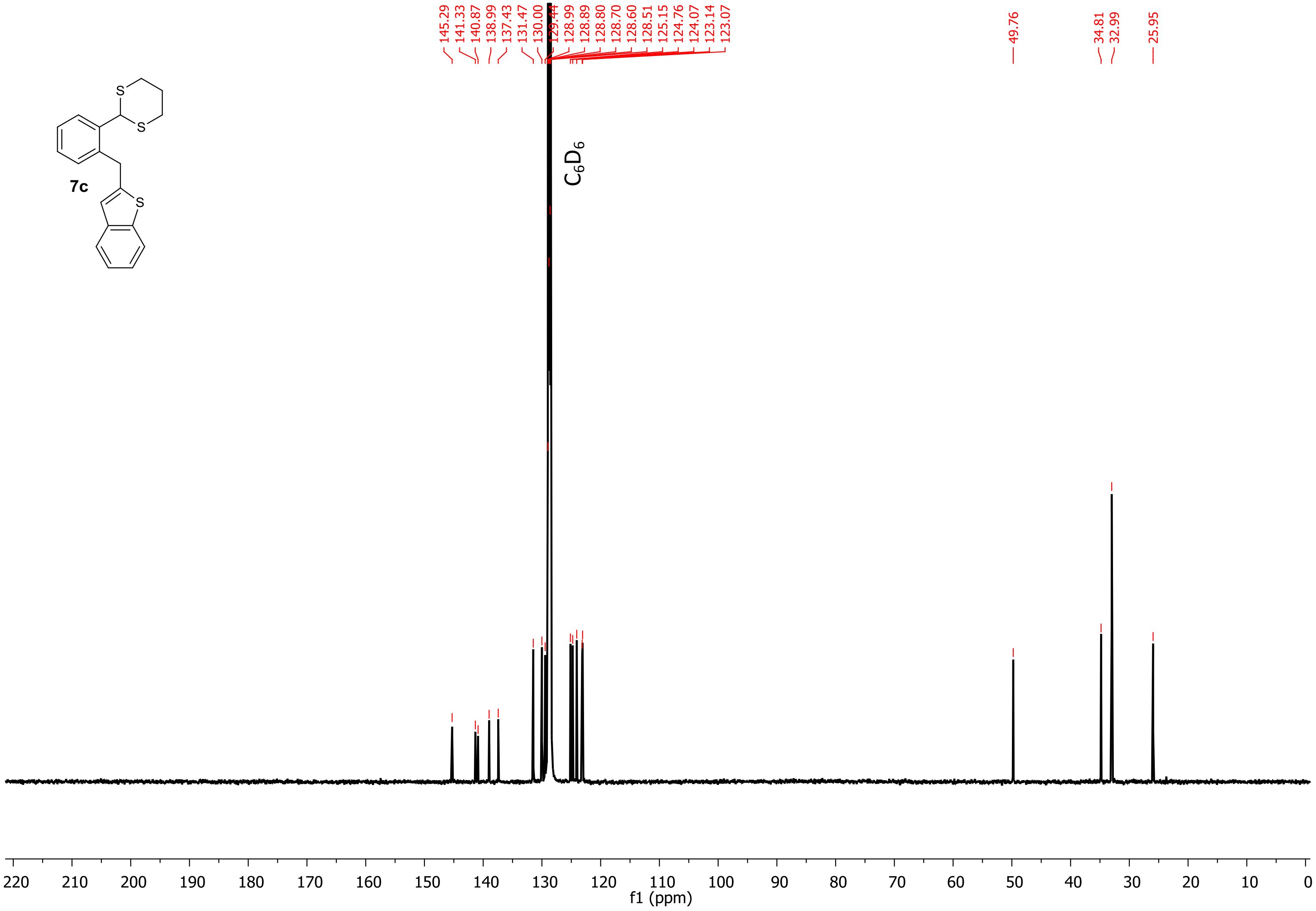

^{1}H NMR spectrum of 2-(2-(thien-2-ylmethyl)phenyl)-1,3-dithiane (7d) (500 MHz, C_6D_6).

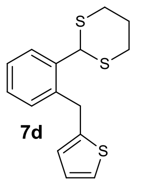

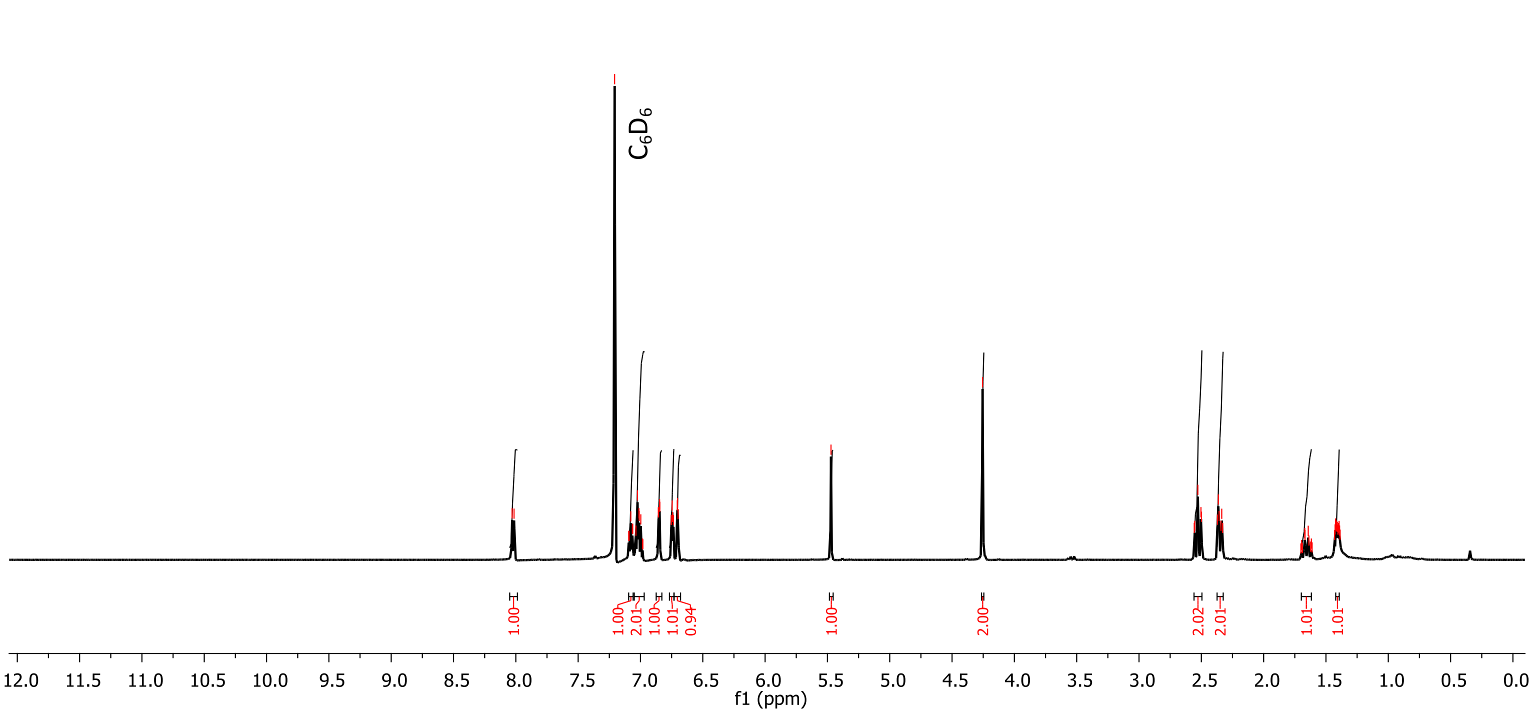

^{13}C NMR spectrum of 2-(2-(thien-2-ylmethyl)phenyl)-1,3-dithiane (7d) (125 MHz, C_6D_6).

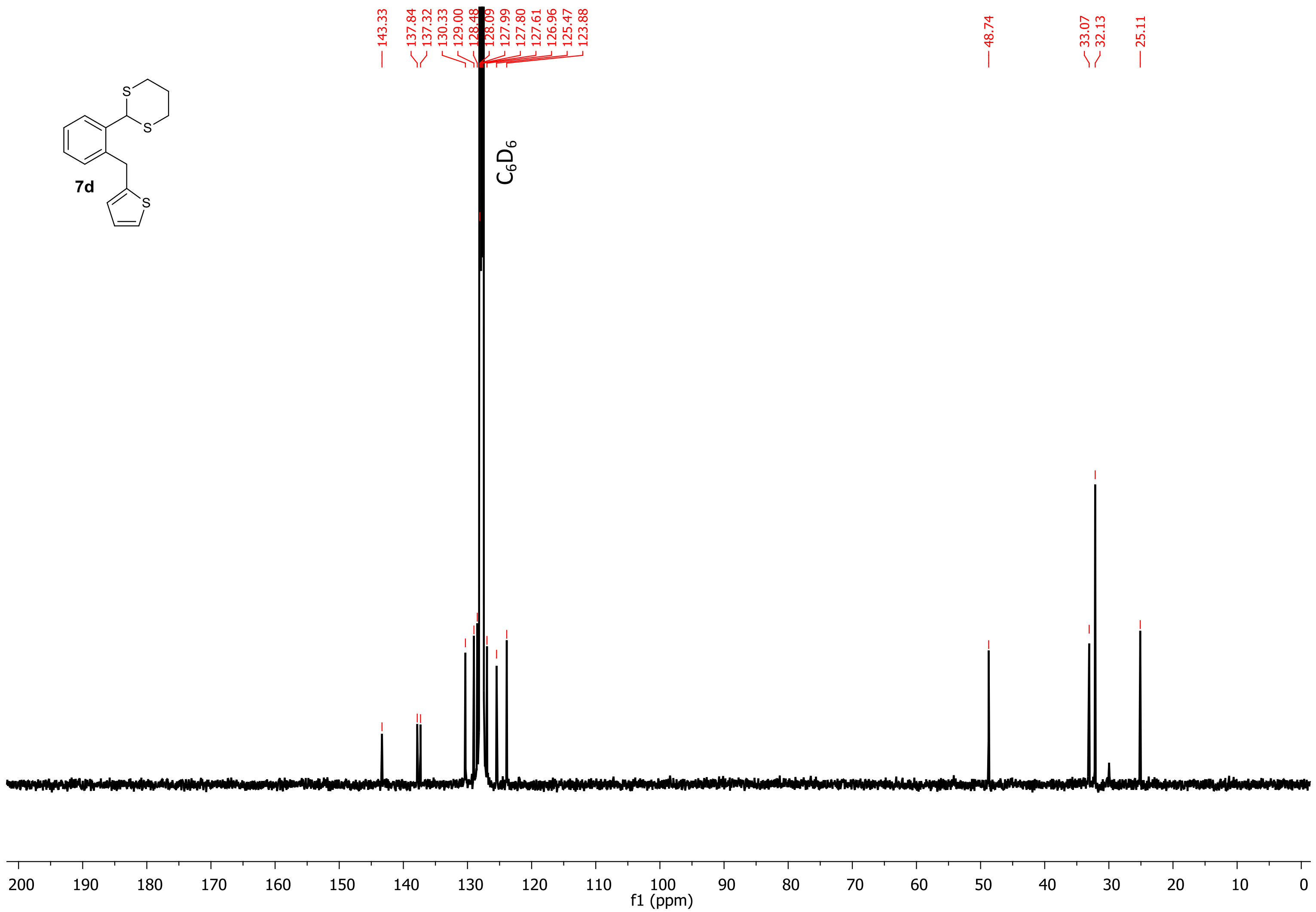

^{1}H NMR spectrum of 2-(2-[1,3]dithian-2-yl-benzyl)-1-methyl-1*H*-indole (7e) (500 MHz, C_6D_6).

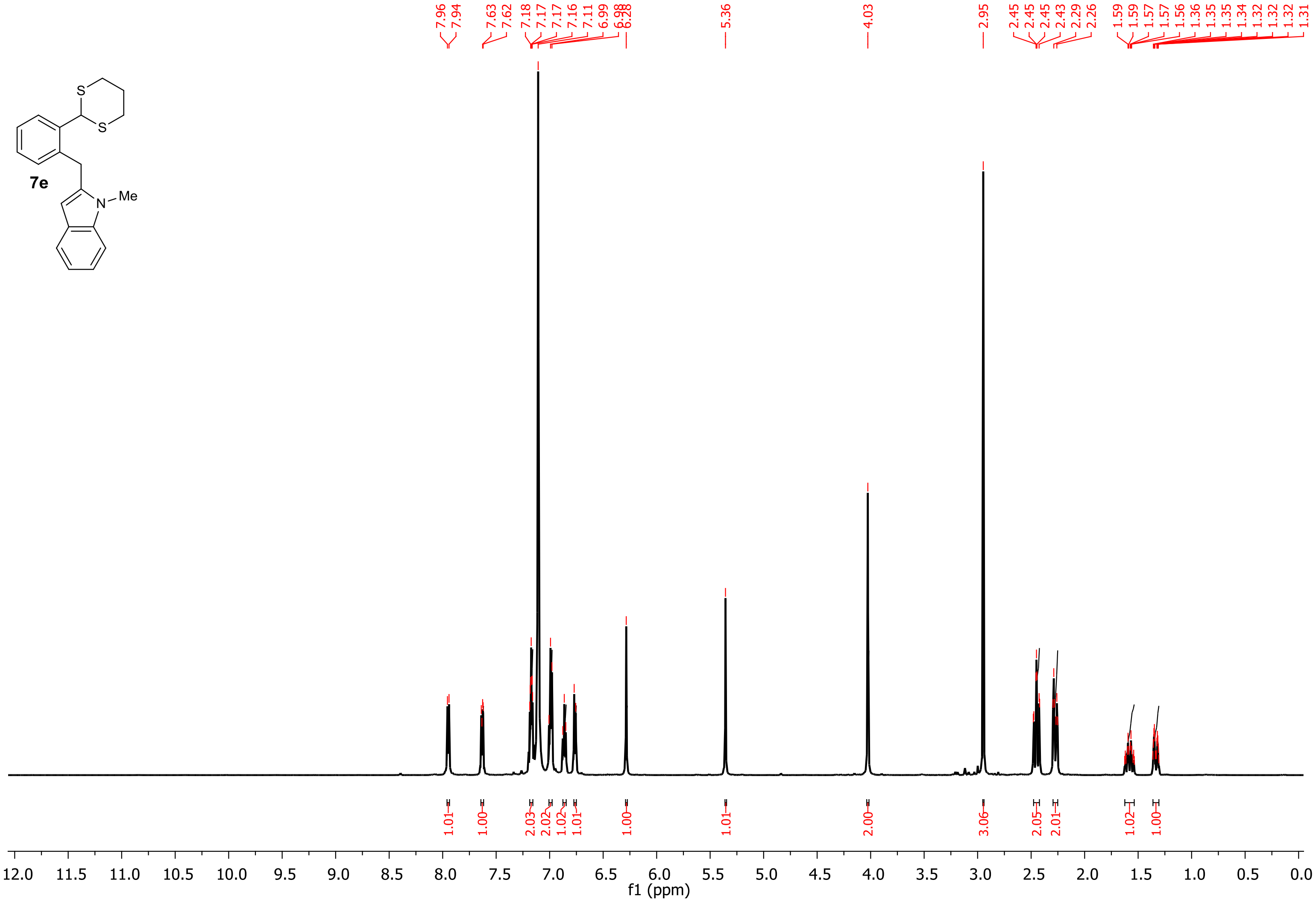

^{13}C NMR spectrum of 2-(2-[1,3]dithian-2-yl-benzyl)-1-methyl-1*H*-indole (7e) (125 MHz, C_6D_6).

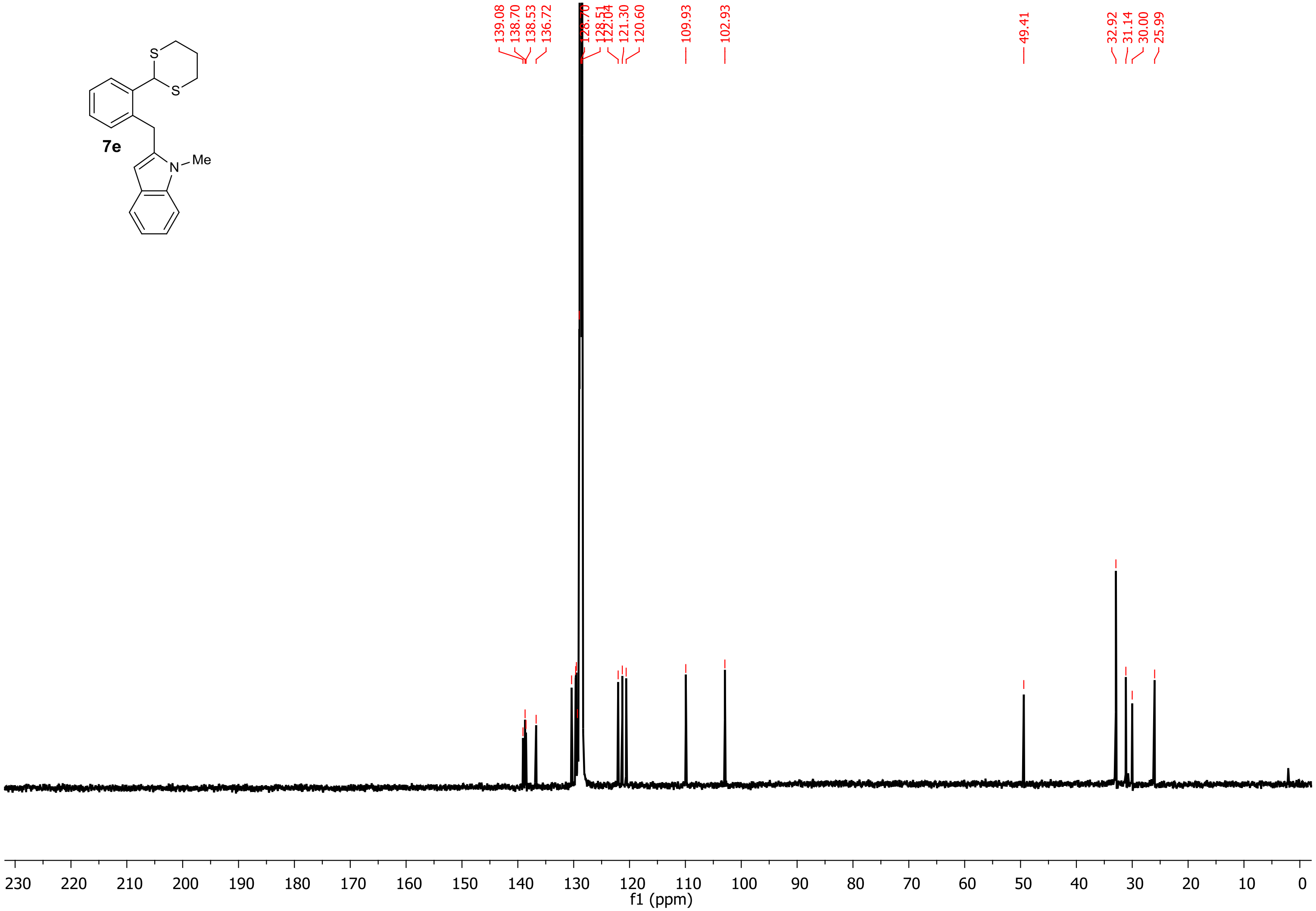

^{1}H NMR spectrum of 2-(4-(diphenylamino)phenylmethyl)phenyl)-1,3-dithiane (7f) (500 MHz, C_6D_6).

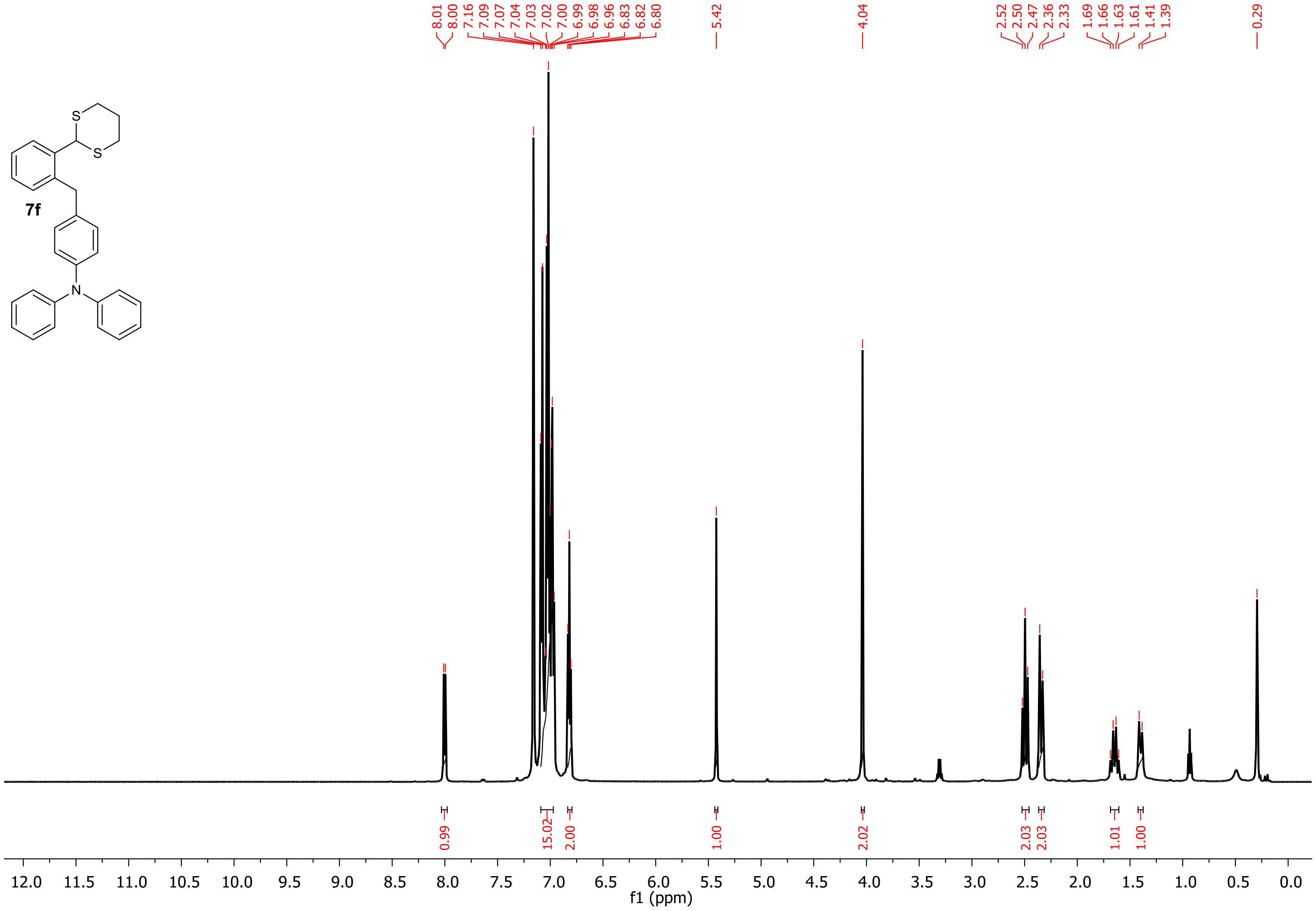

^{13}C NMR spectrum of 2-(4-(diphenylamino)phenylmethyl)phenyl)-1,3-dithiane (7f) (125 MHz, C_6D_6).

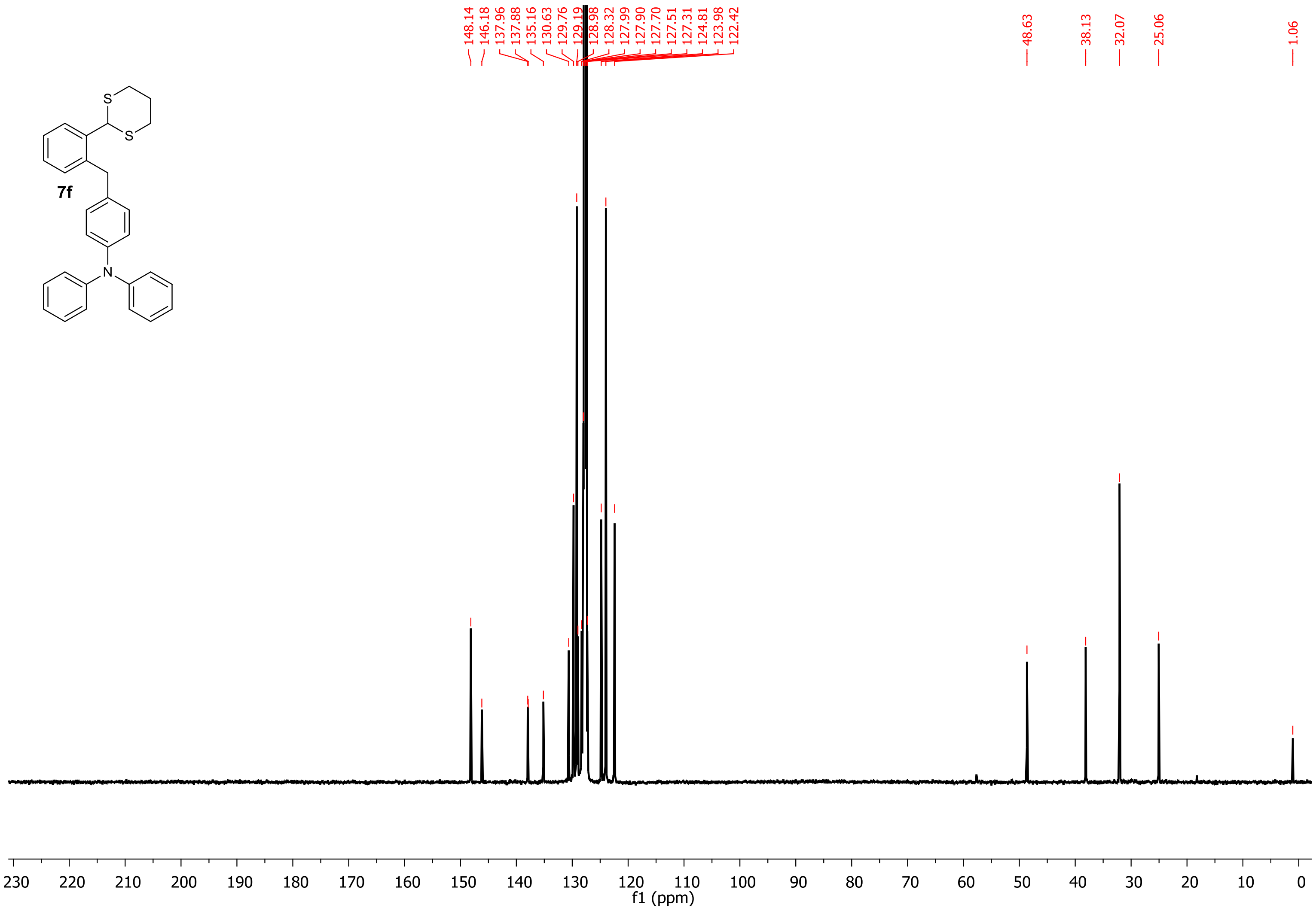

^{1}H NMR spectrum of 3-(2-[1,3]dithian-2-yl-benzyl)-9-ethyl-9*H*-carbazole (7g) (500 MHz, C_6D_6).

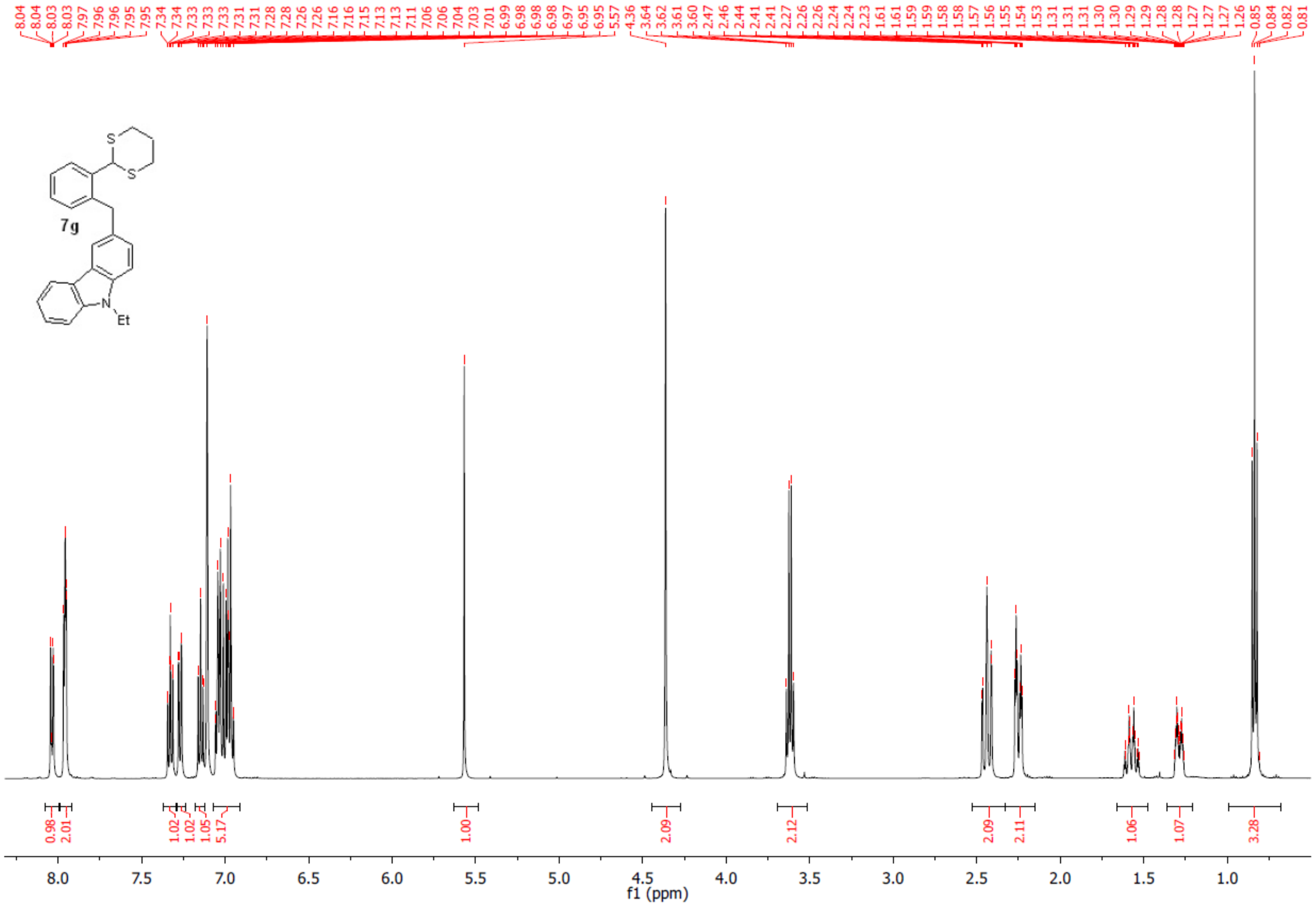

^{13}C NMR spectrum of 3-(2-[1,3]dithian-2-yl-benzyl)-9-ethyl-9*H*-carbazole (7g) (125 MHz, C_6D_6).

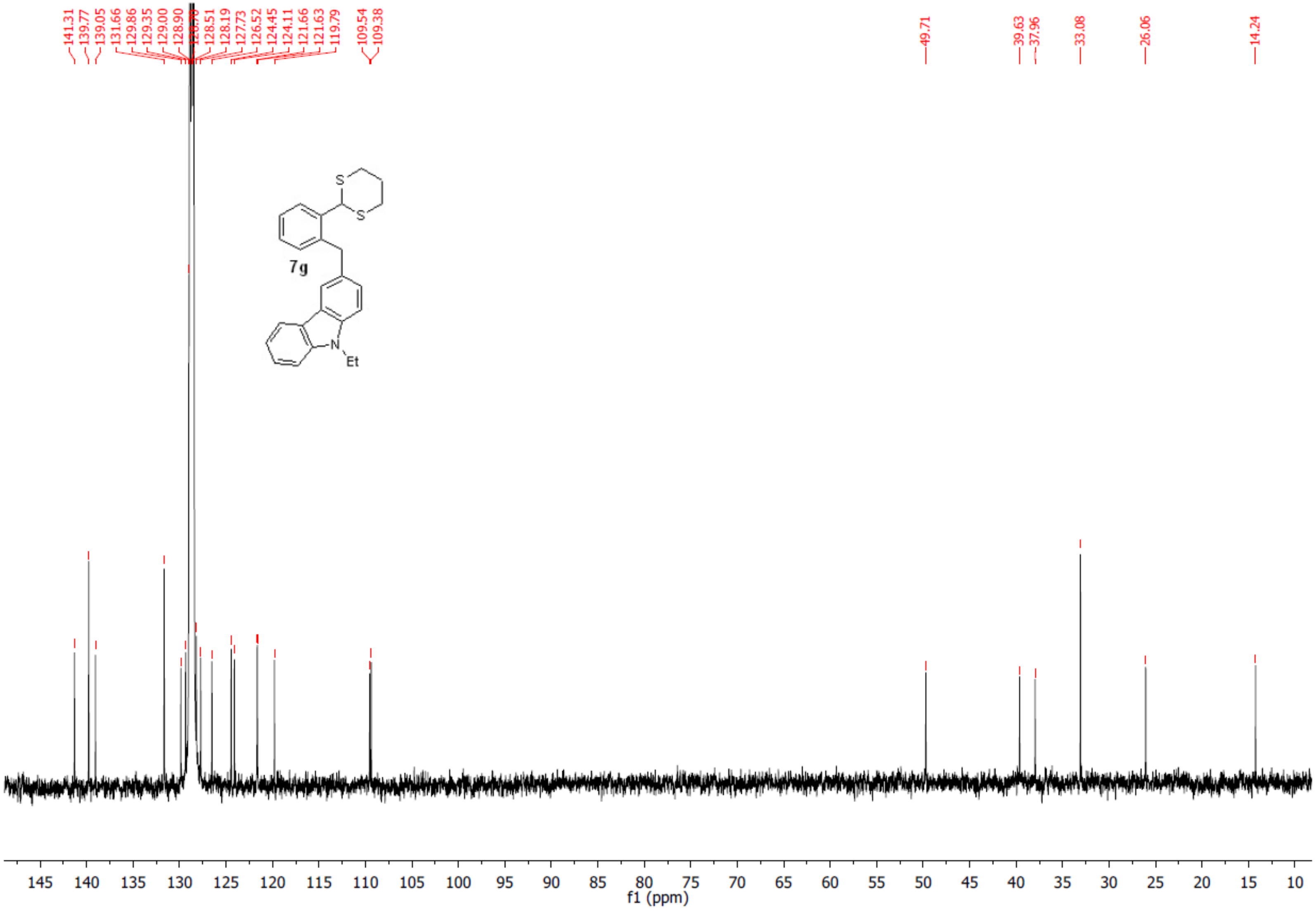

^{1}H NMR spectrum of 2-(2-(4-methoxybenzyl)phenyl)-1,3-dithiane (7h) (500 MHz, C_6D_6).

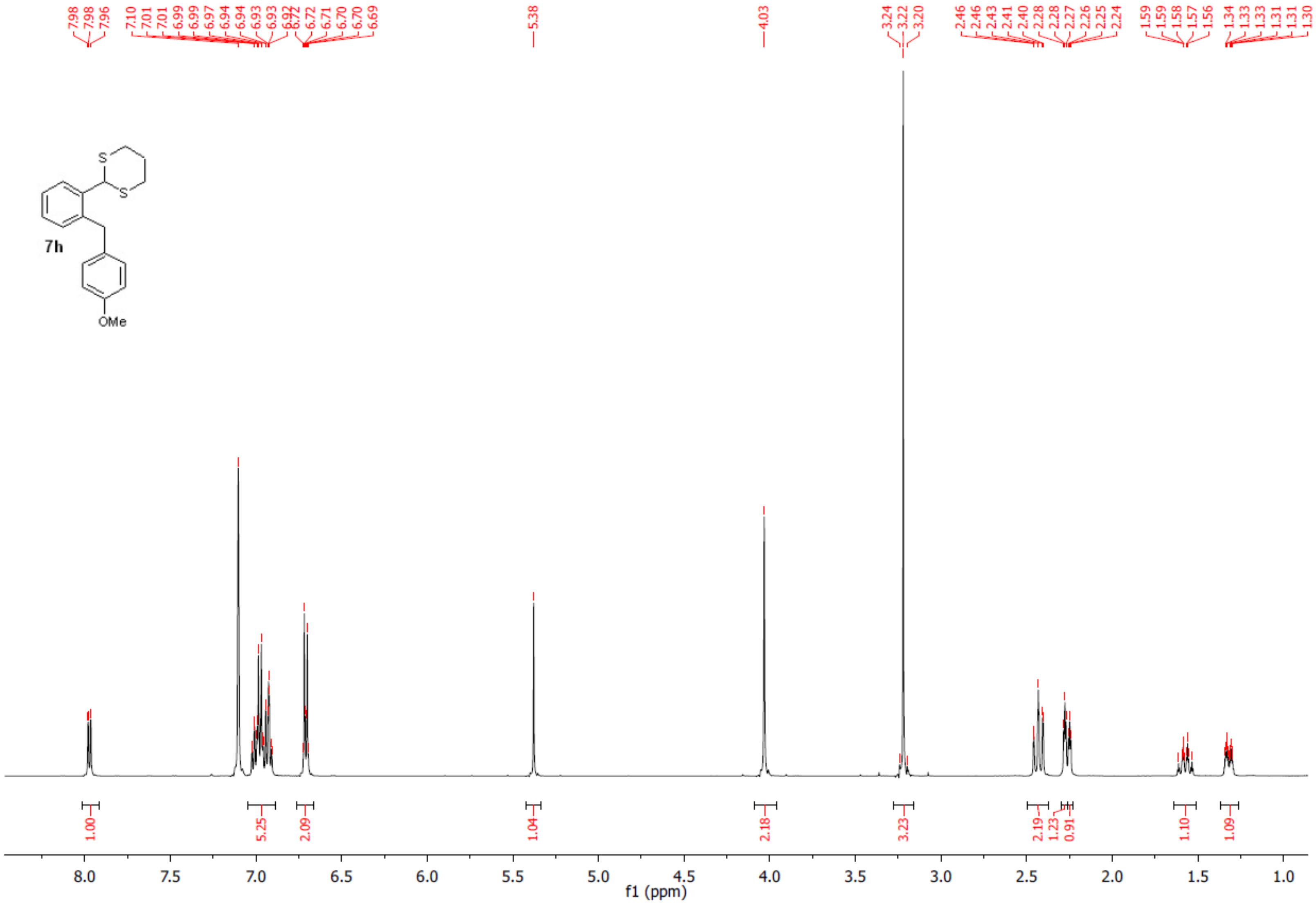

^{13}C NMR spectrum of 2-(2-(4-methoxybenzyl)phenyl)-1,3-dithiane (7h) (125 MHz, C_6D_6).

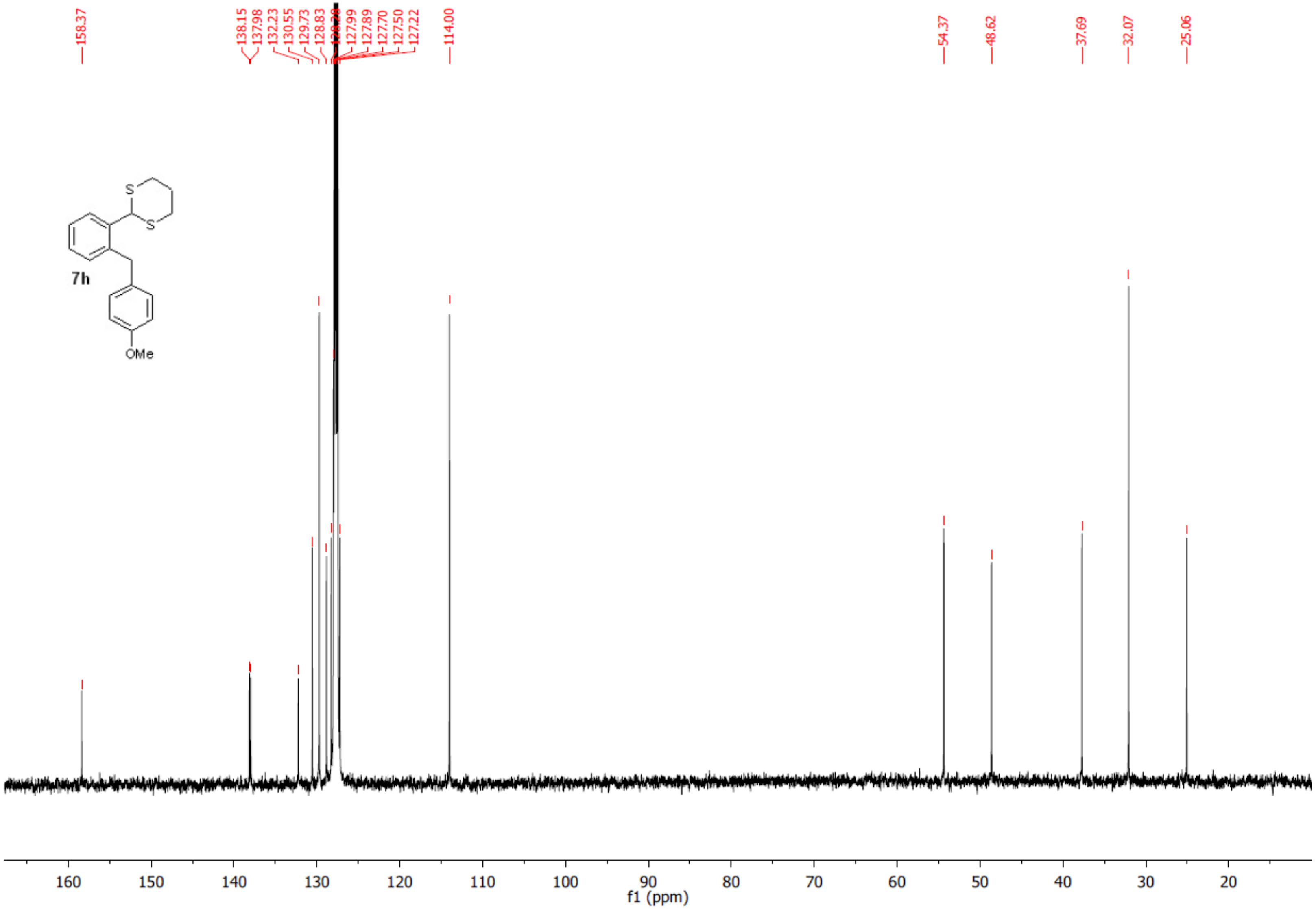

^{1}H NMR spectrum of 5-(benzo[*d*][1,3]dioxol-5-ylmethyl)-6-(1,3-dithian-2-yl)benzo[*d*][1,3]dioxole (8a) (500 MHz, C_6D_6).

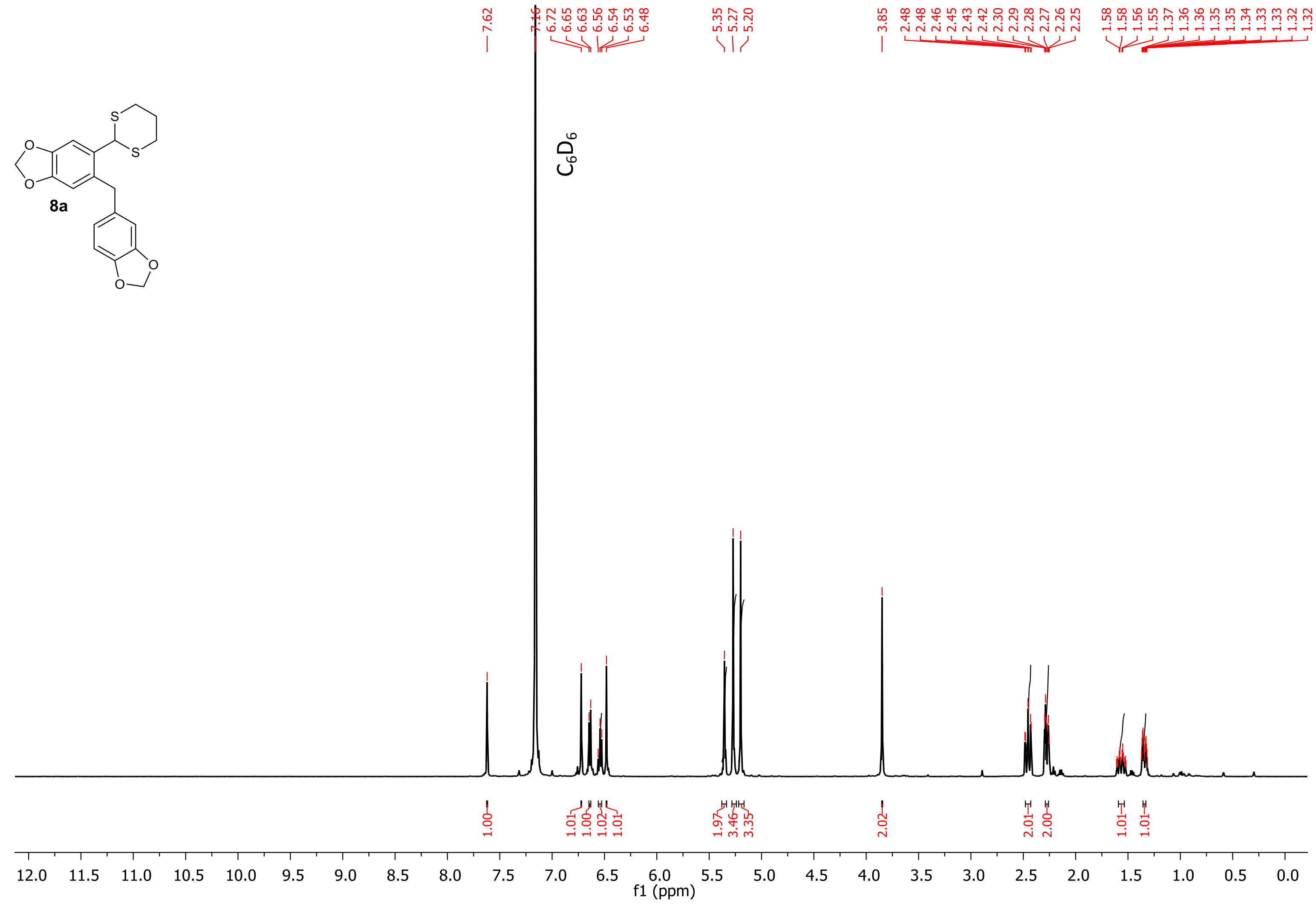

^{13}C NMR spectrum of 5-(benzo[*d*][1,3]dioxol-5-ylmethyl)-6-(1,3-dithian-2-yl)benzo[*d*][1,3]dioxole (8a) (125 MHz, C_6D_6).

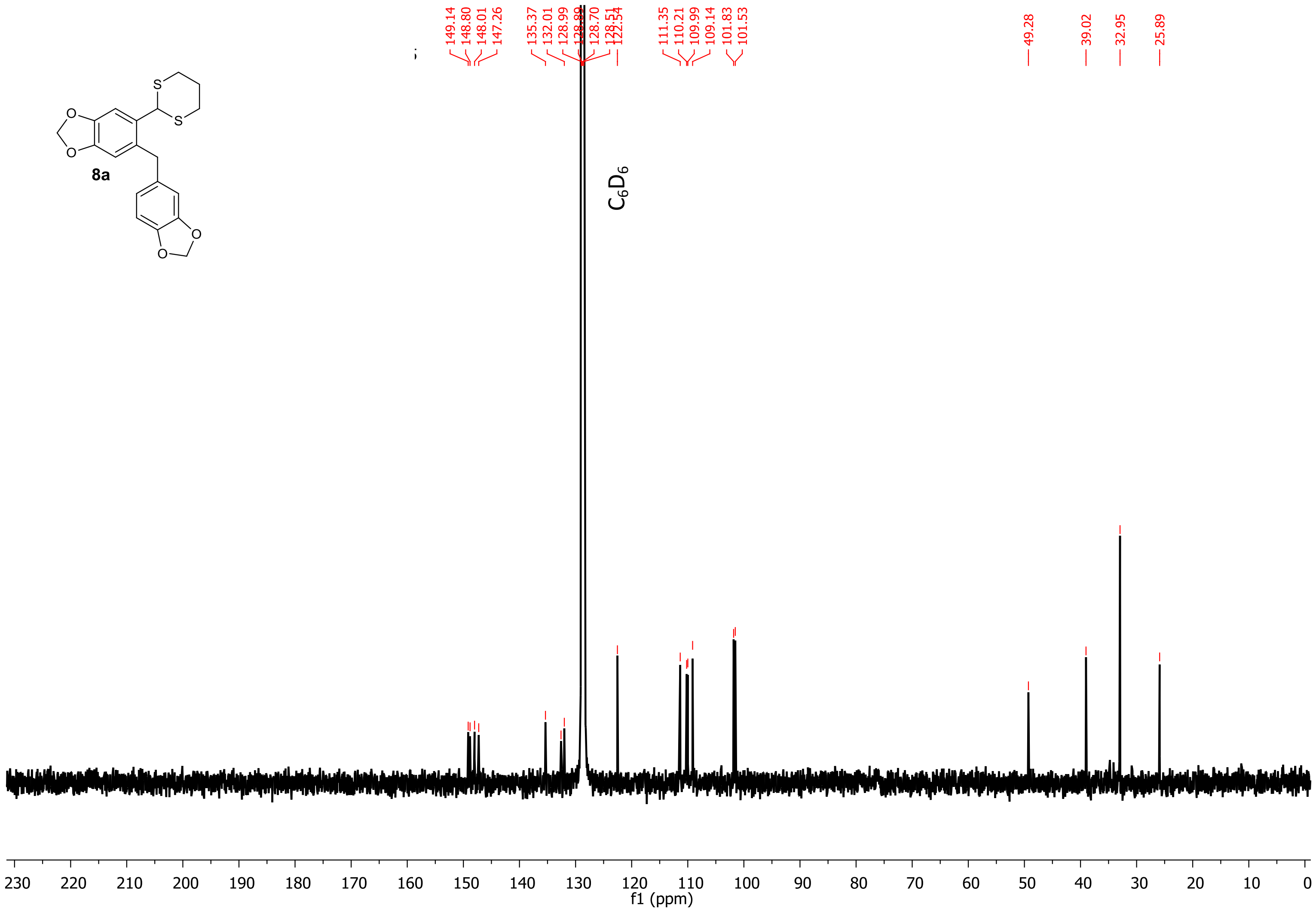

^{1}H NMR spectrum of 5-(1,3-dithian-2-yl)-6-(3,4,5-trimethoxybenzyl)benzo[*d*][1,3]dioxole (8b) (500 MHz, C_6D_6).

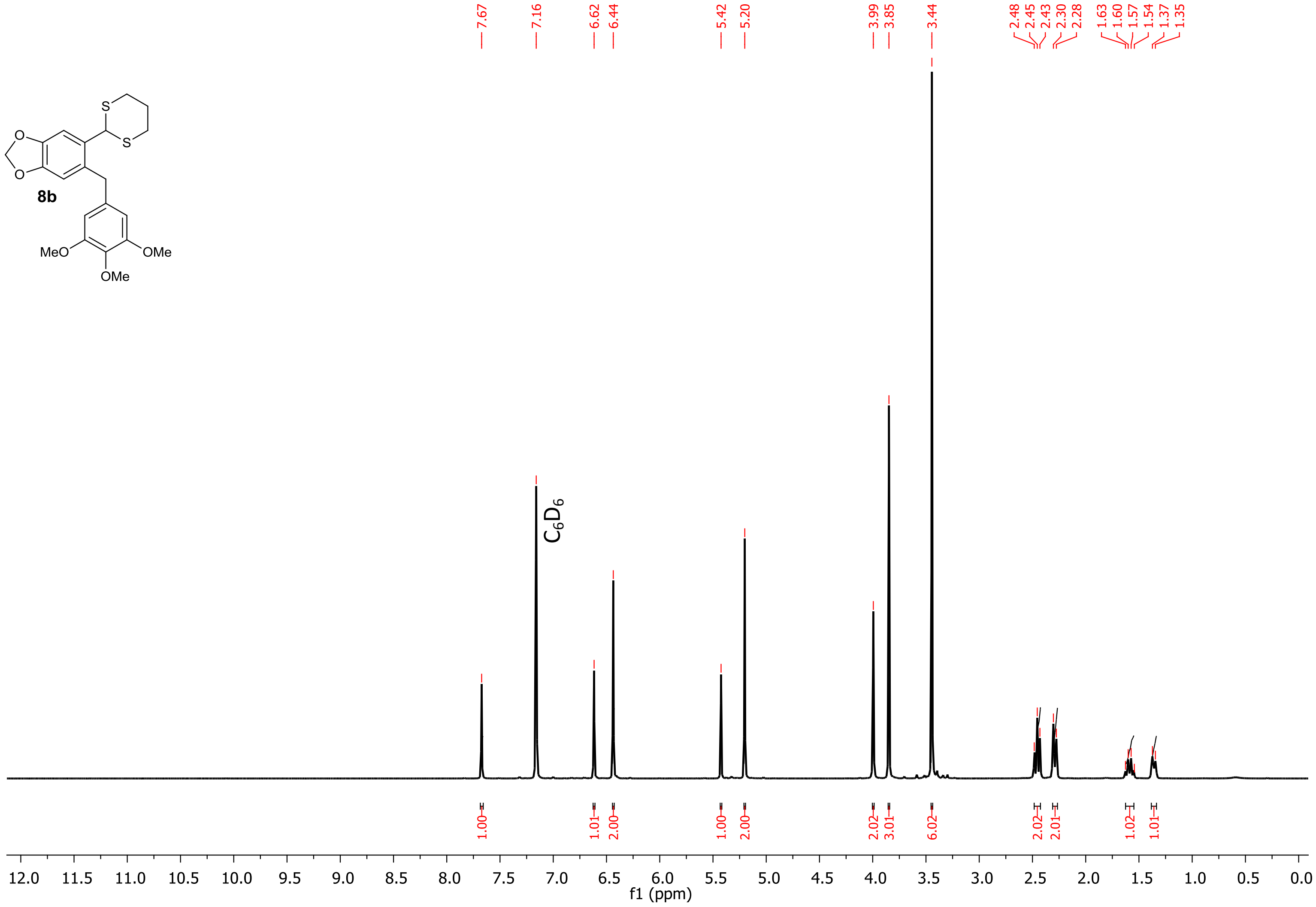

^{13}C NMR spectrum of 5-(1,3-dithian-2-yl)-6-(3,4,5-trimethoxybenzyl)benzo[*d*][1,3]dioxole (8b) (125 MHz, C_6D_6).

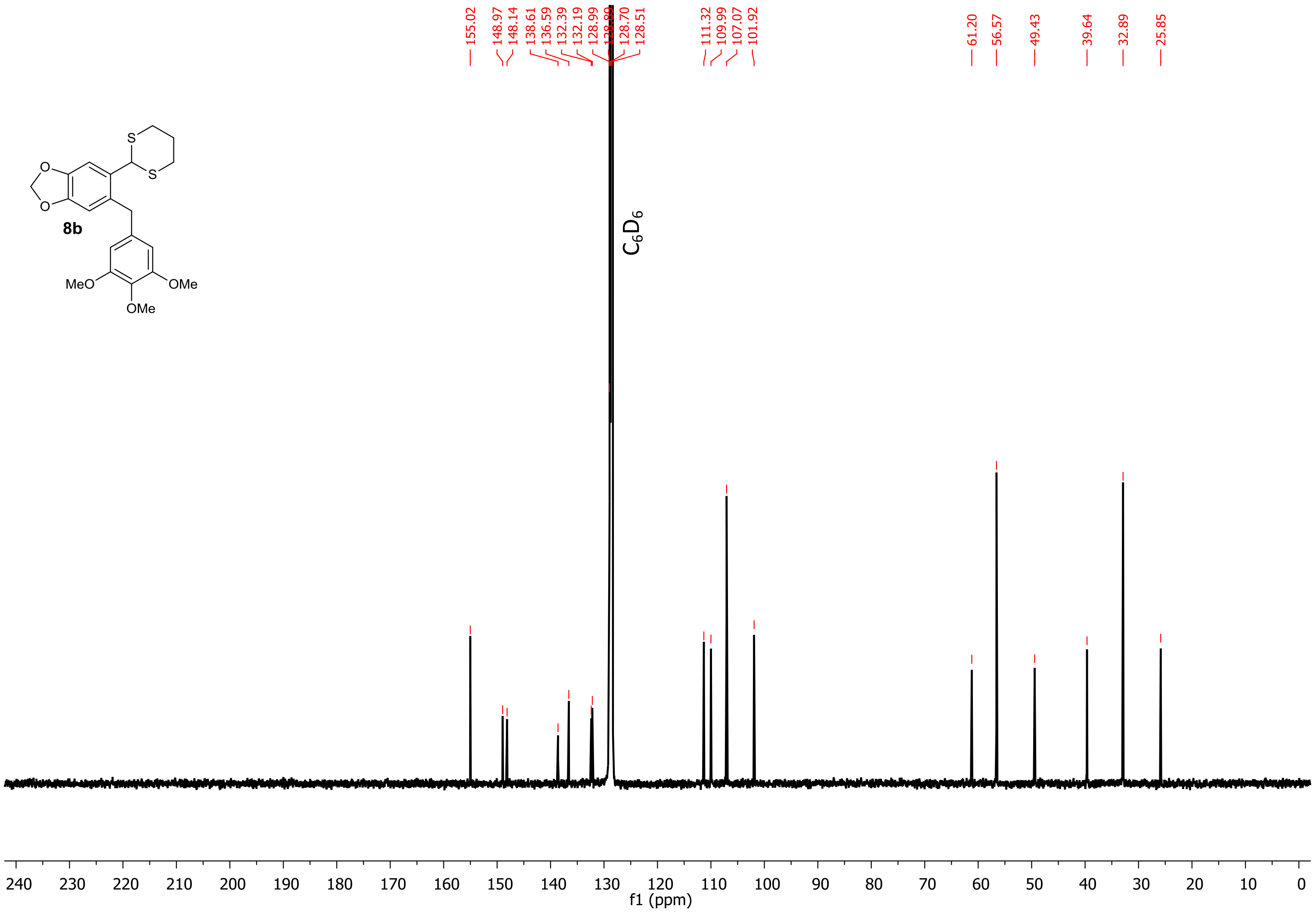

^{1}H NMR spectrum of 5-(3,4,5-trimethoxy-phenyl)-5,7-dihydrofuro[3',4':4,5]benzo[1,2-d][1,3]dioxole (10) (200 MHz, C_6D_6).

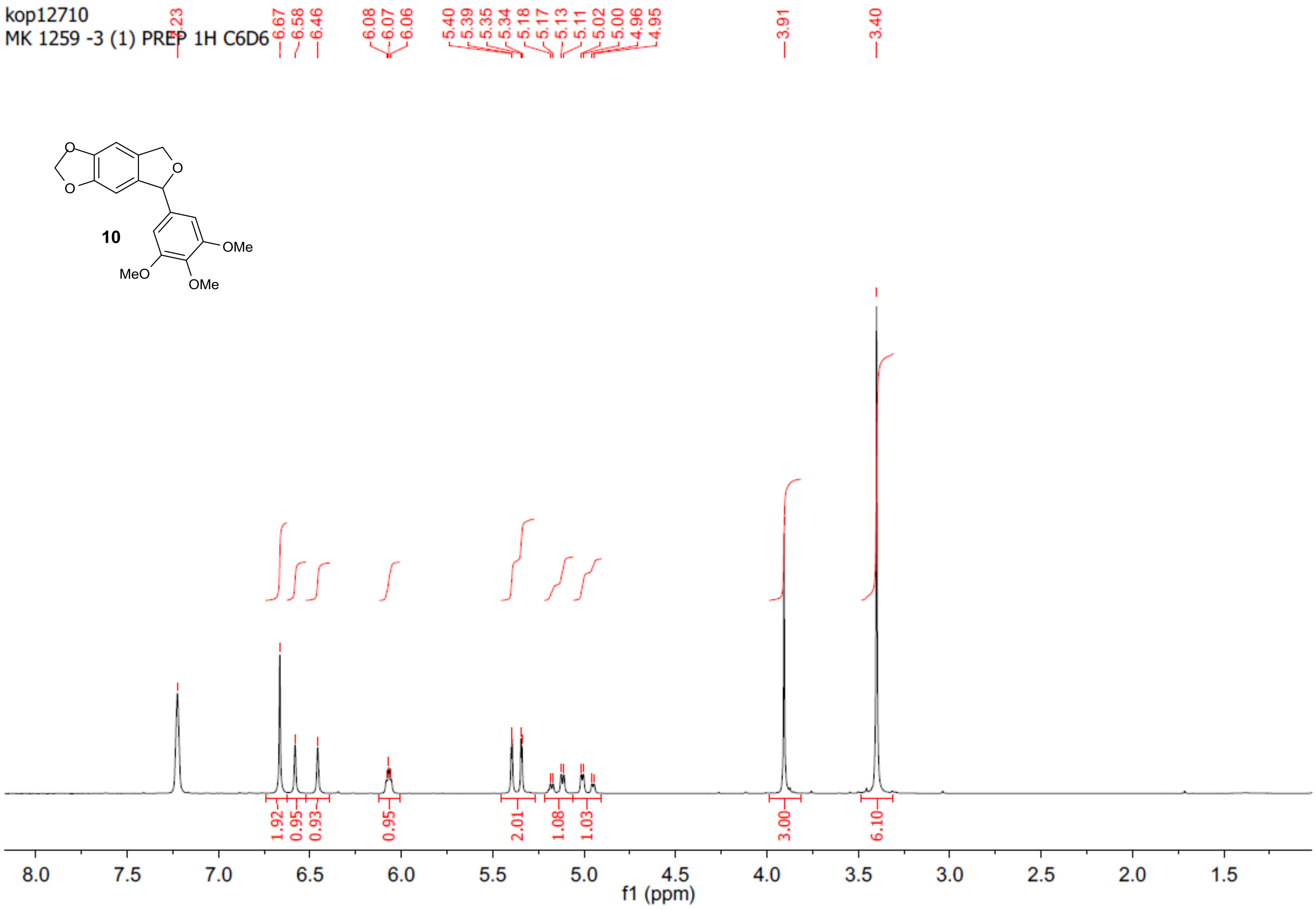

^{1}H NMR spectrum of 5-(3,4,5-trimethoxy-phenyl)-5,7-dihydrofuro[3',4':4,5]benzo[1,2-d][1,3]dioxole (10) (500 MHz, CD_2Cl_2).

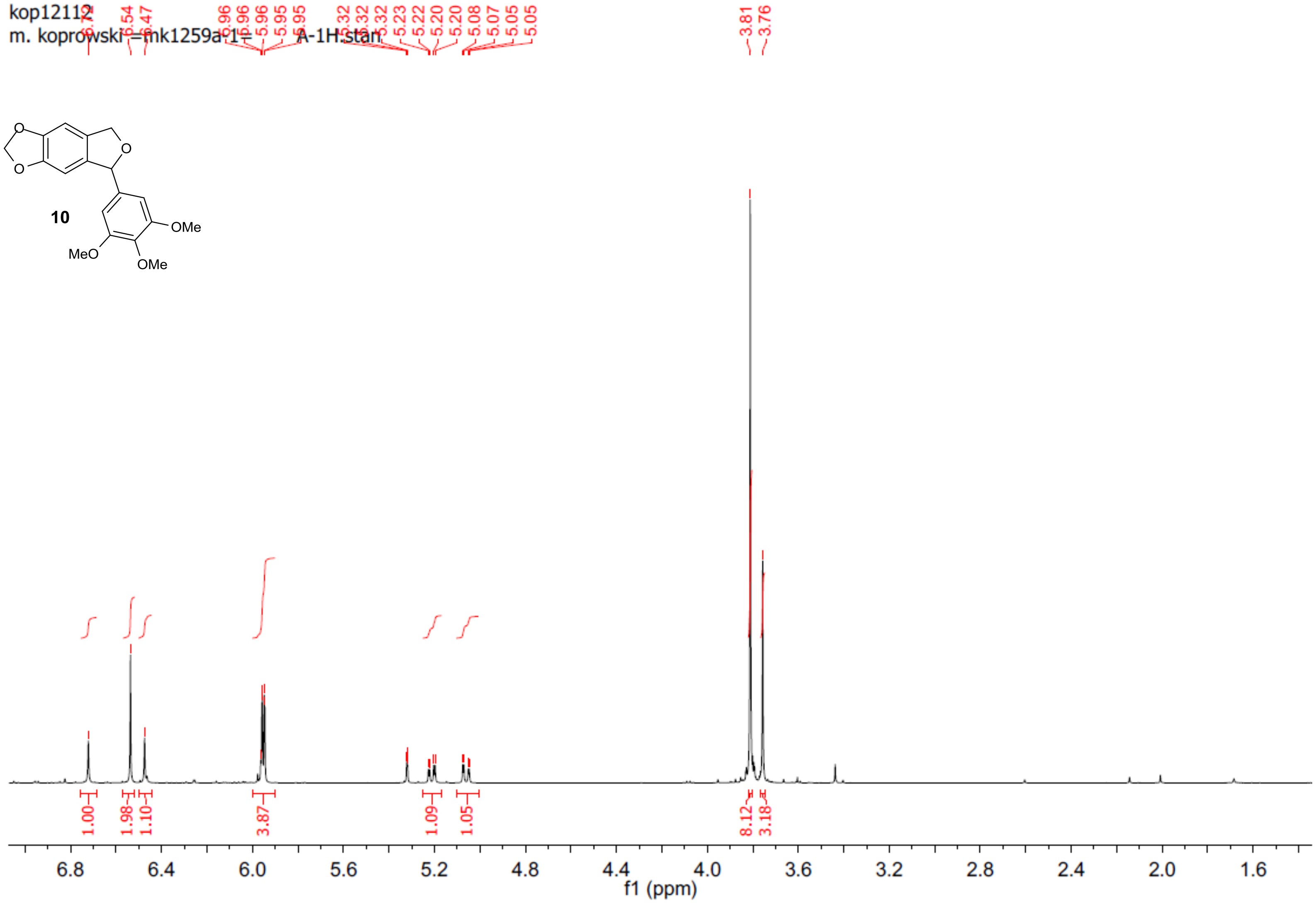

^{13}C NMR spectrum of 5-(3,4,5-trimethoxyphenyl)-5,7-dihydrofuro[3',4':4,5]benzo[1,2-d][1,3]dioxole (10) (500 MHz, CD_2Cl_2).

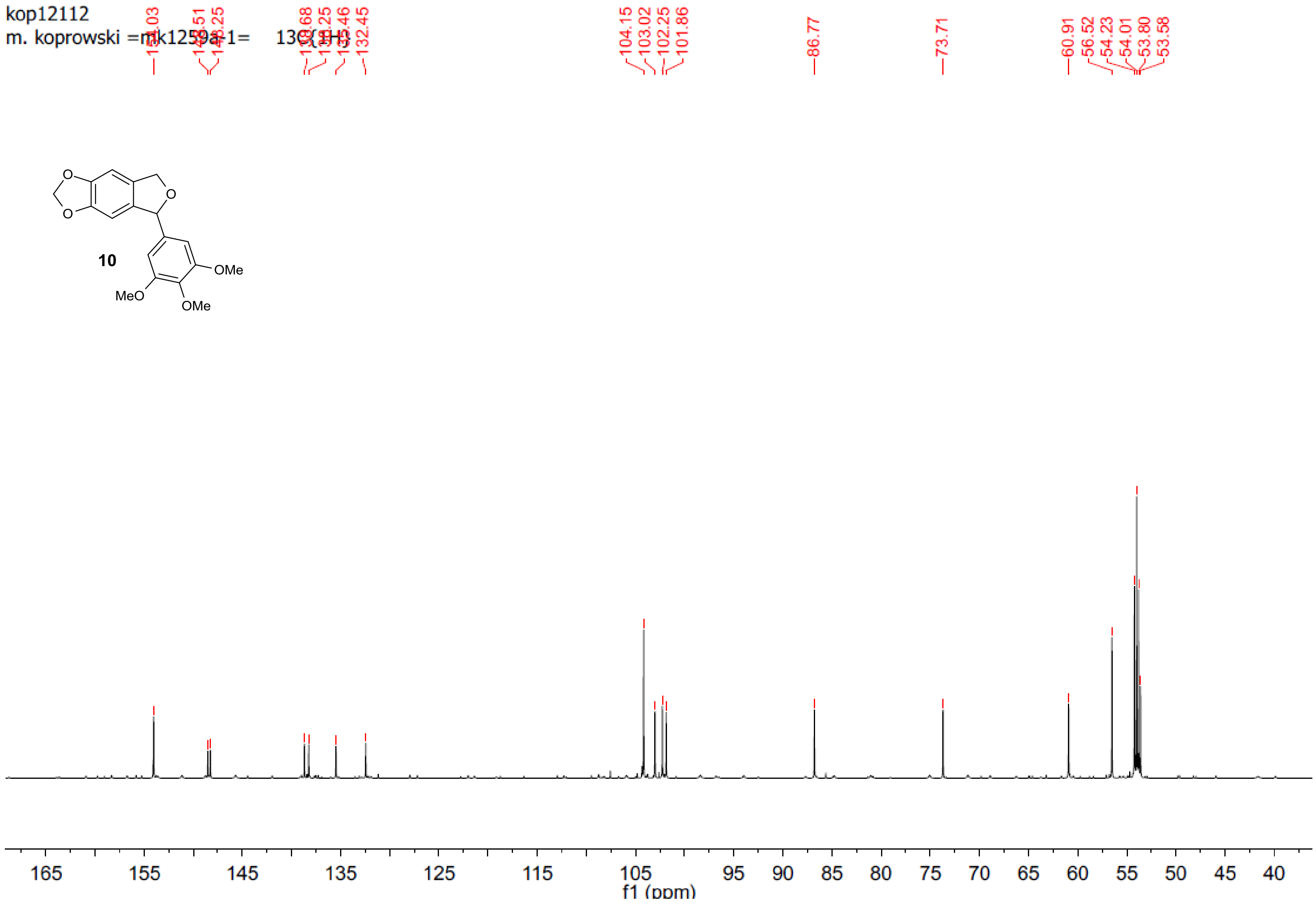

Supplement: File 1 — General experimental information, characterization data and copies of 1H, 13C NMR spectra. [file Beilstein_J_Org_Chem-14-1229-s001.pdf]
